# TGF-β in chronic kidney disease: a controversy for decades

**DOI:** 10.3389/fimmu.2026.1921148

**Published:** 2026-10-05

**Authors:** Heng Wang, Jie Tian, Guoping Zheng, Yun Zhou

**Affiliations:** 1 Centre for Transplant and Renal Research, Westmead Institute for Medical Research, The University of Sydney, Sydney, NSW, Australia; 2 Faculty of Medicine and Health, The University of Sydney, Camperdown, NSW, Australia; 3 Department of Pharmacy, The First Hospital of Shanxi Medical University, Taiyuan, Shanxi, China; 4 Department of Nephrology, The First Hospital of Shanxi Medical University, Taiyuan, Shanxi, China

**Keywords:** chronic kidney disease, fibrosis, immunology, therapy, transforming growth factor-beta

## Abstract

Chronic kidney disease (CKD) is characterized by persistent inflammation, maladaptive repair and progressive renal fibrosis, yet therapeutic strategies capable of effectively targeting these interconnected processes remain limited. Transforming growth factor-β1 (TGF-β1) is a central mediator of renal fibrosis but also exerts essential immunoregulatory and tissue-protective functions, creating a fundamental therapeutic paradox. Emerging evidence indicates that these divergent effects are determined not simply by TGF-β1 abundance, but by its cellular source and target, spatial and temporal context, interactions with other signaling pathways, and the transcriptional cofactors that determine downstream gene expression. This context dependence may partly explain why systemic TGF-β neutralization has shown limited clinical efficacy despite compelling preclinical evidence. In this Review, we discuss the cell-specific and stage-dependent functions of TGF-β1 in CKD, controversies surrounding the cellular origins of renal myofibroblasts, and the integration of TGF-β signaling with some signaling pathways. We further examine lessons from clinical trials and emerging therapeutic approaches designed to selectively modulate pathological TGF-β signaling, including regulation of latent TGF-β activation, receptor and Smad signaling, transcriptional cofactors, tissue-targeted delivery, and gene- and cell-based strategies. Particular attention is given to transcriptional partner selection as a mechanism of signal reprogramming. The β-catenin–TCF/FOXO1 axis provides a proof of concept whereby redirecting β-catenin from profibrotic TCF-dependent transcription toward FOXO1-associated anti-inflammatory and reparative programs may preserve beneficial TGF-β functions while limiting fibrosis. We propose that future TGF-β-targeted therapy should move beyond global pathway inhibition toward spatially, temporally and molecularly informed signal reprogramming, supported by biomarker-guided patient stratification and emerging single-cell and spatial technologies. Such precision modulation may provide a more rational strategy to suppress pathological fibrosis while preserving immune homeostasis and regenerative capacity in CKD.

## 1 Introduction

CKD represents a major global health burden, affecting approximately 850 million individuals worldwide with a prevalence of 14.2%, and is projected to become the fifth leading cause of death by 2050 (1, 2). The defining pathological feature of CKD is progressive renal fibrosis leading to the gradual loss of kidney function and eventual renal failure (3–5). Among the many mediators implicated in fibrogenesis, transforming growth factor-β1 (TGF-β1) is recognized as one of the most potent profibrotic cytokines (6). Across diverse fibrotic conditions, including CKD, diabetic nephropathy (DN), cardiac and hepatic fibrosis, pulmonary arterial hypertension, and atherosclerosis, TGF-β1 promotes tissue fibrosis primarily through the induction of mesenchymal transition processes (7–10). Preclinical studies targeting TGF-β signaling have demonstrated promising antifibrotic effects in various animal models (11, 12). However, as a multifunctional cytokine, TGF-β1 also plays essential roles in regulating cell growth and development, immune surveillance, and tissue repair (13, 14). Due to these context-dependent and sometimes contradictory effects, TGF-β1 signaling may either attenuate or exacerbate disease progression and thus presents challenges as a therapeutic target. Notably, clinical trials evaluating anti-TGF-β therapies in patients with CKD have shown limited or inconsistent benefits in terms of antifibrotic efficacy and renal functional improvement (15–18). A central unresolved question is therefore how TGF-β1 signaling gives rise to such divergent biological outcomes and whether its pathological profibrotic effects can be selectively uncoupled from its anti-inflammatory and reparative functions.

Although all three mammalian TGF-β isoforms (TGF-β1, TGF-β2 and TGF-β3) signal through overlapping receptor and Smad pathways, this Review focuses principally on TGF-β1 because it is the most extensively characterized isoform in renal inflammation and fibrosis and has provided the predominant mechanistic and therapeutic evidence in CKD. TGF-β2 and TGF-β3 may also contribute to renal development, homeostasis and disease, but their cell-specific functions and therapeutic relevance in CKD are considerably less well defined. The canonical TGF-β/Smad signaling pathway has been well characterized. Substantial evidence indicates that TGF-β1 activates downstream effectors such as Smad3 and Smad3-dependent non-coding RNAs, thereby transcriptionally regulating renal inflammation and fibrosis. In contrast, Smad7 acts as a negative regulator of these pathological processes (6). Rebalancing Smad3/Smad7 signaling or selectively targeting Smad3-dependent non-coding RNAs that modulate renal fibrosis or inflammation has therefore emerged as a promising therapeutic strategy (19, 20). However, Smad proteins alone possess low intrinsic affinity for DNA and cannot specify target gene binding sites independently. Instead, DNA-binding specificity is determined by complexes formed between Smads and transcriptional cofactors (21). At the same time, TGF-β signaling is extensively integrated with Wnt/β-catenin, MAPK, integrin–ILK, PI3K–Akt–mTOR, EGFR, Notch and BMP7 pathways. These interactions can amplify, attenuate or qualitatively redirect downstream TGF-β responses according to cellular and disease context. Thus, the biological output of TGF-β1 is determined not simply by whether the pathway is activated, but by where and when signaling occurs and how its downstream transcriptional machinery is configured.

β-catenin provides an illustrative example of context-dependent transcriptional regulation. In addition to its canonical role in Wnt signaling, β-catenin interacts with Smad-associated transcriptional machinery and with alternative nuclear partners, including T-cell factor (TCF) and forkhead box O1 (FOXO1). Preferential association of β-catenin with TCF is linked to profibrotic transcriptional programs, whereas increased interaction with FOXO1 can favor anti-inflammatory and reparative responses. The balance between β-catenin–TCF and β-catenin–FOXO1 transcriptional activity therefore provides a proof of concept that pathological TGF-β1 signaling can potentially be redirected at the transcriptional level without complete suppression of upstream signaling.

In this Review, we critically examine the profibrotic, immunoregulatory and tissue-protective functions of TGF-β1 in CKD, with particular emphasis on cell-specific responses, the evolving controversy surrounding the cellular origins of renal myofibroblasts, and the spatial and temporal heterogeneity of TGF-β1 signaling. We further discuss its crosstalk with major fibrotic and reparative pathways, evaluate lessons from clinical trials of TGF-β–targeted therapies, and compare emerging strategies for more selective therapeutic modulation. Finally, we propose that future therapeutic development should move beyond global TGF-β inhibition toward context-dependent signal reprogramming, integrating molecular selectivity, tissue and cell specificity, therapeutic timing and biomarker-guided patient stratification to suppress pathological fibrosis while preserving immune homeostasis and renal repair.

## 2 Overview of TGF-β signaling pathways

The TGF-β family of peptides belongs to the larger superfamily of growth factors and is encoded by 33 genes in mammals. These peptides function as either homodimers or heterodimers. In both humans and mice, 33 TGF-β family members have been identified, including three TGF-β isoforms, inhibins (activins), nodal proteins, bone morphogenetic proteins (BMP), and growth and differentiation factors (GDF) (22). Among these, TGF-β1 is the most well-characterized isoform with a clearly established profibrotic role in various disease models. Studies on the latent and activation mechanisms of TGF-β signaling have primarily focused on TGF-β1 (23). The TGF-β precursor consists of an N-terminal latency-associated peptide (LAP) and a C-terminal mature TGF-β domain (24). Within the rough endoplasmic reticulum, two precursor molecules are assembled into a homodimer via three disulfide bonds. The LAP wraps around the mature TGF-β domain, forming a latent complex that maintains TGF-β in an inactive state (25, 26). Three major mechanisms have been described for the release of active TGF-β: Proteolytic cleavage of the LAP domain by extracellular proteases (24); Covalent cross-linking of the LAP homodimer to latent TGF-β binding proteins (LTBP), forming a large latent complex (LLC) that becomes sequestered within the extracellular matrix (ECM) and is mechanically activated by integrin-mediated cellular traction (25, 27); Membrane anchoring of the latent complex by glycoprotein A repetitions predominant (GARP), which facilitates activation of TGF-β via integrin-dependent mechanisms (28, 29). Taken together, the release of bioactive TGF-β is a tightly regulated and multifaceted process occurring in the extracellular space, ensuring spatial and temporal control of its signaling activity.

The TGF-β signaling cascade is a prototypical membrane-to-nucleus pathway, encompassing both canonical (Smad-dependent) and non-canonical (Smad-independent) branches. The molecular events underlying TGF-β signaling have been extensively reviewed; here, we provide a brief overview (**Figure 1**).

**Figure 1:**
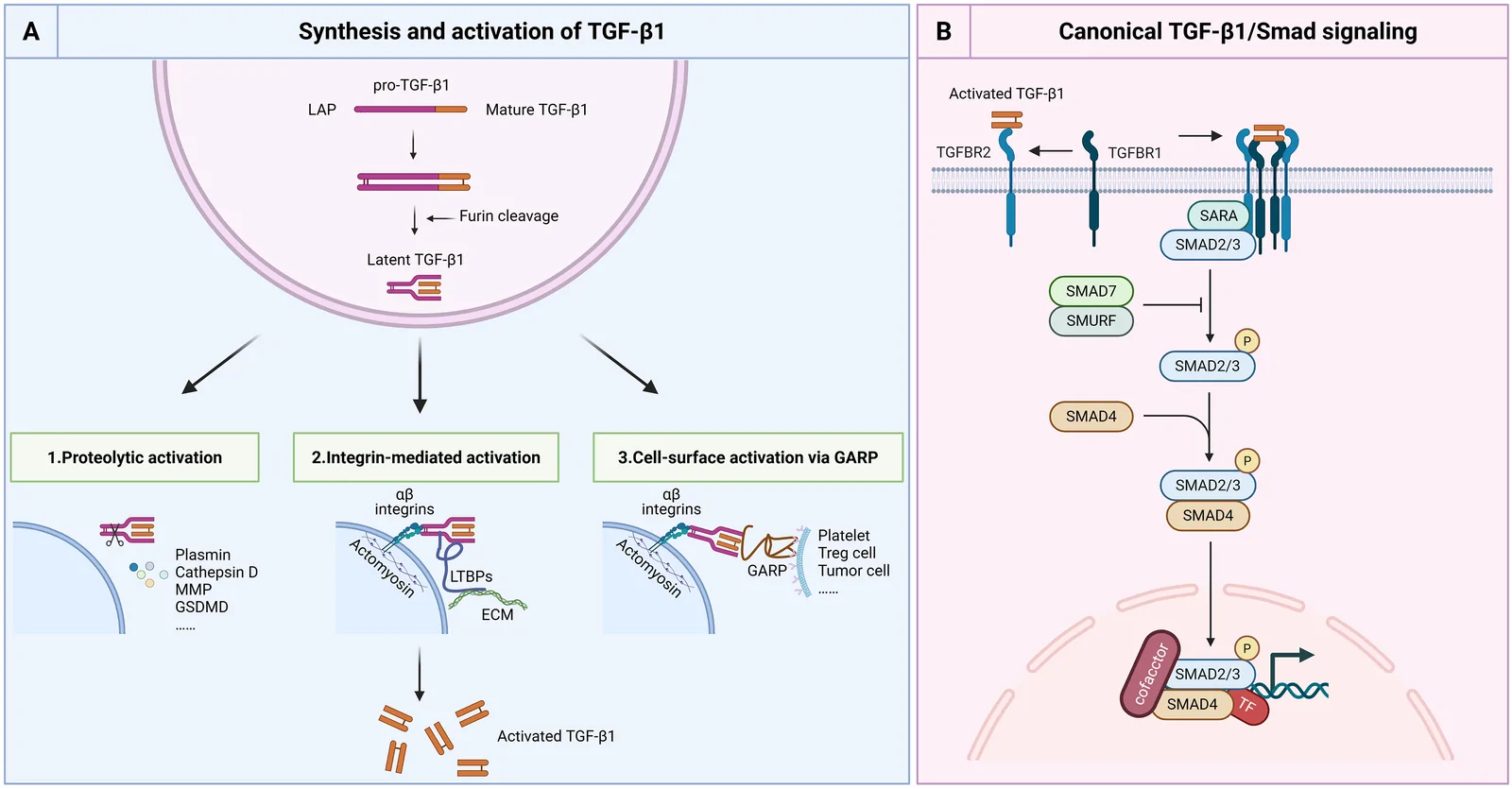
Synthesis, activation, and canonical Smad signaling of TGF-β1. **(A)** Synthesis and activation of TGF-β1. TGF-β1 is synthesized as a precursor (pro-TGF-β1) containing the latency-associated peptide (LAP) and mature TGF-β1 domains. Following furin-mediated proteolytic cleavage, mature TGF-β1 remains non-covalently associated with LAP to form latent TGF-β1. Latent TGF-β1 can be sequestered within the extracellular matrix (ECM) through latent TGF-β-binding proteins (LTBPs) or tethered to the cell surface by glycoprotein A repetitions predominant (GARP). Bioactive TGF-β1 can subsequently be released through several mechanisms, including proteolytic cleavage, integrin-mediated mechanical activation involving cytoskeletal tension, and activation of GARP-associated latent TGF-β1 at the cell surface. These mechanisms enable spatially and contextually regulated liberation of mature TGF-β1 from its latent complex. **(B)** Canonical TGF-β1/Smad signaling. Activated TGF-β1 binds TGF-β receptor type II (TGFBR2), which recruits and activates TGF-β receptor type I (TGFBR1). The activated receptor complex phosphorylates receptor-regulated Smad2/3, facilitated by the Smad anchor for receptor activation (SARA). Phosphorylated Smad2/3 associates with Smad4 and translocates into the nucleus, where the complex cooperates with transcription factors (TFs) and transcriptional cofactors to regulate context-dependent target-gene expression. Smad7 negatively regulates canonical TGF-β signaling, in part through recruitment of Smad ubiquitination regulatory factors (SMURFs), thereby limiting receptor-mediated Smad2/3 activation. Created in BioRender.

The TGF-β superfamily signals through a set of seven type I and five type II serine/threonine kinase receptors. Specifically, TGF-β1 exerts its effects via TGF-β receptor type I (TGFBR1, also known as TβR-I or ALK5) and type II (TGFBR2, also known as TβR-II). The Smad family comprises eight members categorized into three functional classes: receptor-regulated Smads (R-Smad: Smad1, 2, 3, 5, and 8), the common-mediator Smad (Co-Smad: Smad4), and inhibitory Smads (I-Smad: Smad6 and 7). Upon ligand binding, active TGF-β1 interacts directly with TGFBR2, either independently or with the aid of a co-receptor such as betaglycan (also known as TGFBR3), leading to TGFBR2 autophosphorylation. TGFBR2 subsequently recruits and activates TGFBR1, resulting in the formation of a heteromeric receptor complex. Activated TGFBR1 phosphorylates Smad2 and Smad3 at their C-terminal serine residues, facilitating the formation of a heterotrimeric complex with Smad4. This complex translocates to the nucleus, where it cooperates with transcriptional cofactors to regulate the expression of target genes, constituting the canonical TGF-β1 signaling pathway.

In the non-canonical TGF-β signaling pathways, TGF-β elicits cellular responses independently of Smad proteins. Upon receptor activation, downstream cascades are initiated through a variety of post-translational modifications, including phosphorylation, acetylation, sumoylation, ubiquitination, and protein–protein interactions. Active TGF-β can stimulate multiple signaling modules, such as the mitogen-activated protein kinase (MAPK) pathways, Rho-like GTPase signaling, and the phosphoinositide 3-kinase (PI3K)/AKT pathway. Interestingly, these non-Smad signaling pathways may be activated or modulated by other endogenous ligands or inhibitors, and in many cases, they exert either synergistic or antagonistic effects on Smad-dependent signaling. This intricate network of pathway crosstalk contributes to the context-dependent and often unpredictable biological outcomes of TGF-β signaling under both physiological and pathological conditions.

In the resting state, Smad proteins are retained in the cytoplasm by the Smad anchor for receptor activation (SARA). Upon receptor-mediated phosphorylation, Smads dissociate from SARA and translocate to the nucleus, where they participate in transcriptional regulation. Notably, even phosphorylated Smads exhibit low intrinsic affinity for DNA. The recruitment of specific transcriptional cofactors is essential for Smads to bind their target DNA sequences. Thus, the DNA-binding specificity of Smad complexes, and consequently, the transcriptional outcomes of TGF-β signaling, is largely determined by the repertoire of interacting cofactors.

## 3 Profibrotic roles of TGF-β1

Renal fibrosis is characterized by excessive ECM accumulation and expansion of activated myofibroblasts because of sustained or maladaptive tissue repair (30). TGF-β1 is a central profibrotic mediator that promotes ECM production and remodeling, fibroblast activation, parenchymal cell injury, and mesenchymal-like phenotypic changes in multiple renal cell populations. These effects collectively establish a profibrotic microenvironment and promote progressive tissue scarring. However, the extent to which epithelial, endothelial, and immune cells undergo bona fide lineage conversion into myofibroblasts remains controversial (**Figure 2**; **Table 1**).

**Figure 2:**
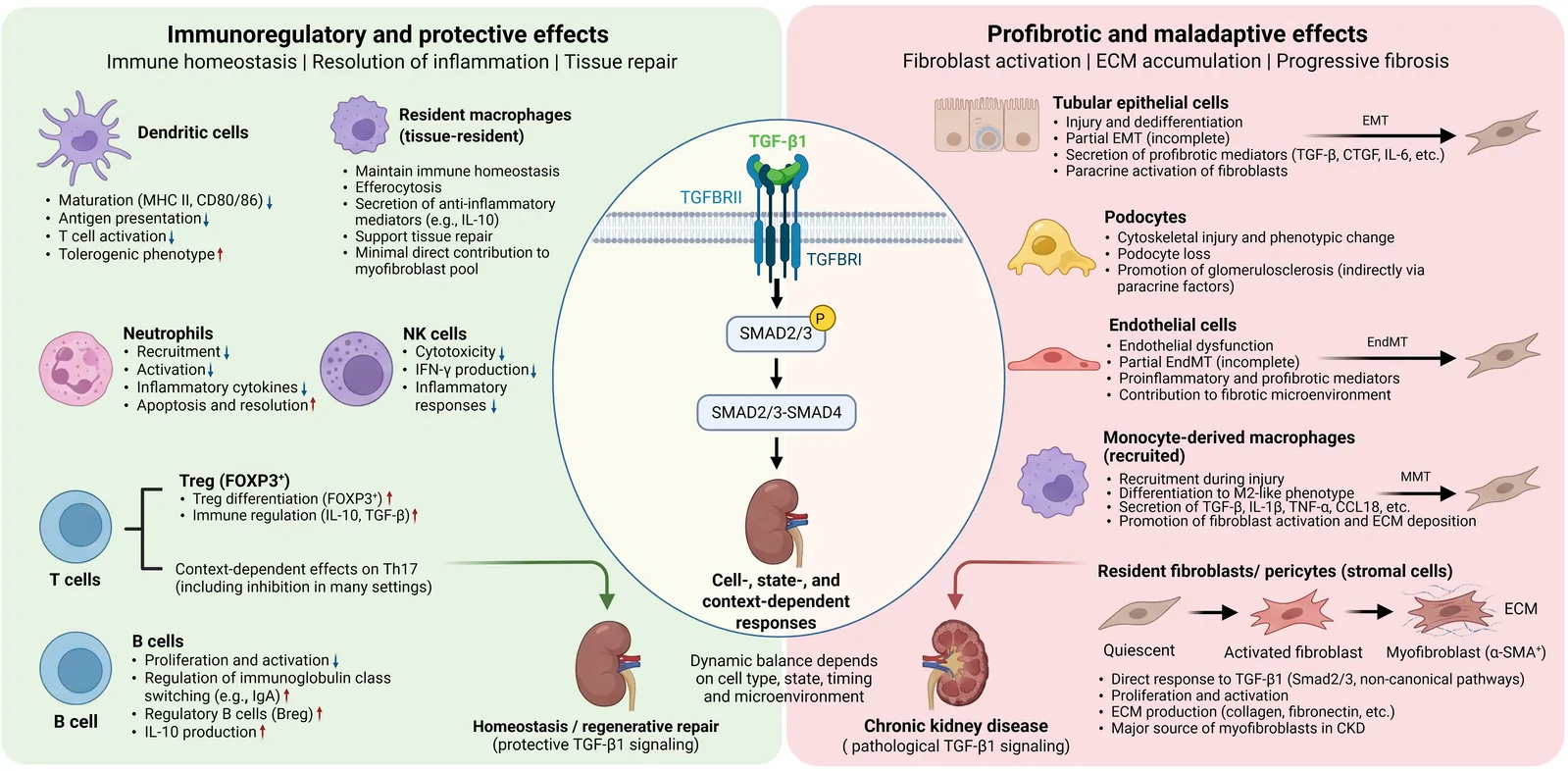
Cell- and context-dependent immunoregulatory, protective, and profibrotic effects of TGF-β1 in the kidney. TGF-β1 exerts divergent biological effects in kidney injury depending on the responding cell type, cellular state, timing, and local microenvironment. Following activation of TGFBR2 and TGFBR1, canonical signaling is mediated predominantly through phosphorylation of Smad2/3 and formation of Smad2/3–Smad4 complexes, although the ultimate cellular response is strongly shaped by cell-specific regulatory networks. Left: In homeostasis and regenerative repair, TGF-β1 can exert immunoregulatory and tissue-protective effects. It suppresses dendritic-cell maturation, antigen presentation, and T-cell activation; limits neutrophil recruitment, activation, and inflammatory cytokine production while facilitating inflammatory resolution; and restrains natural killer (NK)-cell cytotoxicity and inflammatory responses. TGF-β1 also promotes regulatory T-cell (Treg) differentiation and immune regulation and modulates B-cell activation, immunoglobulin class switching, and regulatory B-cell responses. Tissue-resident macrophages contribute to immune homeostasis, efferocytosis, anti-inflammatory mediator production, and tissue repair, with limited direct contribution to the myofibroblast pool. Right: During persistent or maladaptive injury, sustained TGF-β1 signaling promotes renal fibrogenesis. Tubular epithelial and endothelial cells acquire partial epithelial-to-mesenchymal transition (EMT)- and endothelial-to-mesenchymal transition (EndMT)-like phenotypes, respectively, and contribute to fibrosis predominantly through cellular dysfunction and paracrine profibrotic signaling rather than extensive complete lineage conversion. TGF-β1 also promotes podocyte injury and glomerular damage. Recruited monocyte-derived macrophages can acquire profibrotic phenotypes and secrete TGF-β1 and other mediators that activate stromal cells; macrophage-to-myofibroblast transition (MMT) has been proposed, although its quantitative contribution to the myofibroblast pool remains limited and debated. Resident fibroblasts and pericyte-like stromal cells represent major sources of matrix-producing myofibroblasts and respond directly to TGF-β1 by undergoing activation, proliferation, and extracellular matrix (ECM) production. Collectively, the balance between protective and pathological TGF-β1 signaling is dynamic rather than binary and shifts according to the cellular and spatiotemporal context of kidney injury. Created in BioRender.

**Table 1:** Cell-specific and context-dependent effects of TGF-β1 in CKD.

Major TGF-β1-associated signaling mechanisms	Major cellular responses	Predominant functional consequence	Context dependence	References
Smad2/3; PI3K–Akt–FOXO1; SNAIL; NPY–Y1R–p38/NF-κB; Smad3–STAT3	M2-like/reparative polarization, suppression of inflammatory activation and enhanced phagocytosis; prolonged stimulation may promote profibrotic macrophage states and myofibroblast-like transition	Protective or profibrotic	Short-term signaling can favor anti-inflammatory and reparative phenotypes, whereas sustained activation may promote fibrosis; resident and monocyte-derived macrophages show distinct responses	(68, 81, 82, 84, 86, 305)
β-catenin; STAT3; integrin αvβ8-dependent TGF-β activation; CCL20–CCR6	Suppression of differentiation and maturation; reduced T-cell stimulation and Th1/Th17-polarizing cytokines; induction of tolerogenic functions; context-dependent recruitment	Predominantly immunoregulatory	TGF-β can suppress adaptive immune activation but may also participate in DC recruitment within fibrotic niches	(96–101)
NETs–NF-κB–TGF-β1/Smad signaling; TGF-β-dependent regulation of inflammatory responses	Modulation of neutrophil recruitment and activation; NET-associated amplification of inflammatory and fibrotic signaling	Context dependent/potentially profibrotic	Effects depend on injury phase and the local inflammatory microenvironment	(102–105)
TGF-β–NKG2D axis	Reduced NK-cell recognition and cytotoxicity toward renal tubular epithelial cells	Predominantly tissue protective in renal injury	Suppression of cytotoxicity may protect injured tubular cells, although broader TGF-β-mediated NK suppression can be immunosuppressive	(145)
Smad-dependent signaling; FOXP3-associated regulatory programs; regulation of Th17/Treg balance	Promotion of Treg differentiation and immune tolerance; suppression of excessive effector responses	Predominantly immunoregulatory	Outcome depends strongly on T-cell subset and surrounding cytokine environment	(142, 143)
TGF-β-dependent regulatory signaling; Treg–B-cell interactions; GARP-associated TGF-β signaling	Suppression of B-cell activation; regulation of antibody responses; support of regulatory B-cell functions	Predominantly immunoregulatory	Effects vary among activated, antibody-producing and regulatory B-cell populations; kidney-specific mechanistic evidence remains relatively limited	(142–144)
Smad2/3; MAPK; PI3K–Akt; Wnt/β-catenin; Notch; mTOR; NKG2D-ligand regulation	Partial EMT and profibrotic secretory phenotype; regulation of mitochondrial integrity, inflammation and immune recognition	Protective or profibrotic	Intrinsic TGF-β signaling may protect mitochondrial homeostasis and limit inflammation, whereas persistent activation promotes maladaptive repair and fibrosis	(78, 145–147, 306)
Smad2/3; MAPK; Notch; Wnt/β-catenin	Cytoskeletal injury, apoptosis and EMT-like/mesenchymal changes	Predominantly pathogenic when sustained	Chronic TGF-β1 signaling contributes to podocyte dysfunction and glomerular injury	(33, 34, 37)
Smad2/3; integrin-dependent signaling; DPP-4; C/EBPβ	EndMT-like plasticity, endothelial dysfunction and profibrotic signaling	Predominantly profibrotic when sustained	Complete endothelial-to-myofibroblast conversion appears limited; partial phenotypic changes and paracrine signaling may be more important	(46, 80, 83)
Smad2/3; GSK3β; PI3K–Akt–mTOR; metabolic reprogramming	Myofibroblast activation, proliferation, collagen synthesis and ECM accumulation	Profibrotic	Resident stromal populations are currently considered the predominant source of matrix-producing myofibroblasts	(46, 79, 80, 83)
Smad2/3; ERK; PKCδ; TGF-β1–Smad2/3–YAP; NOX4	ECM production, cellular senescence and mesangial expansion	Predominantly profibrotic	Particularly relevant in glomerular and metabolic kidney injury	(52, 54, 56, 59, 283, 284)

### 3.1 Epithelial-to-mesenchymal transition

TGF-β1 can induce epithelial-to-mesenchymal transition (EMT)-like changes in renal tubular epithelial cells (TECs) and podocytes, characterized by loss of epithelial features and acquisition of mesenchymal and profibrotic phenotypes (31–37). Mechanistically, Smad3 is a major mediator of TGF-β1-induced EMT, while non-canonical pathways, including MAPK, PI3K, Wnt/β-catenin, Notch, and mTOR signaling, further modulate this response. However, lineage-tracing studies have challenged the concept that epithelial cells extensively convert into interstitial myofibroblasts. Instead, injured TECs may undergo partial EMT, retaining their epithelial identity while acquiring profibrotic properties that promote fibroblast activation through paracrine signaling. Thus, EMT is increasingly regarded as a spectrum of epithelial plasticity rather than necessarily representing complete epithelial-to-myofibroblast conversion.

### 3.2 Endothelial-to-mesenchymal transition

TGF-β1 can induce endothelial-to-mesenchymal transition (EndMT), characterized by loss of endothelial features and acquisition of mesenchymal and profibrotic phenotypes (38–40). TGF-β1/Smad2/3 signaling is a major driver of this process, as endothelial-specific deletion of TGFBR2 or inhibition of Smad2/3 attenuates EndMT and renal fibrosis in experimental models (40–42). Additional regulators include integrin β1 and dipeptidyl peptidase-4 (DPP-4), which modulate TGF-β receptor complex formation and Smad3 activation, as well as midkine–C/EBPβ-dependent profibrotic transcription (43–45). However, as with EMT, the quantitative contribution of bona fide endothelial-to-myofibroblast conversion to the renal myofibroblast pool remains debated and should be distinguished from EndMT-like endothelial plasticity and paracrine profibrotic signaling.

### 3.3 Activation of fibroblasts

In the healthy kidney, fibroblast populations primarily include interstitial fibroblasts, glomerular mesangial cells, and pericytes—each of which is considered a major contributor to the myofibroblast pool within the fibrotic microenvironment (46). TGF-β1 is a potent stimulator that drives the activation of quiescent interstitial fibroblasts into contractile and ECM-producing myofibroblasts (47). This fibroblast-to-myofibroblast transition is primarily mediated by the TGF-β1/Smad3 signaling pathway, in which glycogen synthase kinase 3β (GSK3β) also plays an essential regulatory role (48–50). Furthermore, secreted phosphoprotein 1 (SPP1) has been shown to enhance Smad2/3 phosphorylation and amplify downstream signaling (51). *In vitro*, TGF-β1 promotes mesangial cell senescence and ECM deposition via the Smad2/3 pathway (52, 53). Under high-glucose or elevated free fatty acid conditions, mesangial cells exhibit increased ERK and PKCδ activity, which interacts with the Smad pathway, leading to autocrine TGF-β1 production and enhanced responsiveness to TGF-β1 stimulation (54–56). Single-cell RNA sequencing data have identified a fibrogenic macrophage subset characterized by TGF-β1^+^Arg1^+^ expression. These macrophages activate the TGF-β1/Smad2/3/Yes-associated protein (YAP) axis, which drives fibrogenic changes in mesangial cells and contributes to the establishment of a pro-fibrotic renal microenvironment (57). The NAD(P)H oxidase isoform NOX4 plays a key role in reactive oxygen species (ROS)–mediated activation of both fibroblasts and mesangial cells and is closely associated with TGF-β1 signaling (58, 59). In models of DN, unilateral ureteral obstruction (UUO), and ischemia–reperfusion injury (IRI), TGF-β1/Smad3 signaling has been shown to promote pericyte-to-myofibroblast transition (PMT) (60, 61). Mechanistically, TGF-β1 represses genes involved in fatty acid oxidation and upregulates key glycolytic enzymes in pericytes, thereby promoting PMT through metabolic reprogramming (62). In particular, the PI3K–Akt–mTOR pathway enhances glycolysis via hexokinase II (HKII) activation to support the metabolic demands of PMT (63).

### 3.4 Macrophage-to-myofibroblast transdifferentiation

Macrophages play a pivotal role in the transition from renal inflammation to fibrosis (64, 65). Based on their functional phenotypes, macrophages are broadly categorized into pro-inflammatory (M1) and anti-inflammatory/pro-repair (M2) subtypes. M1 macrophages contribute to tissue injury through inflammatory cytokine release and matrix degradation. Notably, they also promote fibrosis indirectly by releasing MMPs, which degrade ECM components and activate latent TGF-β. Among these, MMP9 plays a crucial role in both EMT induction and the polarization of M2 macrophages. M2 macrophages, while anti-inflammatory, exert profibrotic effects and are considered a major cellular source of TGF-β1. Compared with M1 macrophages, M2 macrophages also exhibit greater potential to transdifferentiate into myofibroblasts (66, 67). Interestingly, the polarization of M1 to M2 macrophages and the subsequent macrophage-to-myofibroblast transition (MMT) appear to be temporally ordered events, both primarily driven by the TGF-β1/Smad3 signaling pathway (66, 68, 69). However, the quantitative contribution of MMT to the renal myofibroblast pool remains uncertain. Lineage-tracing and macrophage-depletion studies suggest that macrophages constitute only a limited proportion of scar-forming myofibroblasts and that their fibrogenic functions may predominantly arise from paracrine signaling rather than direct lineage conversion (46, 70, 71). Moreover, resident and monocyte-derived macrophages display distinct and context-dependent responses during kidney injury, further complicating interpretation of macrophage contributions to fibrosis (72–77). Thus, although TGF-β1/Smad3-driven MMT represents a biologically plausible profibrotic pathway, its relative contribution to the myofibroblast population remains debated and should be distinguished from the broader effects of macrophages on the fibrotic microenvironment.

### 3.5 Controversies over the cellular origins of myofibroblasts in the era of single-cell and spatial omics

The cellular origin of renal myofibroblasts has remained controversial because of their phenotypic plasticity and the lack of lineage-specific markers. Earlier studies proposed heterogeneous origins involving resident fibroblasts and pericytes, TECs undergoing EMT, endothelial cells undergoing EndMT, and bone marrow-derived cells undergoing MMT. One influential fate-mapping study estimated contributions of approximately 50% from resident fibroblasts, 35% from bone marrow-derived cells, 10% from endothelial cells and 5% from epithelial cells (46). However, the variability among lineage estimates also highlighted their dependence on experimental models, tracing strategies and marker definitions.

Subsequent evidence has increasingly supported resident mesenchymal populations as the predominant source of scar-forming myofibroblasts. Genetic fate tracing showed that labelled TECs remained within the tubular compartment after UUO or IRI rather than contributing substantially to interstitial α-SMA-positive myofibroblasts, supporting partial EMT or epithelial plasticity rather than complete lineage conversion (78). Collagen-I reporter studies similarly identified pericytes and perivascular fibroblasts as major collagen-producing populations, with only minor direct contributions from circulating fibrocytes (79). Consistently, integrated single-cell RNA sequencing, lineage tracing, ATAC-seq and spatial transcriptomics identified fibroblast and pericyte populations as predominant sources of matrix-producing myofibroblasts, whereas injured epithelial, endothelial and monocyte populations showed comparatively limited ECM production (80). These findings have shifted the emphasis from isolated mesenchymal-marker expression towards transcriptionally coherent and functionally matrix-producing cell states.

The contribution of macrophages remains more complex. Earlier bone marrow lineage-tracing and adoptive-transfer studies supported TGF-β/Smad3-dependent MMT, particularly in M2-like macrophages (68). However, recent single-cell and spatial analyses emphasize substantial macrophage heterogeneity rather than a uniform fibrogenic fate. Distinct infiltrating macrophage subsets occupy spatially restricted microenvironments and undergo dynamic redistribution during kidney injury and repair (81), while a proliferative M2-like macrophage population enriched after IRI displays profibrotic properties associated with platelet-derived THBS1 (82). These findings suggest that macrophage contributions to fibrosis depend strongly on cellular state and spatial context and may predominantly involve paracrine regulation of the fibrotic niche.

Recent high-resolution lineage tracing has further challenged complete EMT, EndMT and MMT as quantitatively important sources of renal myofibroblasts. Using lineage-specific and dual-recombinase systems capable of detecting stable and transient mesenchymal transitions, resident fibroblasts were identified as the predominant myofibroblast source, whereas TECs, endothelial cells and resident or recruited macrophages showed no substantial complete lineage conversion (83). Reversible partial mesenchymal transitions were detected in selected epithelial populations, with minimal partial EndMT and no detectable partial MMT. Thus, epithelial, endothelial and macrophage plasticity may remain functionally important through paracrine signaling and inflammatory–stromal crosstalk without representing major sources of scar-forming myofibroblasts.

Collectively, current evidence favors resident fibroblast/pericyte-like stromal populations as the predominant source of matrix-producing renal myofibroblasts, while epithelial, endothelial and immune cells contribute largely through context-dependent plasticity and intercellular signaling (80, 83). Importantly, single-cell transcriptomics cannot establish cellular ancestry, whereas genetic lineage tracing is influenced by promoter specificity, labelling efficiency and experimental context. Integration of lineage tracing with single-cell transcriptomics, chromatin accessibility profiling and spatial approaches therefore provides a more rigorous framework for distinguishing bona fide transdifferentiation from partial phenotypic transitions and defining the spatial organization of fibrogenic niches.

## 4 Anti-inflammatory and tissue-protective roles of TGF-β1

TGF-β1 is a pleiotropic cytokine that plays a central role in immune regulation, tissue repair, and fibrosis. While it is widely recognized for its profibrotic functions in chronic disease states, the anti-inflammatory and protective effects of TGF-β1, particularly during the early phases of tissue injury, are equally critical but often underappreciated. These immunomodulatory functions are context-dependent and intricately linked to the dynamic interactions between TGF-β1 and various immune cell subsets as well as resident parenchymal cells. To better understand this functional duality, this section focuses on the anti-inflammatory and reparative roles of TGF-β1 across three key cellular domains: innate immune cells, adaptive immune cells, and intrinsic renal cells (**Figure 2**; **Table 1**).

### 4.1 Macrophages

Macrophages play multifaceted roles in renal inflammation and fibrosis and are broadly categorized into pro-inflammatory M1 and profibrotic M2 phenotypes. TGF-β1 facilitates the polarization of M1 to M2 macrophages through both Smad-dependent and Smad-independent mechanisms, thereby contributing to its anti-inflammatory functions. For example, TGF-β1 secreted by mesenchymal stem cells (MSCs) promotes M2-like polarization of LPS-stimulated macrophages, suppresses inflammation, and enhances phagocytic capacity via the Akt/FOXO1 pathway (84). In both *in vivo* and *in vitro* settings, TGF-β1 also activates the NPY/Y1R axis to inhibit p38/NF-κB-mediated M1 activation while promoting the acquisition of a reparative M2 phenotype (85). Mechanistically, TGF-β1 upregulates the transcription factor SNAIL via Smad2/3 and PI3K/Akt signaling, thereby skewing macrophage polarization toward the M2-like state. Inhibition of the TGF-β/SNAIL axis restores the production of pro-inflammatory cytokines (86). Furthermore, TGF-β1 enables macrophages to adapt to chronic inflammatory environments through synergistic activation of Smad3 and signal transducer and activator of transcription 3 (STAT3) signaling (87). However, contrasting evidence suggests that while TGF-β robustly activates Smad3 in macrophages, it may not influence Smad-independent pathways in the same context (88). In murine IRI models, TGF-β1 has been shown to chemoattract monocyte-derived macrophages to the injured kidney. Nevertheless, this recruitment does not result in resolution of inflammation but instead leads to fibrosis (89). Importantly, macrophages modified *in vitro* with IL-10 and TGF-β1 markedly attenuate inflammation, structural damage, and functional decline in adriamycin-induced nephropathy (AN) models (90). The duration of TGF-β1 exposure appears to be a critical determinant of macrophage fate. Short-term stimulation (2 days) of inflammatory macrophages drives polarization toward the M2c subtype, which is primarily involved in anti-inflammatory and tissue repair processes. In contrast, prolonged exposure (5–7 days) promotes further transition to an M2a phenotype and ultimately to an αSMA^+^ myofibroblast-like state that contributes to collagen production and fibrosis (67, 91, 92).

### 4.2 Dendritic cells

Dendritic cells (DCs), together with macrophages, form a central component of the innate immune network in the kidney, playing a pivotal role in antigen presentation (93, 94). In human kidney biopsy specimens with interstitial fibrosis, the total number of DCs, including CD141hi and CD1c^+^ myeloid DC (mDC) subsets, was markedly elevated compared with fibrotic-free lesions or healthy renal tissue (95). Despite their immunological importance, the role of TGF-β1 in regulating renal DCs remains incompletely characterized. One study demonstrated that TGF-β1 activation via integrin αvβ8 enhances IL-1β–dependent fibroblast expression of the C-C motif chemokine ligand 20 (CCL20), which in turn promotes recruitment and migration of DCs through the C-C motif chemokine receptor 6 (CCR6) axis (96). Furthermore, TGF-β1 signaling, through both β-catenin and STAT3 pathways, suppresses key DC functions, including the differentiation, maturation, and endocytic capacity of human CD14^+^ monocyte-derived DCs. This inhibition limits the ability of mature DCs to stimulate allogeneic T cell proliferation and dampens their production of cytokines critical for Th1 and Th17 polarization, such as interferon-γ (IFN-γ), IL-12, IL-6, and IL-23 (97, 98). Neutralization of IL-10 and TGF-β receptors on DCs significantly enhances IFN-γ production and increases the cytotoxic potential of DC-activated effector T cells (99). Moreover, DC-derived TGF-β has been implicated as a key mediator of regulatory T cell (Treg) induction and immune tolerance (100). However, not all experimental evidence supports a renoprotective role for TGF-β1–modulated DCs. Although murine bone marrow–derived DCs can be rendered tolerogenic by IL-10 and TGF-β1 exposure, adoptive transfer of TGF-β1–preconditioned DCs failed to home to the renal cortex or ameliorate kidney injury in a mouse model of AN (101).

### 4.3 Neutrophils

Neutrophils are the most abundant leukocyte population in human peripheral blood and serve as the first line of cellular defense in the innate immune system. Through phagocytosis, degranulation, neutrophil extracellular traps (NETs) formation, and the release of immunomodulatory mediators, neutrophils actively participate in the pathophysiology of both acute kidney injury (AKI) and CKD (102, 103). In the UUO mouse model, gasdermin D (GSDMD)-dependent NETs formation triggers nuclear translocation of p65, which subsequently activates the TGF-β1/Smad signaling pathway and promotes macrophage expression of proinflammatory cytokines and α-SMA (69). A subset of Siglec-F–expressing neutrophils, derived from conventional intravascular neutrophils under the influence of TGF-β1 and GM-CSF, plays a critical role in establishing a profibrotic microenvironment in the kidney (104). Despite these observations, the immunoregulatory role of TGF-β1 in renal neutrophils remains poorly characterized. In oncology, however, TGF-β1 is well recognized for its role in polarizing neutrophils toward an N2 tumor-associated neutrophil (TAN) phenotype, thereby suppressing proinflammatory N1-like features such as tumor necrosis factor alpha (TNFα) expression. Inhibition of TGF-β1 enhances neutrophil chemoattractant expression and facilitates the recruitment of highly segmented N1 TANs that exhibit greater cytotoxicity toward tumor cells and produce higher levels of proinflammatory cytokines (105, 106). *In vitro* studies have shown that TGF-β1 activates canonical Smad3 signaling and non-canonical ERK1/2 pathways in human neutrophils, though it does not promote chemotaxis (107). Additionally, TGF-β1 reduces the adhesive capacity of TNFα-primed human neutrophils in adhesion assays (108). Collectively, these findings support an immunosuppressive role for TGF-β1 in neutrophil regulation, though its function in the kidney warrants further investigation.

### 4.4 Natural killer cells

Natural killer (NK) cells play a critical role in the pathogenesis of kidney disease (92, 109). In murine models of IRI and kidney transplantation, NK cells have been shown to be key mediators of inflammation and early immune injury (110, 111). In patients with CKD, renal biopsy specimens reveal an increased abundance of CD56bright NK cell subsets in the tubulointerstitial compartment, which contribute to renal fibrosis via the production of IFN-γ (112). TGF-β1 is a potent suppressor of NK cell function and exerts its regulatory effects through multiple mechanisms. At high concentrations, TGF-β1 modulates NK cell trafficking by upregulating the expression of C-X-C motif chemokine receptor 4 (CXCR4) and CXCR3 while downregulating CXCR1, thereby impairing NK cell recruitment to peripheral tissues (113). In primary human NK cells, TGF-β1 induces Smad3 phosphorylation and inhibits CD16-mediated IFN-γ production as well as antibody-dependent cellular cytotoxicity (114). Furthermore, TGF-β1 suppresses IFN-γ expression and the transcription factor T-BET (a key positive regulator of IFN-γ) through canonical Smad2, Smad3, and Smad4 signaling in isolated human NK cells (115, 116). In support of this mechanism, activated NK cells from Smad3-deficient mice produce significantly greater amounts of IFN-γ *in vivo*, highlighting the inhibitory role of Smad3 in TGF-β1–mediated NK cell regulation.

### 4.5 T lymphocytes

In CKD, the T cell compartment is dominated by helper CD4^+^ T cells (Th1, Th2, Th17), regulatory CD4^+^ T cells (Tregs), and cytotoxic CD8^+^ T cells. In proliferative glomerulonephritis such as IgA nephropathy and focal segmental necrotizing glomerulonephritis (FSNGN), elevated levels of Th1-, Th2-, and Th17-associated cytokines have been observed and are correlated with renal injury (117). Experimental crescentic glomerulonephritis is characterized by a nephritogenic Th1-driven immune response and T cell–mediated glomerular damage, with IL-12p40 functioning as a key Th1 cytokine subunit and IL-18 exerting local proinflammatory effects (118). In the UUO mouse model, inhibition of CD4^+^ T cell differentiation into Th2 cells has been proposed as a potential therapeutic approach to prevent renal fibrosis (119). TGF-β signaling broadly suppresses immune responses through multiple mechanisms. It promotes apoptosis of CD8^+^ T cells and downregulates cytotoxic mediators such as IFN-γ and TNFα (109, 120, 121). TGF-β1 also induces the expression of TIAF-1, an anti-apoptotic factor that protects renal parenchymal cells from apoptosis during allograft rejection (122). Tregs play a key role in immune modulation, peripheral tolerance, and antifibrotic defense. TGF-β1 is essential for the differentiation of naïve CD4^+^ T cells into CD4^+^Foxp3^+^ Tregs (123, 124). In contrast to well-functioning renal allografts, failed allografts due to antibody-mediated rejection show downregulation of regulatory markers including Foxp3, TGF-β1, RANTES, and CD20, indicating a loss of regulatory balance (125). TGF-β–induced CD4^+^Foxp3^+^ Tregs have been shown to attenuate acute antibody-mediated rejection *in vivo* and reduce proinflammatory M1 macrophage polarization (126).

In early DN, low-dose TGF-β1 administration not only avoids renal toxicity but also lowers blood glucose levels, attenuates kidney hypertrophy, reduces oxidative stress, maintains STAT3 activity, and promotes a favorable Treg/Th17 balance (127). In UUO models, inhibition of profibrotic β-catenin–TCF signaling redirects β-catenin interaction toward FOXO1, a transcription factor that enhances TGF-β–driven inducible Treg (iTreg) differentiation, thereby promoting the anti-inflammatory rather than fibrotic arm of TGF-β signaling (128). Moreover, mesenchymal stromal cells (MSCs) overexpressing TGF-β1 have shown therapeutic potential in promoting local renal Treg generation following IRI (129). In autoimmune nephritis models, targeted delivery of IL-2 and TGF-β via PLGA nanoparticles to T cells significantly expands both CD4^+^ and CD8^+^ Tregs *in vivo*, suppressing the pathogenic renal immune responses associated with systemic lupus erythematosus (SLE) (130).

### 4.6 B lymphocytes

B cells are typically considered proinflammatory lymphocytes that contribute to adaptive immune responses primarily through antibody production. In the context of kidney transplantation, although peripheral B cell repertoire diversity is generally reduced, immune repertoire sequencing of B cells has been used to assess the risk of graft rejection both before and after transplantation (131). The presence of B cells and immunoglobulin transcripts is a hallmark of chronic inflammation in late-stage allograft rejection (132). C-X-C motif chemokine ligand 13 (CXCL13), also known as B lymphocyte chemoattractant, mediates B cell recruitment to lymphoid follicles and has recently emerged as a biomarker of acute renal allograft rejection (133). Experimental strategies that deplete or suppress B cells have demonstrated improved renal graft survival (134, 135). In mouse models of bilateral IRI, early immune responses are linked to the emergence of B cell–related signatures associated with late-stage renal dysfunction and fibrosis (136). In unilateral IRI, single-cell RNA sequencing revealed an increase in CXCR5-expressing CD22^+^ B cells infiltrating the ischemic kidney (133). Adoptive transfer of B cells into B cell–deficient mice results in reduced tubular cell proliferation but increased tubular atrophy (137). In obesity-related kidney disease, CD3^−^CD19^+^ B cells exacerbate tubular injury by expressing miR-3960, which targets the nuclear gene TRMT5, impairing mitochondrial respiration in TECs (138). In nephrotic syndrome, activated B cells produce proinflammatory cytokines via the IL-4/STAT6 axis, contributing to podocyte damage and proteinuria (139).

To date, the role of TGF-β1 in regulating B cell responses in kidney disease remains poorly understood. TGF-β1 has been shown to suppress the migration of activated B cells via the SDF-1–CXCR4 axis (140). Through ligation of toll-like receptors (TLR), including TLR4, TLR7, and TLR9, B cells can induce the expression of membrane-bound GARP–TGF-β complexes, which suppress B cell proliferation and promote IgA class-switch recombination (141). In lupus nephritis models, natural CD4^+^Foxp3^+^ regulatory T cells (nTregs) inhibit B cell responses via cytotoxic mechanisms involving granzyme A, granzyme B, and perforin. In contrast, inducible Tregs (iTregs), generated *in vitro* with TGF-β, suppress B cell activation and proliferation through direct engagement of TGF-β signaling (142, 143). Moreover, a subset of regulatory B cells (Bregs) contributes to immune suppression through the production of immunomodulatory cytokines, including IL-10, TGF-β1, and IL-35 (144).

### 4.7 Intrinsic renal cells

TGF-β1 exerts protective and reparative effects following kidney injury; however, sustained or excessive TGF-β1 signaling ultimately leads to maladaptive fibrotic remodeling. One study reported that TGF-β induces the release of soluble MHC class I–related chain A (MICA) from HKII proximal tubular cells, leading to downregulation of NKG2D on NK cells and thereby limiting NK cell–mediated cytotoxicity against proximal TECs in renal IRI (145). TGF-β1 has also been shown to suppress the expression of MHC class II and B7–1 on TECs, thereby impairing their antigen-presenting capacity *in vitro (*146). Intrinsic TGF-β signaling attenuates mitochondrial injury and inflammation in proximal tubular cells during CKD (147). Furthermore, TGF-β may negatively regulate macrophage-mediated profibrotic activation of mesangial cells by enhancing ECM degradation and reducing matrix synthesis (148).

*In vitro* disruption of TGFBR2 in renal fibroblasts or TECs effectively blocks TGF-β1–induced Smad signaling and fibrotic gene expression. However, this also impairs the anti-inflammatory effects of TGF-β1, including its ability to suppress IL-1β–induced NF-κB activation and proinflammatory cytokine production (149). These findings highlight a central paradox of TGF-β1 signaling in the kidney: while it promotes fibrosis under chronic stimulation, complete inhibition of TGF-β1 expression or receptor binding can abrogate its essential tissue-protective and anti-inflammatory functions during acute injury.

## 5 Cellular sources and spatiotemporal regulation of TGF-β1

The biological effects of TGF-β1 in CKD are determined not only by its abundance but also by its cellular origin, spatial distribution, duration of activation, and the cellular state of the responding tissue. Kidney injury dynamically alters both TGF-β1-producing and TGF-β1-responsive cell populations, generating localized signaling niches that may support immune regulation and tissue repair or, when sustained, promote maladaptive remodeling and fibrosis. This spatiotemporal heterogeneity provides an important framework for reconciling the apparently paradoxical functions of TGF-β1 during CKD progression (**Figure 3**).

**Figure 3:**
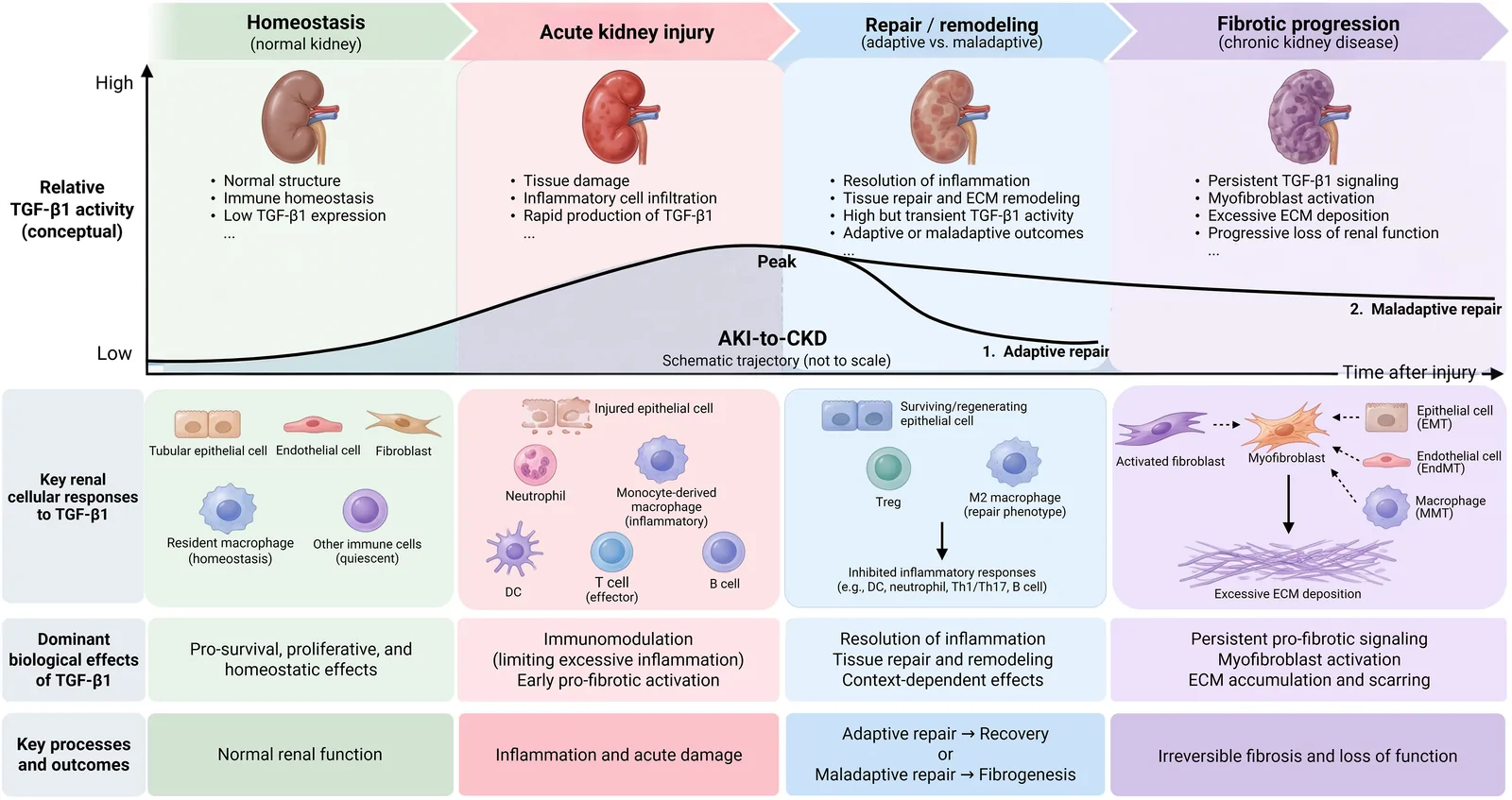
Conceptual spatiotemporal dynamics and context-dependent functions of TGF-β1 during the AKI-to-CKD transition. The schematic illustrates the proposed temporal changes in TGF-β1 activity and the corresponding cellular and biological responses across four stages of kidney injury: homeostasis, acute kidney injury (AKI), repair/remodeling, and fibrotic progression. Under physiological conditions, relatively low basal TGF-β1 activity contributes to renal homeostasis, cell survival, and immune regulation. Following AKI, TGF-β1 activity increases in parallel with tissue injury and inflammatory-cell infiltration. During the subsequent repair/remodeling phase, transiently elevated TGF-β1 signaling may contribute to resolution of inflammation, tissue repair, extracellular matrix (ECM) remodeling, regulatory T-cell responses, and reparative macrophage phenotypes. The outcome of this phase is context dependent: appropriate resolution of TGF-β1 signaling may facilitate adaptive repair and functional recovery, whereas persistent or dysregulated signaling may promote maladaptive repair and progression toward fibrosis. In established CKD, sustained TGF-β1 activity above physiological baseline promotes fibroblast and myofibroblast activation, excessive ECM deposition, tissue scarring, and progressive loss of renal function. Resident fibroblasts and pericyte-like stromal cells represent major sources of matrix-producing myofibroblasts, whereas epithelial, endothelial, and macrophage populations may contribute predominantly through partial phenotypic transitions and paracrine profibrotic signaling; their direct contributions through complete epithelial-to-mesenchymal transition (EMT), endothelial-to-mesenchymal transition (EndMT), or macrophage-to-myofibroblast transition (MMT) remain limited and/or debated. The TGF-β1 trajectory shown here is conceptual and not to scale and is intended to illustrate a proposed temporal shift in the predominant biological functions of TGF-β1 rather than a quantitatively measured kinetic profile. Created in BioRender.

### 5.1 Cellular sources of TGF-β1 in CKD

TGF-β1 is produced by multiple renal parenchymal, stromal, and immune cell populations, and the dominant cellular sources vary with the etiology and stage of kidney injury. TECs can increase TGF-β1 production in response to hypoxia, metabolic stress, proteinuria, and inflammatory injury, thereby influencing neighboring fibroblasts and immune cells. Glomerular cells, including podocytes, mesangial cells, and endothelial cells, also contribute to local TGF-β1 signaling, particularly in glomerular and metabolic kidney diseases. In the interstitium, activated fibroblasts and pericyte-derived stromal populations participate in reciprocal profibrotic signaling and constitute the major precursors of scar-forming myofibroblasts identified by integrated single-cell, lineage-tracing, and spatial analyses (80).

Immune cells provide an additional and highly dynamic source of TGF-β1. Following injury, recruited monocyte-derived macrophages can produce abundant TGF-β1 and other profibrotic mediators, thereby promoting fibroblast activation and ECM deposition. In contrast, tissue-resident macrophages have distinct homeostatic and reparative functions, emphasizing that macrophage-derived TGF-β1 should not be regarded as uniformly profibrotic. T cells, Tregs, DCs, and other infiltrating leukocytes may further contribute to the local TGF-β1 milieu. Thus, rather than arising from a single dominant cellular source, renal TGF-β1 represents a multicellular signaling network whose functional consequences depend on the identity and activation state of both producing and responding cells.

### 5.2 Spatial and temporal heterogeneity of TGF-β1 signaling in CKD

The transition from AKI to CKD illustrates the temporal complexity of TGF-β1 signaling. TGF-β1 is rapidly induced after renal injury and can initially participate in immunoregulation, cell survival, and tissue repair. However, persistent pathway activation during maladaptive repair favors fibroblast activation, ECM accumulation, and progressive loss of renal architecture. Accordingly, the biological consequences of TGF-β1 are better represented as a dynamic, context-dependent trajectory rather than a monotonic increase in expression.

Recent spatially resolved transcriptomic studies have provided direct evidence that intrarenal TGF-β1 activity is regionally organized rather than uniformly distributed. In IRI, spatial transcriptomics demonstrated increased TGF-β1 expression within macrophage-enriched regions, accompanied by spatial enrichment of macrophage and fibrotic signatures. A proliferative, M2-like macrophage subset was further identified within these injury-associated niches, linking local immune-cell heterogeneity to profibrotic TGF-β1 activity (82). At a broader tissue level, spatial and single-cell multi-omic profiling of human healthy, diabetic and hypertensive kidneys has identified distinct glomerular, tubular, immune and fibrotic microenvironments, demonstrating substantial regional reorganization of cellular states and signaling networks during CKD (150). Spatial transcriptomic analysis of experimental CKD similarly revealed disruption of normal cortico-medullary organization and demonstrated that the consequences of TGF-β signaling differ across proximal tubular and surrounding interstitial compartments. Notably, proximal tubular deletion of TGF-β2 aggravated mitochondrial injury, inflammation and renal remodeling, highlighting that spatially restricted TGF-β signaling may exert protective as well as profibrotic effects depending on the responding cell population. Together, these observations indicate that the biological consequences of intrarenal TGF-β1 are determined not only by its overall abundance but also by the spatial organization of ligand-producing cells, responding cell populations and the surrounding fibrotic microenvironment.

### 5.3 Epigenetic and non-coding RNA regulation of TGF-β1 signaling

The persistence of TGF-β1 signaling after the initiating injury has resolved is partly maintained by epigenetic and post-transcriptional regulatory mechanisms. TGF-β/Smad signaling can reshape chromatin accessibility and transcriptional programs in injured epithelial and stromal cells, thereby stabilizing profibrotic cell states. Conversely, chromatin modifications and transcriptional cofactors can alter the accessibility of Smad-responsive genes, providing a mechanism through which previous injury may influence subsequent TGF-β responses.

Non-coding RNAs constitute a particularly well-characterized regulatory layer. TGF-β1 induces miR-21 through Smad3, and increased miR-21 promotes renal fibrosis, whereas Smad3 deficiency prevents its induction (151). In DN, miR-21 further amplifies TGF-β1 signaling through suppression of Smad7 and PTEN, thereby enhancing Smad3- and PI3K/AKT-dependent profibrotic responses (152). Long non-coding RNAs similarly participate in reciprocal regulatory circuits with TGF-β/Smad signaling. The Smad3-dependent lncRNA Erbb4-IR suppresses Smad7 and promotes renal fibrosis (153), whereas the kidney-enriched lnc-TSI interacts with Smad3 and inhibits its activation, thereby restraining fibrogenesis (154).

These findings indicate that epigenetic and non-coding RNA networks can function as molecular “memory” mechanisms that reinforce or restrain TGF-β1 signaling after kidney injury. Such regulatory layers may therefore contribute to the transition from reversible repair to persistent fibrosis and provide opportunities to modulate pathological TGF-β signaling without abolishing its physiological functions.

## 6 Crosstalk between TGF-β signaling and other pathways

An increasing body of evidence indicates that the fibrogenic activity of TGF-β1 does not operate in isolation but is tightly regulated through extensive crosstalk with other signaling networks. These interactions shape cellular responses to injury and influence the balance between fibrotic resolution and progression. Elucidating the interplay between TGF-β1 and other pathways is critical to understanding the complexity of fibrosis regulation and may reveal novel therapeutic targets for intervention (**Figure 4**).

**Figure 4:**
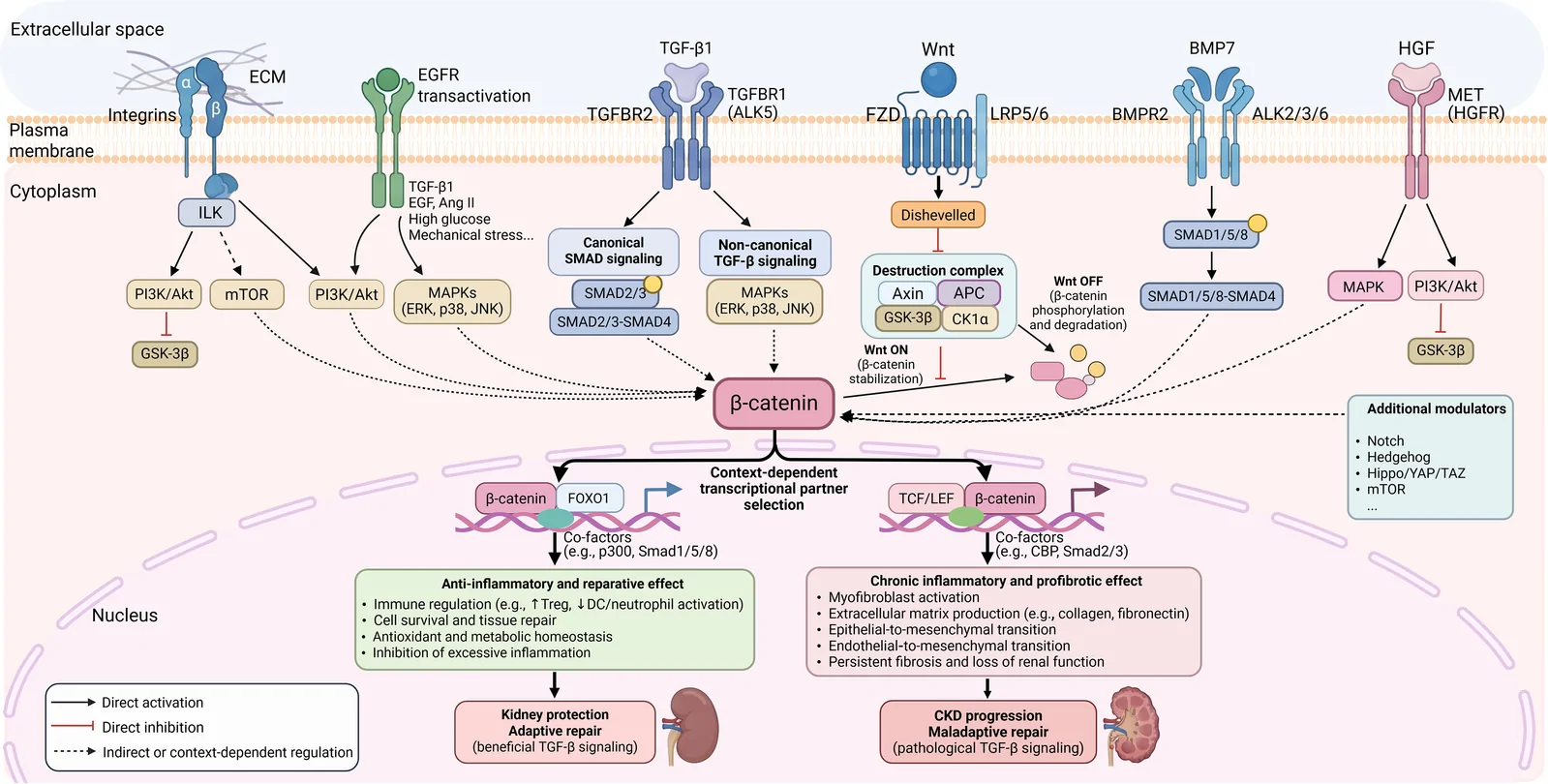
Crosstalk between TGF-β1 signaling and β-catenin-centered regulatory networks determines context-dependent renal responses. TGF-β1 signaling interacts with multiple signaling pathways that converge on β-catenin and collectively shape its context-dependent transcriptional output. Canonical TGF-β1 signaling is initiated through TGFBR2 and TGFBR1 (ALK5), leading to Smad2/3 phosphorylation and formation of Smad2/3–Smad4 complexes, whereas non-canonical TGF-β signaling activates pathways including MAPKs. Integrin/integrin-linked kinase (ILK), epidermal growth factor receptor (EGFR), Wnt, bone morphogenetic protein 7 (BMP7), and hepatocyte growth factor (HGF)/MET signaling further modulate β-catenin activity through PI3K–Akt, mTOR, MAPK, GSK3β, Smad, and related regulatory mechanisms. In canonical Wnt signaling, Wnt binding to Frizzled (FZD) and LRP5/6 inhibits the Axin–APC–GSK3β–CK1α destruction complex, thereby stabilizing β-catenin and facilitating its nuclear accumulation. In the nucleus, the biological consequences of β-catenin signaling depend partly on context-dependent transcriptional partner selection. Association of β-catenin with FOXO1 and associated cofactors may support anti-inflammatory, stress-adaptive, and reparative programs, whereas β-catenin–TCF/LEF-associated transcription can promote chronic inflammatory and profibrotic programs, including myofibroblast activation, extracellular matrix production, and maladaptive tissue remodeling. Additional pathways, including Notch, Hedgehog, Hippo/YAP/TAZ, and mTOR signaling, further influence this regulatory network. Importantly, β-catenin partner selection is presented as a context-dependent regulatory framework rather than an absolute binary switch, with the ultimate biological outcome determined by cell type, injury state, signaling intensity, and the surrounding molecular environment. Solid black arrows indicate direct activation, red blunt-ended lines indicate direct inhibition, and dashed arrows indicate indirect or context-dependent regulation. Created in BioRender.

### 6.1 Wnt/β-catenin signaling

Wnt signaling is a pivotal pathway that regulates tissue differentiation and proliferation during embryogenesis and maintains tissue homeostasis in adulthood (155). Wnt pathways are broadly classified into canonical (β-catenin–dependent) and non-canonical (β-catenin–independent) branches. The canonical Wnt/β-catenin pathway has been closely linked to the development of fibrosis in kidney disease, whereas non-canonical Wnt signaling is primarily involved in regulating cell polarity and migration (156, 157). In the absence of Wnt activation, cytoplasmic β-catenin is targeted for degradation by a destruction complex composed of GSK3β, adenomatous polyposis coli (APC), casein kinase 1 (CK1), and axin. This complex phosphorylates and ubiquitinates β-catenin, promoting its proteasomal degradation (158). Upon Wnt ligand binding to Frizzled (FZD) receptors and co-receptors LRP5/6, Dishevelled (DVL) proteins are recruited to the membrane. DVL binds to axin and GSK3β, thereby disrupting the formation of the destruction complex and preventing β-catenin degradation (155, 159). Stabilized β-catenin subsequently translocates to the nucleus, where it associates with TCF and lymphoid enhancer factor (LEF) to regulate the transcription of profibrotic genes, including α-SMA, collagens, fibronectin, Snail, components of the RAS, and TGF-β1 itself (159, 160).

Wnt/β-catenin signaling represents one of the key crosstalk pathways with TGF-β1. On one hand, activation of Wnt/β-catenin in the UUO mouse model promotes M2 macrophage polarization and synergizes with TGF-β1 to exacerbate renal fibrosis and stimulate further TGF-β1 production (160, 161). Notably, this signaling does not alter Smad expression or phosphorylation levels (162). On the other hand, TGF-β1 has been shown to induce Wnt/β-catenin signaling and increase β-catenin expression in fibrogenic stroma (128, 163, 164). Within the TGF-β1/Smad3 signaling axis, the formation of Smad3–β-catenin complexes is required to achieve sufficient DNA-binding affinity for the transcription of specific target genes (21, 128).

### 6.2 MAPK signaling

The MAPK family comprises three well-characterized members: ERK, JNK, and p38 MAPK (165). Activation of these MAPK pathways has been consistently observed across a range of kidney diseases, where they contribute to the progression of renal fibrosis (166, 167). In the non-canonical TGF-β signaling cascade, MAPK pathways function as direct mediators of transcriptional responses (6, 168). One study demonstrated that TGF-β binding to TGFBR1 leads to phosphorylation of serine and tyrosine residues on SHC-transforming protein 1 (ShcA), which in turn facilitates the recruitment of Grb2/Sos and activation of the classical ERK cascade commonly associated with receptor tyrosine kinases (169). TGF-β can also activate ERK through the modulation of secreted modular calcium-binding protein 2 (SMOC2) and proteinase-activated receptor 2 (PAR2) (170, 171). In addition, TGF-β induces K63-linked ubiquitination of TRAF6, promoting its interaction with TAK1 and subsequent activation of both JNK and p38 MAPK pathways (172).

Numerous studies have shown that TGF-β1–induced activation of MAPK pathways drives myofibroblast activation and differentiation in the kidney, thereby promoting fibrotic progression (173, 174). Moreover, MAPK signaling can modulate the TGF-β/Smad pathway. Several studies have shown that targeting MAPKs can alter TGF-β1 expression, collagen synthesis, and myofibroblast activation—representing a promising antifibrotic strategy for CKD (166, 175, 176). Both hyperglycemia and angiotensin II have been reported to stimulate the expression of TGF-β1 in various renal cell types through MAPK activation, thereby accelerating fibrotic processes (177–179). Interestingly, angiotensin II can also activate Smad proteins via a MAPK-dependent mechanism that is independent of TGF-β1, both *in vivo* and *in vitro*, thus contributing to renal fibrosis (180, 181). Furthermore, renin itself has been shown to rapidly activate ERK signaling in mesangial cells via receptor-mediated mechanisms, leading to intracellular upregulation of TGF-β1 independently of angiotensin II, and promoting fibrogenesis (182).

### 6.3 integrin–ILK signaling

ILK has emerged as a profibrotic mediator in various kidney diseases (183, 184). On one hand, ILK can promote the transcription of TGF-β1 through multiple mechanisms (185, 186). On the other hand, ILK expression is also regulated by TGF-β1. In TECs, TGF-β1 induces ILK expression in a time- and dose-dependent manner via Smad signaling.

Forced overexpression of ILK in human proximal TECs suppresses E-cadherin expression while inducing fibronectin expression and its extracellular assembly (187). Inhibition of ILK activity abrogates TGF-β1–induced phosphorylation of Akt and GSK-3β, thereby stabilizing β-catenin and preserving canonical Wnt/β-catenin signaling (188, 189).

Moreover, TGF-β1 promotes fibrosis through ILK upregulation via integrin α3–mediated signaling, involving phosphorylation of β-catenin at Y654 and Smad2 activation, which contributes to E-cadherin downregulation (190, 191). A recent study further demonstrated that TGF-β1 induces the expression of CTGF, collagen, integrin α/β subunits, and ILK through the myocardin-related transcription factor (MRTF)–serum response factor (SRF) axis (192).

### 6.4 mTOR signaling

The mechanistic target of rapamycin (mTOR) signaling pathway is activated in macrophages and myofibroblasts during renal fibrosis (65, 193, 194). Activation of the Rictor/mTORC2 complex mediates TGF-β1–induced fibroblast activation and M2 macrophage polarization, thereby contributing to the progression of kidney fibrosis (195, 196). TGF-β1 has also been shown to promote PMT by enhancing glycolytic flux through the PI3K–Akt–mTOR axis, upregulating HK expression (63). Crosstalk between TGF-β1 and mTOR signaling is further reinforced by shared upstream mediators such as PI3K/Akt, which is also activated by ILK. Importantly, GSK3β, a downstream substrate of Akt, serves as a convergence point for mTOR signaling and Wnt/β-catenin pathway activation (197–199).

Autophagy represents another context-dependent component of the TGF-β signaling network (200, 201). Notably, its effects on renal fibrogenesis appear to depend on the duration and stage of kidney injury. Transient autophagic activation may exert an antifibrotic effect by promoting the degradation of mature TGF-β1, thereby limiting TGF-β-dependent fibrotic signaling (202). In contrast, persistent tubular autophagy during maladaptive repair after AKI can promote renal fibrosis. Sustained autophagy activates EGR1/2 through MAPK/ERK signaling in TECs, inducing FGF2 production and a profibrotic secretory phenotype that stimulates interstitial fibroblast activation (166, 203). Thus, the temporal switch from transient to sustained autophagy provides another example of how the biological consequences of TGF-β-associated signaling networks are determined by cellular context and disease stage.

### 6.5 EGFR signaling

Epidermal growth factor receptor (EGFR), a member of the receptor tyrosine kinase family, has been implicated in the pathogenesis of renal fibrosis by regulating cellular processes such as proliferation, differentiation, and migration (204). On one hand, activation of EGFR is required for TGF-β1–induced transcription of fibrotic genes in renal cells (205). TGF-β1, angiotensin II, and epidermal growth factor have all been shown to activate EGFR and promote EMT in TECs via the PI3K/Akt signaling pathway (206). Angiotensin II further induces tubular epithelial hypertrophy through coordinated activation of EGFR and TGF-β1/Smad3 signaling (207, 208). TGF-β1 can also transactivate EGFR via Src kinase, leading to downstream activation of ERK and STAT3 pathways and contributing to Smad3-mediated profibrotic responses in mesangial cells, fibroblasts, and TECs (209, 210). Hyperglycemia has likewise been shown to induce EMT via the EGFR/ERK pathway (211).

On the other hand, EGFR also plays a role in the regulation of TGF-β1 transcription. High glucose induces phosphorylation of Akt at Ser473 in a protein kinase C-β (PKC-β)–dependent manner, leading to upregulation of TGF-β1 through the AP-1 transcription factor (212, 213). In a mouse model of DN, human heparin-binding EGF-like growth factor (hHB-EGF) activates EGFR tyrosine kinase and subsequently triggers ERK and AKT pathways, as well as the profibrotic TGF-β1/Smad signaling cascade (210). Notably, EGFR knockout significantly reduces TGF-β1 expression, Smad2/3 phosphorylation, and glomerular fibronectin deposition in DN mice (214).

### 6.6 Notch signaling

Notch signaling, an evolutionarily conserved pathway, plays a crucial role in renal fibrogenesis and engages in extensive crosstalk with TGF-β signaling (215). TGF-β1 has been shown to induce the expression of Jagged1 and Notch receptors in TECs and podocytes via a Smad3/ERK-dependent mechanism, thereby promoting the transcription of mesenchymal markers such asα-SMA, vimentin, and Snail1 (216, 217). In renal fibroblasts, TGF-β1 activates fibrogenic gene expression through the JNK/Notch2 axis (218).

Notably, Notch signaling also contributes to mesenchymal transformation of pericytes, endothelial cells, and mesangial cells, thereby facilitating fibrotic progression (219, 220). Indeed, Jagged–Notch signaling has been proposed to be essential for TGF-β1–driven fibrotic gene expression (221). Moreover, *de novo* expression of Notch1 in podocytes leads to increased TGF-β1 transcript levels both *in vitro* and *in vivo*, potentially accounting for the upregulation of TGF-β1 observed in a variety of glomerular diseases (222). Interestingly, in the UUO mouse model, Notch activation has been implicated in monocyte recruitment and the transdifferentiation of macrophages into myofibroblast-like cells (72), suggesting the existence of a positive feedback loop between TGF-β1 and Notch signaling pathways.

### 6.7 BMP7 signaling

BMP7, a member of the TGF-β superfamily, exerts anti-fibrotic effects that are functionally antagonistic to those of TGF-β1. BMP7 signals through a complex of type I receptors: activin receptor type 1 (ACVR1; also known as ALK2), BMP receptor type 1A (BMPR1A; ALK3), and BMPR1B (ALK6); and type II receptors: BMPR2, ACVR2A, and ACVR2B. Ligand binding initially occurs at the type II receptor, which subsequently recruits and phosphorylates the type I receptor to form a stable heterotetrameric complex. Like TGF-β1, BMP7 signaling involves both canonical Smad-dependent and non-canonical Smad-independent pathways. In the canonical pathway, BMP7 activates Smad1/5/8, which then forms a phosphorylated complex with Smad4 that translocates to the nucleus to regulate the transcription of target genes. BMP7 also signals through MAPK, including ERK and p38, as well as the Akt pathway (223).

Accumulating evidence indicates that BMP7 expression is reduced during renal fibrogenesis (224–226). BMP7 has been shown to stimulate proliferation and cadherin-11 expression in metanephric mesenchymal cell lines in a dose-dependent manner via ERK and p38 activation (224). In a rat model of uremia, exogenous BMP7 suppresses the expression of pro-fibrotic genes in the kidney (227). In models of DN, BMP7 restores SnoN protein levels via the Smad1/5 axis, thereby partially reversing epithelial–mesenchymal transition (225, 228). Moreover, administration of recombinant human BMP7 (rhBMP7) in UUO mice prevented fibrosis through activation of Smad1/5/8 and inhibition of Akt signaling (229). Given the mechanistic parallels between BMP7 and TGF-β1, the ability of BMP7 to functionally antagonize TGF-β1 has substantial preclinical therapeutic potential (230, 231). However, in a murine myocardial infarction model, BMP signaling activated Smad5, ERK, and AKT, rather than Smad1, thereby promoting reparative responses in cardiomyocytes (232). These findings highlight the context-dependent nature of BMP7 signaling and underscore the need to further elucidate its role in balancing anti-fibrotic and reparative processes.

### 6.8 Context-dependent consequences of TGF-β signaling crosstalk

The biological consequences of TGF-β signaling in the kidney cannot be attributed to the activation of a single linear pathway. Rather, TGF-β functions within an interconnected signaling network in which Wnt/β-catenin, MAPK, integrin–ILK, PI3K–Akt–mTOR, EGFR, Notch and BMP7 pathways dynamically modify the magnitude, duration and transcriptional output of TGF-β signaling. Several of these pathways establish positive feedback loops that reinforce profibrotic responses. For example, MAPK and EGFR signaling can enhance TGF-β1 expression and Smad-dependent transcription, whereas integrin–ILK and PI3K/Akt signaling converge on GSK-3β and thereby influence β-catenin stability. Wnt signaling further promotes β-catenin accumulation by suppressing the β-catenin destruction complex. Together, these interactions can amplify fibroblast activation, mesenchymal transition programs and extracellular matrix production, particularly during persistent renal injury.

Importantly, however, signaling crosstalk does not invariably enhance fibrosis. The same signaling nodes can produce distinct or even opposing biological effects depending on the cell type, injury stage and accompanying extracellular signals. BMP7 provides a prominent example by activating Smad1/5/8 and counterbalancing TGF-β1/Smad2/3-mediated profibrotic responses, thereby promoting epithelial preservation and adaptive repair in several experimental settings. Similarly, pathways such as MAPK, PI3K/Akt and EGFR participate not only in fibrogenesis but also in cell survival, proliferation and tissue regeneration. Consequently, inhibition of an individual signaling component may suppress pathological fibrosis while simultaneously interfering with physiological repair. These observations indicate that the functional consequence of TGF-β crosstalk is determined not simply by whether a pathway is activated, but by the cellular state, temporal pattern of activation and combination of signaling inputs.

A major point of convergence within this network is β-catenin, whose stability and transcriptional activity are influenced by Wnt, integrin–ILK, PI3K/Akt, EGFR and other signaling pathways. β-catenin can cooperate with Smad complexes and different nuclear transcriptional partners, allowing similar upstream TGF-β signals to generate divergent transcriptional programs. In a profibrotic context, sustained β-catenin signaling and its association with TCF/LEF can reinforce transcription of genes involved in fibroblast activation and extracellular matrix remodeling. By contrast, alternative transcriptional interactions, including β-catenin–FOXO1-associated programs, may favor anti-inflammatory and adaptive responses. Thus, pathway crosstalk should be viewed as a mechanism that reprograms the output of TGF-β signaling rather than simply strengthening or weakening its activity. This context dependence provides a mechanistic explanation for the paradoxical functions of TGF-β1 in CKD and suggests that selectively redirecting downstream signaling outputs may offer a more rational therapeutic strategy than global TGF-β inhibition.

## 7 Clinical translation of TGF-β targeted therapies in CKD

Despite compelling experimental evidence identifying TGF-β1 as a central mediator of renal fibrosis, translation of TGF-β-targeted therapies into clinical benefit has remained challenging. Our previous systematic review identified six published clinical trial reports and 14 registered trials evaluating anti-TGF-β-related interventions in CKD and found that, although several approaches showed encouraging preclinical or early clinical signals, randomized trials of direct TGF-β neutralization failed to demonstrate consistent improvements in renal outcomes (233). These discrepancies highlight an important distinction between the pathogenic importance of TGF-β signaling and its suitability for indiscriminate therapeutic inhibition. The clinical experience to date therefore provides critical lessons for the development of more selective and context-dependent strategies (**Table 2**).

**Table 2:** Clinical trials and translational barriers of TGF-β-targeted therapies in CKD.

Drugs	Therapeutic mechanism	Clinical context	Stage	Main findings	Major translational lesson	References
Pirfenidone	Small-molecule antifibrotic agent; indirectly suppresses TGF-β-associated fibrotic signaling	FSGS; diabetic nephropathy; CKD	Pilot and Phase I–II studies	Slower eGFR decline or improved eGFR was reported in selected studies, but efficacy was inconsistent and higher doses were less well tolerated	Partial/pleiotropic modulation may differ fundamentally from direct TGF-β neutralization	(234–236)
Fresolimumab (GC1008)	Pan–TGF-β neutralizing monoclonal antibody	Treatment-resistant/steroid-resistant FSGS	Phase I–II	Phase I established initial feasibility, whereas Phase II did not significantly improve the prespecified proteinuria endpoint	Systemic pan–TGF-β neutralization may lack adequate biological and therapeutic selectivity	(16, 17)
LY2382770	TGF-β1-selective monoclonal antibody	Diabetic nephropathy	Phase II	Did not improve serum creatinine, eGFR or proteinuria and was terminated early for futility	Ligand isoform selectivity alone may be insufficient to overcome the limitations of systemic TGF-β blockade	(15)
Bexotegrast (PLN-74809)	Dual αvβ6/αvβ1 integrin inhibitor; inhibits latent TGF-β activation	Fibrotic diseases; kidney-relevant experimental evidence	Clinical development in fibrotic diseases	Supports selective targeting of disease-associated latent TGF-β activation	Upstream activation-selective inhibition may provide greater biological precision than systemic ligand neutralization	(291, 308)
Sparsentan	Dual endothelin type A and angiotensin II type 1 receptor antagonist	Proteinuric CKD	Clinically established/advanced	Reduces proteinuria and modifies profibrotic signaling networks	Effective antifibrotic treatment does not necessarily require direct TGF-β inhibition	(309, 310)
Finerenone	Non-steroidal mineralocorticoid receptor antagonist	CKD associated with type 1/2 diabetes	Phase III/clinical use	Reduces renal and cardiovascular events and modulates inflammation/fibrosis-associated pathways	Indirect targeting of interconnected fibrotic networks may avoid liabilities of pan–TGF-β blockade	(292, 311, 312)

### 7.1 Lessons from clinical trials of TGF-β targeted therapies

Pirfenidone represents the most extensively investigated small-molecule antifibrotic agent with TGF-β-modulatory activity in CKD. In an early open-label study of 21 patients with focal segmental glomerulosclerosis (FSGS), pirfenidone treatment was associated with an approximately 25% reduction in the rate of eGFR decline among patients completing treatment, although proteinuria and blood pressure were not significantly improved (234). A subsequent randomized trial involving 77 patients with DN reported improvement in eGFR after one year in the 1,200-mg/day group compared with placebo, whereas the 2,400-mg/day group showed a higher discontinuation rate and no significant benefit, suggesting that increased systemic exposure does not necessarily translate into greater renal efficacy (235). More recently, a Phase I bridging study in individuals with CKD stages G2–G3a found that a single 400-mg dose of pirfenidone had broadly comparable pharmacokinetic characteristics across renal-function groups and was not associated with severe adverse events (236).

A larger randomized Phase II study, TOP-CKD (NCT04258397), was subsequently designed to evaluate pirfenidone in approximately 200 patients with CKD at risk of progression. Notably, this study incorporated diffusion-weighted renal magnetic resonance imaging and urinary markers of tubulointerstitial fibrosis, including α1-microglobulin, procollagen III N-terminal peptide and MCP-1, providing a potentially informative biomarker-based framework for assessing antifibrotic activity. However, as of September 2026, no study results have been posted on ClinicalTrials.gov, and the registry status remains unknown following its last verification in April 2022. Thus, its efficacy cannot currently be evaluated.

More direct neutralization of TGF-β has produced less encouraging results. Fresolimumab (GC1008), a monoclonal antibody that neutralizes all three TGF-β isoforms, was initially evaluated in a Phase I dose-escalation study involving 16 patients with treatment-resistant primary FSGS. Single-dose administration was generally tolerated, providing initial evidence that systemic TGF-β neutralization was clinically feasible (16). However, in a subsequent randomized Phase II study of steroid-resistant primary FSGS, only 36 patients were enrolled before premature study closure. Neither 1 nor 4 mg/kg fresolimumab produced a significant improvement in the prespecified proteinuria remission endpoint, although interpretation was limited by the small sample size and early termination (17).

Similarly, LY2382770, a humanized monoclonal antibody selectively targeting TGF-β1, failed to translate strong experimental rationale into clinical efficacy. In a randomized Phase II trial of patients with moderate-to-advanced DN receiving background renin–angiotensin system blockade, monthly administration of LY2382770 at 2, 10 or 50 mg did not improve serum creatinine, eGFR or proteinuria compared with placebo, and the study was terminated early for futility (15). Importantly, the absence of efficacy with both pan-TGF-β and TGF-β1-selective antibodies suggests that simply increasing ligand specificity may be insufficient to overcome the fundamental biological and pharmacological limitations of sustained systemic TGF-β blockade.

Collectively, clinical experience has therefore produced a consistent message: the strong association between TGF-β activity and renal fibrosis does not imply that global inhibition of TGF-β will necessarily improve CKD outcomes. The modest signals observed with pirfenidone, contrasted with the negative antibody trials, further suggest that partial or pleiotropic modulation of fibrotic networks may differ fundamentally from complete ligand neutralization.

### 7.2 Molecular and clinical determinants of therapeutic failure

Several factors may explain the substantial gap between preclinical efficacy and clinical outcomes. Foremost is the pleiotropic and context-dependent biology of TGF-β1. In addition to driving fibroblast activation and extracellular matrix deposition, TGF-β1 regulates immune tolerance, limits excessive inflammatory responses and participates in epithelial homeostasis and tissue repair. Systemic and sustained inhibition may therefore suppress pathological fibrosis while simultaneously disrupting physiological functions required for immune homeostasis and adaptive repair.

The second challenge is achieving an appropriate therapeutic window. Direct antibody-based approaches face additional pharmacological challenges. Circulating drug exposure does not necessarily reflect inhibition of locally activated TGF-β within fibrotic renal niches, and penetration of large biologics into poorly vascularized, matrix-rich interstitial regions may be heterogeneous. Conversely, increasing systemic exposure to overcome insufficient renal target engagement could increase inhibition of physiological TGF-β signaling in non-target tissues. Thus, inadequate tissue-specific pharmacodynamic engagement and systemic on-target effects represent competing constraints on dose escalation.

Patient and disease heterogeneity further complicate translation. CKD encompasses biologically distinct diseases and stages in which inflammation, epithelial injury, glomerular damage and established fibrosis contribute to progression to different extents. Enrollment based primarily on clinical diagnoses, eGFR and proteinuria may therefore combine patients with markedly different degrees of active TGF-β-dependent fibrosis. Patients with advanced irreversible scarring may have limited capacity for structural recovery even if fibrogenic signaling is successfully suppressed, whereas treatment during an early inflammatory phase may interfere with beneficial immunoregulatory and reparative TGF-β functions.

Trial design and outcome selection provide an additional challenge. Conventional endpoints such as serum creatinine, eGFR and proteinuria are clinically important but are indirect measures of fibrosis and may change slowly relative to molecular responses. A biologically active antifibrotic intervention could therefore alter renal fibrogenic activity without producing a detectable short-term change in these endpoints. The incorporation of renal imaging and urinary fibrosis markers in TOP-CKD represents an important attempt to address this limitation, although its results remain unavailable. Future studies may benefit from combining clinical outcomes with tissue, circulating, urinary and imaging biomarkers that demonstrate pathway activity and pharmacodynamic target engagement.

Taken together, the limited efficacy of previous anti-TGF-β therapies should not be interpreted as evidence that TGF-β is an invalid therapeutic target in CKD. Rather, these trials highlight the limitations of treating a spatially, temporally and cell-specific signaling network as a uniformly pathogenic pathway. This recognition provides the rationale for developing more selective and context-dependent therapeutic strategies.

## 8 Therapeutic strategies targeting TGF-β signaling in CKD

The limited clinical efficacy of systemic TGF-β inhibition does not necessarily invalidate TGF-β signaling as a therapeutic target in CKD, but rather highlights the need for greater molecular, cellular, and spatial selectivity. Therapeutic interventions can target multiple levels of the TGF-β signaling cascade, ranging from active or latent TGF-β1 and its receptors to intracellular Smad signaling and transcriptional cofactors (237). More recently, tissue- and cell-specific delivery, pathway-selective modulation, and gene- or cell-based approaches have further expanded the therapeutic landscape. These strategies differ substantially in their biological precision, preservation of homeostatic TGF-β functions, and translational maturity. The following sections therefore discuss TGF-β-targeted therapies according to their level of intervention and potential for selective modulation of pathological signaling in CKD (**Table 3**).

**Table 3:** Comparative strategies for precision modulation of TGF-β signaling in CKD.

Therapeutic strategy	Representative targets	Mechanistic rationale	Major advantages	Major limitations	Translational readiness	References
Direct ligand neutralization	Fresolimumab; LY2382770	Neutralization of extracellular TGF-β.	Direct mechanism with strong experimental antifibrotic rationale.	Limited spatial, temporal and cell specificity; may interfere with immune homeostasis and tissue repair.	Clinical trials completed; limited efficacy in CKD	(15–17)
Selective inhibition of latent TGF-β activation	SOF10; LTBP/TSP1-associated approaches	Prevents pathological activation of latent TGF-β rather than neutralizing all active ligand.	May preserve selected physiological TGF-β activation routes and improve upstream selectivity.	Kidney-specific efficacy and long-term selectivity require further validation.	Preclinical/emerging	(242)
Integrin-dependent activation blockade	Bexotegrast (PLN-74809); αvβ1/αvβ6 inhibition	Restricts integrin-mediated activation of latent TGF-β.	Targets a disease-associated activation step and may avoid complete systemic TGF-β neutralization.	Integrin functions are tissue dependent; clinical efficacy in CKD remains unestablished.	Clinically advanced in fibrotic disease; kidney translation emerging	(291, 308)
TGF-β receptor inhibition	Vactosertib (TEW-7197); TGFBR1/ALK5 inhibitors	Blocks receptor-mediated canonical and non-canonical TGF-β signaling.	Potent and pharmacologically tractable pathway inhibition.	Broad downstream suppression may compromise physiological TGF-β signaling and narrow the therapeutic window.	Preclinical to Phase I/II (in cancer)	(269, 313)
Smad pathway modulation	Smad3 inhibition; Smad7 restoration; Smad-regulated ncRNAs	Rebalances profibrotic Smad3 signaling and inhibitory/protective Smad7-associated responses.	Greater intracellular pathway selectivity than ligand neutralization.	Intracellular delivery, cell-specific effects and off-target transcriptional consequences remain challenges.	Predominantly preclinical	(20, 275)
Transcriptional cofactor modulation	HIPK2; Smad-associated transcriptional cofactors	Modifies Smad-dependent target-gene selection rather than eliminating upstream TGF-β signaling.	Potential to suppress pathological transcription while preserving physiological upstream signaling.	Cofactor functions are highly context dependent; specificity and druggability remain challenges.	Preclinical/emerging	(289, 314, 315)
β-catenin-associated transcriptional reprogramming	β-catenin–TCF/FOXO1 partner selection; ICG-001	Redirects β-catenin-associated transcription away from profibrotic programs without globally suppressing TGF-β signaling.	Directly addresses the functional duality of TGF-β and may provide a wider therapeutic window.	Existing compounds are not fully partner-selective; cell- and stage-specific effects require validation.	Proof-of-concept/preclinical with preliminary human associative evidence	(289, 303, 305, 306)
Kidney-/cell-targeted delivery	Nanoparticles, liposomes, polymers and receptor/ligand-directed carriers	Concentrates therapeutic modulation within defined renal compartments or cell populations.	May reduce systemic exposure and is compatible with small molecules and nucleic-acid therapeutics.	Biodistribution, delivery efficiency, immunogenicity and manufacturing complexity.	Preclinical/emerging	(290, 316)
Dual-target/pathway-selective modulation	Combination or multifunctional targeting of TGF-β-associated profibrotic networks; finerenone as an indirect paradigm	Addresses pathway redundancy and compensatory profibrotic signaling.	May improve efficacy without complete TGF-β suppression.	Greater pharmacodynamic complexity, potential toxicity and need for biomarker-guided selection.	Variable; some indirect strategies clinically established	(291)
Gene- and cell-based modulation	siRNA; antisense oligonucleotides; miRNA/lncRNA modulation; engineered or therapeutic cells	Enables molecularly or cellularly selective manipulation of TGF-β-associated signaling.	High target specificity and potential for durable or personalized intervention.	Delivery, durability, reversibility, manufacturing and long-term safety remain major barriers.	Early translational/preclinical	(20, 294)

### 8.1 Strategies targeting active TGF-β1

Direct inhibition of TGF-β1 remains one of the most established anti-fibrotic strategies. As noted above, therapies targeting active TGF-β1 have demonstrated significant anti-fibrotic efficacy in various animal models of CKD (238–240). However, clinical trials to date have yielded unsatisfactory outcomes. One of the major limitations of TGF-β1 neutralization lies in its failure to account for the cytokine’s context-dependent anti-inflammatory and tissue-protective roles, particularly during the early phases of injury. Alternative approaches may indirectly counteract active TGF-β1 signaling by restoring endogenous antifibrotic pathways, such as BMP7/Smad1/5/8 signaling, as discussed above (225, 241).

### 8.2 Approaches directed at latent TGF-β1

Active TGF-β1 is a potent pro-fibrotic cytokine, and strategies aimed at preventing the cleavage of latent TGF-β1 or overexpressing its precursor form have shown robust anti-fibrotic effects (242). In streptozotocin (STZ)-induced type 1 DN (T1DN) mice, overexpression of latent TGF-β1 conferred renal protection by suppressing Arkadia-mediated ubiquitin degradation of Smad7, thereby attenuating both TGF-β1/Smad3-driven fibrosis and NF-κB-mediated renal inflammation (243). Similarly, latent TGF-β1 overexpression mitigated inflammation and progressive fibrosis in the UUO mouse model (244, 245).

Thrombospondin-1 (TSP1), a key endogenous activator of TGF-β1, has been implicated in fibrosis progression; blockade of TSP1-mediated TGF-β1 activation alleviates renal fibrosis across various CKD models (246–248). Members of the integrin family also play crucial roles in regulating latent TGF-β1 activation. For instance, integrin αvβ8 expressed on mesangial cells maintains latent TGF-β1 conformation to preserve glomerular microvascular integrity (249), while integrins αv, αvβ6, αvβ3, and αvβ1 facilitate latent TGF-β1 activation and mediate pro-fibrotic responses in proximal TECs, endothelial cells, and fibroblasts (250–252).

In addition, several endogenous proteins—including ADAMTS (a disintegrin and metalloproteinase with thrombospondin motifs) family members (particularly ADAMTS1, 4, 6, and 16), ADAM9, tissue transglutaminase, extracellular matrix protein 1 (ECM1), and ROS—activate TGF-β1 by cleaving its latent precursor, thereby driving fibrogenesis (253–256). Notably, a novel antibody (LTBP-49247) that specifically targets an epitope on TGF-β1 presented by LTBP has shown selective inhibition of TGF-β1 activation and demonstrated anti-fibrotic efficacy in a rat model of adenine-induced CKD (257).

### 8.3 Inhibition of TGF-β receptor binding

Blocking the interaction between TGF-β1 and its receptors or inhibiting receptor activity represents an effective anti-fibrotic strategy. In both UUO and folic acid (FA)-induced murine models, the deubiquitinating enzyme USP11 promotes tubular cell senescence and fibrosis by preventing ubiquitin-mediated degradation of TGFBR2, whereas the USP11 inhibitor mitoxantrone (MTX) significantly ameliorates renal fibrosis (258). Similarly, inhibition of heat shock protein 90 (Hsp90) disrupts its interaction with TGFBR2, enhancing receptor ubiquitination and reducing its availability (259). A fully human monoclonal antibody targeting TGFBR2 has been shown to reduce ECM accumulation in a rat model of anti-Thy-1 nephritis (260).

In STZ-induced diabetic mice, TGF-βIIR heterozygous knockout attenuates renal injury by suppressing downstream Smad signaling and ECM gene expression (261). However, TGFBR2 deficiency also enhances NF-κB signaling and increases renal pro-inflammatory cytokine expression, likely due to the loss of TGF-β1’s anti-inflammatory effects (149). Interestingly, in a murine model of crescentic glomerulonephritis, TGFBR2 deficiency prevents kidney injury by reducing ERK pathway activation (262).

MicroRNA-based therapies have also emerged as potential TGF-β receptor modulators. For example, miR-101 and miR-92b inhibit TGF-β1-induced EMT by targeting TGFBR1, thereby alleviating renal fibrosis in CKD mouse models (263, 264). Furthermore, pharmacological inhibition of TGFBR1 using agents such as GW788388, AZ12601011, IN-1130, and R-268712 markedly improves tubulointerstitial fibrosis and glomerulosclerosis in animal models of UUO, IRI, and Thy1 nephritis (265–268). Notably, vactosertib (TEW-7197), an orally bioavailable small-molecule inhibitor of TGFBR1(ALK5), has progressed into clinical trials, primarily in patients with solid and hematological malignancies. However, its therapeutic efficacy in CKD or renal fibrosis has not yet been established clinically, highlighting the persistent translational gap between experimental TGF-β receptor blockade and its application in kidney disease (269–271).

### 8.4 Modulation of Smad-dependent signaling

Multiple Smad proteins exhibit complex and sometimes opposing roles in fibrosis. Smad3 is recognized as a key pro-fibrotic mediator; genetic deletion of Smad3 significantly protects against renal fibrosis and inflammation in models of UUO and DN (272–274). Pharmacological inhibition of Smad3 using agents such as SIS3, petchiether A, and alantolactone has shown therapeutic efficacy in attenuating renal fibrosis and inflammation (275–277). In addition, non-coding RNAs contribute to Smad3/TGF-β1 regulation—lnc-TSI and miR-15b directly target Smad3, while miR-29b and miR-200a suppress TGF-β1 expression, thereby exerting anti-fibrotic effects (20, 278, 279).

Smad7, on the other hand, acts as an endogenous inhibitor of TGF-β/Smad signaling and exerts both anti-fibrotic and anti-inflammatory functions. Upregulation of Smad7 has been shown to mitigate renal fibrosis in various murine models, including IRI, UUO, and FA-induced nephropathy (280–282). Therefore, therapeutic strategies aimed at restoring the Smad3/Smad7 balance may effectively suppress TGF-β1-driven fibrogenesis. However, such interventions should be carefully calibrated to avoid compromising anti-inflammatory defense or impairing cellular homeostasis.

### 8.5 Transcription factor-targeted strategies for modulating TGF-β1 signaling

TGF-β1 exerts its pleiotropic effects through distinct Smad proteins. Upon phosphorylation, Smads translocate into the nucleus; however, due to their low intrinsic DNA-binding affinity, they require cooperation with specific transcriptional cofactors to determine the target gene specificity of TGF-β1 responses (21). YAP and transcriptional coactivator with PDZ-binding motif (TAZ) are two closely related cofactors that promote pro-fibrotic transcription via both TGF-β–dependent and –independent mechanisms (283, 284). The tumor suppressor p53 also functions as a co-regulator of TGF-β1 signaling by associating with receptor-activated Smads to mediate transcriptional activation of fibrogenic genes (285, 286). In contrast, TG3-interacting factor (TGIF) acts as a transcriptional repressor by competing with p300 for binding to Smad2/3, thereby modulating the transcriptional output of TGF-β signaling (287, 288). Additional regulatory mechanisms further illustrate the therapeutic potential of selectively modifying Smad3-dependent transcription. Homeodomain-interacting protein kinase 2 (HIPK2) regulates profibrotic gene expression through phosphorylation of the Smad3 linker region, and allosteric HIPK2 inhibition can attenuate fibrotic transcription while preserving physiological TGF-β signaling (289). Moreover, the MAPK/ERK-inducible transcription factors EGR1 and EGR2 cooperate with Smad3 to regulate profibrotic genes, including collagen, CTGF, PAI-1, and TIMP-1, providing an additional mechanism through which signaling crosstalk shapes the transcriptional specificity of TGF-β responses (166, 203).

Among these transcriptional regulatory mechanisms, competitive β-catenin partner selection is particularly relevant to the concept of TGF-β signal reprogramming. The therapeutic implications of the β-catenin–TCF/FOXO1 axis are therefore discussed in detail in Chapter 9.

### 8.6 Emerging strategies for precision modulation of TGF-β1 signaling

The limitations of systemic TGF-β blockade have shifted therapeutic development toward greater spatial, molecular and functional selectivity. Emerging approaches include tissue- or cell-directed delivery, selective targeting of pathological activation or signaling nodes, and gene- or cell-based modulation, with the common objective of suppressing pathological TGF-β1 signaling while preserving its homeostatic and reparative functions.

#### 8.6.1 Tissue- and cell-specific delivery of TGF-β1 modulators

Kidney-targeted delivery may improve the therapeutic index of TGF-β1 modulators by enhancing drug exposure within fibrotic renal niches while limiting systemic pathway suppression. Nanoparticle-, liposome-, polymer- and ligand-based platforms can facilitate renal and cell-specific delivery; for example, megalin-dependent uptake illustrates the feasibility of targeting therapeutic cargo to proximal TECs (290). Emerging platforms may similarly deliver small molecules, peptides or nucleic-acid therapeutics to TECs, macrophages or fibrogenic stromal populations. However, CKD-associated changes in renal perfusion, filtration, tubular uptake and extracellular matrix composition may alter carrier distribution across disease stages, and most such approaches remain preclinical.

#### 8.6.2 Dual-target and pathway-selective strategies

Selective targeting of disease-associated TGF-β activation may provide greater specificity than global ligand neutralization. SOF10, a humanized antibody against latent TGF-β1, selectively inhibits protease- and integrin αvβ8-mediated activation and reduced renal fibrosis while improving kidney function in experimental CKD, with favorable preclinical safety findings (242). Similarly, bexotegrast (PLN-74809), a dual αvβ1/αvβ6 integrin inhibitor in clinical development for fibrotic lung disease, attenuated adenine- and UUO-induced kidney fibrosis, partly through inhibition of FAK/Src/Akt/β-catenin signaling (291). Although its clinical efficacy in CKD remains unestablished, these approaches illustrate the potential to target pathological TGF-β activation without directly neutralizing systemic TGF-β.

More broadly, targeting interconnected profibrotic networks may reduce compensatory signaling and permit lower exposure to individual inhibitors. Finerenone, which inhibits mineralocorticoid receptor-dependent inflammatory and fibrotic signaling, provides clinical evidence that indirect modulation of TGF-β-associated networks can slow CKD progression without direct TGF-β neutralization (292). However, multi-target approaches may increase pharmacodynamic complexity and off-target effects, supporting biomarker-guided rather than empirical pathway selection.

#### 8.6.3 Gene- and cell-based modulation of TGF-β1 signaling

Gene-based approaches may provide additional selectivity by modifying intracellular determinants of TGF-β signaling. Experimental strategies using siRNA, antisense oligonucleotides and gene-transfer systems have been explored to suppress profibrotic mediators, restore inhibitory regulators such as Smad7, or modulate TGF-β-regulated non-coding RNAs (293, 294). Such approaches may enable sustained or cell-restricted modulation but remain limited by delivery efficiency, durability, off-target effects and safety.

Cell-based therapies offer a complementary means of modifying the local inflammatory and fibrotic microenvironment. Mesenchymal stromal cells and engineered immune-cell approaches may influence TGF-β-associated signaling through paracrine or programmable mechanisms. However, variability in cell persistence, localization and phenotype, together with manufacturing and long-term safety considerations, currently limits clinical translation. Thus, gene- and cell-based strategies offer substantial potential for precision modulation but remain less translationally mature than pharmacological approaches.

### 8.7 Comparative advantages, limitations, and translational readiness

The therapeutic strategies described above differ substantially in their molecular selectivity, spatial precision, biological consequences, and translational maturity. Direct neutralization of active TGF-β provides the most straightforward means of suppressing pathway activity and has shown robust antifibrotic effects in experimental models. However, clinical experience with fresolimumab and LY2382770 has demonstrated that systemic ligand neutralization does not necessarily translate into renal benefit. In addition to incomplete target engagement within fibrotic renal niches, broad inhibition may interfere with the immunoregulatory, homeostatic, and reparative functions of TGF-β. Consequently, although direct ligand blockade is pharmacologically well characterized, its relatively narrow therapeutic window limits its attractiveness for long-term treatment of heterogeneous CKD populations.

Targeting latent TGF-β activation or receptor-proximal signaling may provide greater mechanistic selectivity. Approaches directed against integrin-dependent activation or latent TGF-β1, exemplified by bexotegrast and SOF10, aim to preferentially suppress pathological ligand activation while retaining a greater proportion of physiological TGF-β activity. Similarly, inhibition of TGFBR1 can efficiently interrupt downstream signaling, but receptor-level blockade remains intrinsically broad because both protective and pathogenic responses converge on the same receptor complex. Downstream modulation of Smad signaling or transcriptional regulators may therefore offer greater functional selectivity. In particular, targeting disease-associated Smad cofactors, HIPK2-dependent regulation, or β-catenin-associated transcriptional programs has the potential to suppress profibrotic gene expression without completely abolishing upstream TGF-β signaling (289, 295). The principal limitation of these approaches is their lower translational maturity and the context dependence of intracellular signaling networks, which may produce variable effects across renal cell types and disease stages.

Tissue-targeted delivery, nucleic-acid therapeutics, and engineered cell-based approaches offer the highest theoretical degree of spatial and cellular precision. By restricting pathway modulation to defined renal compartments or disease-associated cell states, these strategies could potentially widen the therapeutic window and minimize systemic on-target effects. However, this increased precision is accompanied by substantial translational challenges, including heterogeneous renal distribution, delivery efficiency, durability, manufacturing complexity, immunogenicity, and long-term safety. Most such approaches therefore remain preclinical, and their superiority over conventional pharmacological therapies has yet to be established in CKD.

Therefore, future development should prioritize not maximal suppression of TGF-β signaling, but an appropriate balance among antifibrotic efficacy and preservation of homeostatic signaling. This framework provides the rationale for moving from conventional pathway inhibition toward context-dependent reprogramming of TGF-β signaling output, as discussed in the following section.

## 9 TGF-β signal reprogramming as an emerging therapeutic paradigm in CKD

The dual functions of TGF-β1 and the limited clinical efficacy of systemic pathway inhibition raise a conceptually different therapeutic question: rather than determining how completely TGF-β signaling can be inhibited, can its pathological transcriptional output be selectively redirected while preserving its physiological functions? This possibility is supported by the transcriptional architecture of TGF-β signaling. Smads possess limited intrinsic DNA-binding specificity and rely on transcriptional cofactors for target-gene selection, while multiple CKD-associated signaling pathways converge on these nuclear regulatory complexes. Thus, TGF-β1 output is determined not only by ligand abundance or Smad activation, but also by the transcriptional context in which these signals operate, providing a mechanistic basis for therapeutic signal reprogramming (**Figure 4**).

### 9.1 Transcriptional cofactors as molecular determinants of TGF-β signaling output

The conceptual basis of TGF-β signal reprogramming derives from the context-dependent transcriptional functions of β-catenin. Beyond its canonical role in Wnt signaling, β-catenin acts as a transcriptional cofactor whose biological output depends on its nuclear binding partners. β-catenin–TCF/LEF signaling integrates multiple profibrotic pathways in the kidney and regulates genes involved in tubular injury, extracellular matrix remodeling and the renin–angiotensin system. Accordingly, pharmacological inhibition of β-catenin-dependent transcription with ICG-001 attenuated renal fibrosis in experimental nephropathy (296, 297).

Conversely, studies outside the kidney demonstrated that β-catenin can associate with FOXO transcription factors under oxidative stress, enhancing FOXO-dependent transcription and competing with TCF for β-catenin availability (298–301). These observations established that β-catenin is not committed to a single transcriptional program and raised the possibility that its partner selection could function as a molecular switch between alternative cellular responses. Given the extensive interaction between TGF-β/Smad and β-catenin signaling in renal injury, Zheng et al. subsequently proposed that competitive β-catenin partner selection may help determine whether TGF-β1-associated signaling favors β-catenin–TCF-dependent profibrotic transcription or β-catenin–FOXO1-associated protective programs (302).

### 9.2 Reprogramming the β-catenin–TCF/FOXO1 molecular switch

Zheng et al. subsequently tested this hypothesis across immune, renal epithelial and fibrotic contexts. In TGF-β1-induced Tregs, inhibition of β-catenin–TCF transcription with ICG-001 increased β-catenin–FOXO1 interaction, FOXO1 recruitment to the Foxp3 regulatory region and Foxp3 expression. T-cell fate mapping further demonstrated that co-administration of TGF-β1 and ICG-001 enhanced Treg-cell expansion *in vivo*. Importantly, this transcriptional redirection simultaneously reduced renal inflammation and fibrosis in UUO and IRI models (128). These experiments provided proof of concept that inhibition of the profibrotic β-catenin–TCF axis need not abolish the protective actions of TGF-β1; instead, β-catenin could be redirected toward FOXO1-dependent immunoregulatory transcription.

Subsequent studies extended this concept from immune regulation to renal fibrosis. Analysis of human kidney biopsies demonstrated greater β-catenin–TCF interaction in fibrotic kidneys, whereas β-catenin–FOXO1 interaction was associated with less fibrosis. Genetic disruption of FOXO1 or TCF1 in renal TECs further demonstrated opposing effects of these transcriptional partners on TGF-β1-induced profibrotic responses. In experimental UUO, promoting β-catenin–FOXO1 signaling by inhibiting β-catenin–TCF attenuated interstitial fibrosis (303). These findings were subsequently supported in 240 human kidney transplant biopsies, in which tubular β-catenin–FOXO1 interaction was inversely associated with chronic fibrosis and subsequent inflammatory injury, whereas β-catenin–TCF1 binding showed the opposite relationship (304).

The same principle also appears to operate in myeloid cells. Redirecting TGF-β signaling from β-catenin–TCF toward β-catenin–FOXO1 in bone marrow-derived macrophages reduced their acquisition of profibrotic characteristics and inflammatory cytokine production and attenuated renal fibrosis in UUO (305). Thus, β-catenin partner selection may influence TGF-β responses across multiple cellular compartments rather than representing a tubular epithelial cell-specific mechanism.

Importantly, the protective β-catenin–FOXO axis extends beyond suppression of fibrosis and inflammation to tissue repair. Because fibrosis represents a maladaptive extension of wound healing, complete inhibition of repair-associated signaling may itself compromise recovery. In renal epithelial wound-healing and unilateral ischemia–reperfusion models, inhibition of β-catenin–TCF with ICG-001 or iCRT3 increased β-catenin–FOXO interaction, promoted epithelial proliferation and migration, reduced TGF-β-induced mesenchymal differentiation and facilitated structural repair with less fibrosis (306). Collectively, these studies support a model in which β-catenin functions as a transcriptional molecular switch: preferential association with TCF favors persistent profibrotic transcription, whereas redirection toward FOXO1 can support immunoregulation, antifibrotic activity and adaptive epithelial repair.

Nevertheless, this model should not be interpreted as an absolute binary switch. β-catenin interacts with numerous transcriptional regulators, and the availability, localization and post-translational state of TCF, FOXO1 and other cofactors vary among cell types and injury states. Moreover, ICG-001 primarily disrupts β-catenin/CBP-dependent transcription rather than directly forcing β-catenin to bind FOXO1. Thus, existing pharmacological studies establish proof of concept for transcriptional redirection but do not yet provide a highly specific means of controlling β-catenin partner selection.

This limitation raises an important next question: can endogenous regulators of transcriptional partner selection provide a more physiologically selective means of reprogramming this molecular switch? One candidate is inhibitor of β-catenin and TCF (ICAT; encoded by CTNNBIP1), an endogenous β-catenin-binding protein that interferes with β-catenin–TCF complex formation without requiring upstream elimination of β-catenin (307). Such endogenous regulators provide a conceptual framework for selectively altering β-catenin transcriptional partnerships rather than globally suppressing β-catenin abundance or activity. Whether manipulation of endogenous partner-selection mechanisms can reproducibly redirect TGF-β signaling toward protective outputs in CKD remains to be established and represents an important direction for future investigation.

### 9.3 Translational perspectives on patient stratification and therapeutic timing

The principal translational value of TGF-β signal reprogramming lies in its potential to overcome a fundamental limitation of conventional TGF-β-targeted therapies: the inability to suppress pathological fibrosis without simultaneously disrupting the homeostatic functions of TGF-β1. Rather than reducing ligand availability or globally inhibiting receptor signaling, modulation of downstream transcriptional partner selection may selectively attenuate profibrotic output while preserving, or potentially favoring, protective programs. The β-catenin–TCF/FOXO1 axis provides a proof of concept for this strategy. Experimental studies have shown that redirecting β-catenin-associated transcription away from TCF-dependent profibrotic programs toward FOXO1-associated responses can attenuate renal fibrosis while modulating inflammatory and reparative processes (128, 303, 305, 306). Importantly, associations between β-catenin transcriptional partner selection and fibrotic outcomes have also been observed in human kidney transplant biopsies, providing preliminary clinical support for the relevance of this regulatory axis (304).

Clinical translation, however, will require identification of patients in whom pathological TGF-β-associated transcription remains active and therapeutically reversible. The benefit of signal reprogramming is unlikely to be uniform throughout CKD progression. During early injury, excessive intervention may interfere with physiological inflammatory resolution and tissue repair, whereas advanced CKD dominated by irreversible scarring may provide limited opportunity for functional recovery. The most plausible therapeutic window may therefore be the phase of active maladaptive repair and progressive fibrogenesis, when profibrotic transcriptional programs are sustained but substantial renal tissue remains salvageable. This framework also suggests that future trials should move beyond enrollment based solely on CKD stage, eGFR or proteinuria and incorporate biomarkers reflecting pathway activity and fibrogenic state.

A biomarker-guided strategy could include assessment of Smad activation, β-catenin–TCF/FOXO1-associated transcriptional states, urinary or circulating markers of active matrix remodeling, and molecular or imaging signatures of ongoing fibrosis. Single-cell and spatial profiling may further identify the renal cell populations and anatomical niches in which pathological TGF-β signaling is dominant, thereby informing both patient selection and cell- or tissue-directed delivery. Ultimately, the clinical value of TGF-β signal reprogramming will depend on demonstrating that selective modification of downstream signaling output provides a wider therapeutic window than conventional pathway blockade. Prospective validation of predictive biomarkers, pharmacodynamic evidence of target reprogramming, and demonstration of preserved physiological TGF-β functions will therefore be critical steps toward translating this concept from experimental models to precision therapy for CKD.

## 10 Conclusions and future perspectives

TGF-β1 occupies a paradoxical position in CKD, acting as a central driver of fibroblast activation, extracellular matrix accumulation and renal scarring while also contributing to immune homeostasis, resolution of inflammation and tissue repair. These divergent effects are determined by cellular source and target, anatomical compartment, disease stage, duration of signaling and interactions with downstream regulatory networks. The limited clinical efficacy of systemic TGF-β neutralization therefore highlights the limitations of treating TGF-β1 as a uniformly pathogenic mediator and supports a shift from global pathway inhibition toward context-dependent modulation.

Emerging therapeutic strategies increasingly aim to redirect pathological TGF-β signaling while preserving its homeostatic functions. The β-catenin–TCF/FOXO1 axis illustrates how transcriptional partner selection can reshape TGF-β1-associated responses, while selective Smad-associated cofactors, LRG1-mediated modulation of the ALK1/ALK5 balance, autophagy-dependent regulation and integrin-mediated control of latent TGF-β activation provide complementary mechanisms for context-selective intervention. Together, these advances suggest that therapeutic opportunities may lie not only in targeting TGF-β itself, but also in selectively modifying its activation, signaling context and downstream transcriptional output. This broader framework supports precision reprogramming of pathological TGF-β signaling rather than indiscriminate pathway suppression.

Translating this principle into CKD therapy will require spatially, temporally and molecularly informed patient stratification. Biomarkers of active TGF-β signaling, fibrogenic cell states and downstream transcriptional programs may help identify disease stages in which pathological signaling remains therapeutically reversible. Single-cell, spatial and multi-omic resources, including those generated by the Kidney Precision Medicine Project, are already providing high-resolution maps of disease-associated cell states, cellular interactions and fibrotic niches in human kidney disease. Integration of these resources with longitudinal clinical phenotyping may facilitate selection of the appropriate therapeutic target, cellular context and treatment window. Ultimately, precision reprogramming of TGF-β signaling may offer a rational strategy to limit renal inflammation and fibrosis while preserving immune homeostasis and regenerative capacity.

## References

[1] Herrington WG Judge PK Grams ME Wanner C . Chronic kidney disease. Lancet (London England). (2026) 407:90–104. doi: 10.1016/s0140-6736(25)01942-7 41314225

[2] GBD 2023 Chronic Kidney Disease Collaborators . Global, regional, and national burden of chronic kidney disease in adults, 1990-2023, and its attributable risk factors: a systematic analysis for the Global Burden of Disease Study 2023. Lancet (London England). (2025) 406:2461–82. doi: 10.1016/s0140-6736(25)01853-7 41213283

[3] Huang R Fu P Ma L . Kidney fibrosis: from mechanisms to therapeutic medicines. Signal Transduction Targeted Ther. (2023) 8:129. doi: 10.1038/s41392-023-01379-7 36932062 PMC10023808

[4] Wang H Fan K Ning Y Li Y Qi X Wang Z . Hypertension and BMI as mediators of type 2 diabetes-induced CKD: Insights from an integrative multi-database study. Diabetes Metab syndrome Obes Targets Ther. (2025) 18:3343–61. doi: 10.2147/dmso.S538554 40949084 PMC12433225

[5] Wang H Fan C Fan K Ning Y Wang Z Li Y . Impact of kidney dysfunction on outcomes in peripheral artery disease: A multi-database cohort study. iScience. (2026) 29:115681. doi: 10.1016/j.isci.2026.115681 42080135 PMC13133972

[6] Meng XM Nikolic-Paterson DJ Lan HY . TGF-β: the master regulator of fibrosis. Nat Rev Nephrol. (2016) 12:325–38. doi: 10.1038/nrneph.2016.48 27108839

[7] Zhang W Gonzalez L Li X Bai H Li Z Taniguchi R . Endothelial TGF-β signaling regulates endothelial-mesenchymal transition during arteriovenous fistula remodeling in mice with chronic kidney disease. Arteriosclerosis thrombosis Vasc Biol. (2024) 44:2509–26. doi: 10.1161/atvbaha.124.320933 39297205 PMC11593991

[8] Feng L Chen C Xiong X Wang X Li X Kuang Q . PS-MPs promotes the progression of inflammation and fibrosis in diabetic nephropathy through NLRP3/Caspase-1 and TGF-β1/Smad2/3 signaling pathways. Ecotoxicology Environ Saf. (2024) 273:116102. doi: 10.1016/j.ecoenv.2024.116102 38382346

[9] Wang S Link F Han M Chaudhary R Asimakopoulos A Liebe R . The interplay of TGF-β1 and cholesterol orchestrating hepatocyte cell fate, EMT, and signals for HSC activation. Cell Mol Gastroenterol Hepatol. (2024) 17:567–87. doi: 10.1016/j.jcmgh.2023.12.012 38154598 PMC10883985

[10] Zhou Y Tabib T Huang M Yuan K Kim Y Morse C . Molecular changes implicate angiogenesis and arterial remodeling in systemic sclerosis-associated and idiopathic pulmonary hypertension. Arteriosclerosis thrombosis Vasc Biol. (2024) 44:e210–25. doi: 10.1161/atvbaha.123.320005 38841857 PMC11269037

[11] McGaraughty S Davis-Taber RA Zhu CZ Cole TB Nikkel AL Chhaya M . Targeting anti-TGF-β therapy to fibrotic kidneys with a dual specificity antibody approach. J Am Soc Nephrol JASN. (2017) 28:3616–26. doi: 10.1681/asn.2017010013 28827403 PMC5698069

[12] Guan Q Li S Gao S Chen H Nguan CY Du C . Reduction of chronic rejection of renal allografts by anti-transforming growth factor-β antibody therapy in a rat model. Am J Physiol Renal Physiol. (2013) 305:F199–207. doi: 10.1152/ajprenal.00665.2012 23552866

[13] Peng D Fu M Wang M Wei Y Wei X . Targeting TGF-β signal transduction for fibrosis and cancer therapy. Mol Cancer. (2022) 21:104. doi: 10.1186/s12943-022-01569-x 35461253 PMC9033932

[14] Ota M Horiguchi M Fang V Shibahara K Kadota K Loomis C . Genetic suppression of inflammation blocks the tumor-promoting effects of TGF-β in gastric tissue. Cancer Res. (2014) 74:2642–51. doi: 10.1158/0008-5472.Can-13-3404 24590056 PMC4158836

[15] Voelker J Berg PH Sheetz M Duffin K Shen T Moser B . Anti-TGF-β1 antibody therapy in patients with diabetic nephropathy. J Am Soc Nephrol JASN. (2017) 28:953–62. doi: 10.1681/asn.2015111230 27647855 PMC5328150

[16] Trachtman H Fervenza FC Gipson DS Heering P Jayne DR Peters H . A phase 1, single-dose study of fresolimumab, an anti-TGF-β antibody, in treatment-resistant primary focal segmental glomerulosclerosis. Kidney Int. (2011) 79:1236–43. doi: 10.1038/ki.2011.33 21368745 PMC3257033

[17] Vincenti F Fervenza FC Campbell KN Diaz M Gesualdo L Nelson P . A phase 2, double-blind, placebo-controlled, randomized study of fresolimumab in patients with steroid-resistant primary focal segmental glomerulosclerosis. Kidney Int Rep. (2017) 2:800–10. doi: 10.1016/j.ekir.2017.03.011 29270487 PMC5733825

[18] Abbad L Esteve E Chatziantoniou C . Advances and challenges in kidney fibrosis therapeutics. Nat Rev Nephrol. (2025) 21:314–29. doi: 10.1038/s41581-025-00934-5 39934355

[19] Gu YY Liu XS Huang XR Yu XQ Lan HY . Diverse role of TGF-β in kidney disease. Front Cell Dev Biol. (2020) 8:123. doi: 10.3389/fcell.2020.00123 32258028 PMC7093020

[20] Gu YY Liu XS Lan HY . Therapeutic potential for renal fibrosis by targeting Smad3-dependent noncoding RNAs. Mol Ther J Am Soc Gene Ther. (2024) 32:313–24. doi: 10.1016/j.ymthe.2023.12.009 38093516 PMC10861968

[21] Massagué J . How cells read TGF-beta signals. Nat Rev Mol Cell Biol. (2000) 1:169–78. doi: 10.1038/35043051 11252892

[22] Morikawa M Derynck R Miyazono K . TGF-β and the TGF-β family: Context-dependent roles in cell and tissue physiology. Cold Spring Harbor Perspect Biol. (2016) 8. doi: 10.1101/cshperspect.a021873 27141051 PMC4852809

[23] Robertson IB Rifkin DB . Regulation of the bioavailability of TGF-β and TGF-β-related proteins. Cold Spring Harbor Perspect Biol. (2016) 8. doi: 10.1101/cshperspect.a021907 27252363 PMC4888822

[24] Travis MA Sheppard D . TGF-β activation and function in immunity. Annu Rev Immunol. (2014) 32:51–82. doi: 10.1146/annurev-immunol-032713-120257 24313777 PMC4010192

[25] Shi M Zhu J Wang R Chen X Mi L Walz T . Latent TGF-β structure and activation. Nature. (2011) 474:343–9. doi: 10.1038/nature10152 21677751 PMC4717672

[26] Batlle E Massagué J . Transforming growth factor-β signaling in immunity and cancer. Immunity. (2019) 50:924–40. doi: 10.1016/j.immuni.2019.03.024 30995507 PMC7507121

[27] Annes JP Chen Y Munger JS Rifkin DB . Integrin alphaVbeta6-mediated activation of latent TGF-beta requires the latent TGF-beta binding protein-1. J Cell Biol. (2004) 165:723–34. doi: 10.1083/jcb.200312172 15184403 PMC2172370

[28] Metelli A Wu BX Fugle CW Rachidi S Sun S Zhang Y . Surface expression of TGFβ docking receptor GARP promotes oncogenesis and immune tolerance in breast cancer. Cancer Res. (2016) 76:7106–17. doi: 10.1158/0008-5472.Can-16-1456 27913437 PMC5504525

[29] Rachidi S Metelli A Riesenberg B Wu BX Nelson MH Wallace C . Platelets subvert T cell immunity against cancer via GARP-TGFβ axis. Sci Immunol. (2017) 2. doi: 10.1126/sciimmunol.aai7911 28763790 PMC5539882

[30] Li L Fu H Liu Y . The fibrogenic niche in kidney fibrosis: components and mechanisms. Nat Rev Nephrol. (2022) 18:545–57. doi: 10.1038/s41581-022-00590-z 35788561

[31] Strutz F Okada H Lo CW Danoff T Carone RL Tomaszewski JE . Identification and characterization of a fibroblast marker: FSP1. J Cell Biol. (1995) 130:393–405. doi: 10.1083/jcb.130.2.393 7615639 PMC2199940

[32] Ng YY Huang TP Yang WC Chen ZP Yang AH Mu W . Tubular epithelial-myofibroblast transdifferentiation in progressive tubulointerstitial fibrosis in 5/6 nephrectomized rats. Kidney Int. (1998) 54:864–76. doi: 10.1046/j.1523-1755.1998.00076.x 9734611

[33] Srivastava SP Zhou H Setia O Dardik A Fernandez-Hernando C Goodwin J . Podocyte glucocorticoid receptors are essential for glomerular endothelial cell homeostasis in diabetes mellitus. J Am Heart Assoc. (2021) 10:e019437. doi: 10.1161/jaha.120.019437 34308664 PMC8475689

[34] Dan Hu Q Wang HL Liu J He T Tan RZ Zhang Q . Btg2 promotes focal segmental glomerulosclerosis via Smad3-dependent podocyte-mesenchymal transition. Advanced Sci (Weinheim Baden-Wurttemberg Germany). (2023) 10:e2304360. doi: 10.1002/advs.202304360 37749872 PMC10646233

[35] Liu Y . Renal fibrosis: new insights into the pathogenesis and therapeutics. Kidney Int. (2006) 69:213–7. doi: 10.1038/sj.ki.5000054 16408108

[36] Bertelli R Valenti F Oleggini R Caridi G Altieri P Coviello DA . Cell-specific regulation of alpha1(III) and alpha2(V) collagen by TGF-beta1 in tubulointerstitial cell models. Nephrology dialysis Transplant. (1998) 13:573–9. doi: 10.1093/ndt/13.3.573 9550630

[37] Fan JM Ng YY Hill PA Nikolic-Paterson DJ Mu W Atkins RC . Transforming growth factor-beta regulates tubular epithelial-myofibroblast transdifferentiation *in vitro*. Kidney Int. (1999) 56:1455–67. doi: 10.1046/j.1523-1755.1999.00656.x 10504497

[38] Nataraj D Ernst A Kalluri R . Idiopathic pulmonary fibrosis is associated with endothelial to mesenchymal transition. Am J Respir Cell Mol Biol. (2010) 43:129–30. doi: 10.1165/rcmb.2010-0044ED 20651063

[39] Maddaluno L Rudini N Cuttano R Bravi L Giampietro C Corada M . EndMT contributes to the onset and progression of cerebral cavernous malformations. Nature. (2013) 498:492–6. doi: 10.1038/nature12207 23748444

[40] Zeisberg EM Potenta SE Sugimoto H Zeisberg M Kalluri R . Fibroblasts in kidney fibrosis emerge via endothelial-to-mesenchymal transition. J Am Soc Nephrol JASN. (2008) 19:2282–7. doi: 10.1681/asn.2008050513 18987304 PMC2588112

[41] Jacobs ME de Vries DK Engelse MA Dumas SJ Rabelink TJ . Endothelial to mesenchymal transition in kidney fibrosis. Nephrology dialysis Transplant. (2024) 39:752–60. doi: 10.1093/ndt/gfad238 37968135

[42] Xavier S Vasko R Matsumoto K Zullo JA Chen R Maizel J . Curtailing endothelial TGF-β signaling is sufficient to reduce endothelial-mesenchymal transition and fibrosis in CKD. J Am Soc Nephrol JASN. (2015) 26:817–29. doi: 10.1681/asn.2013101137 25535303 PMC4378095

[43] Shi S Srivastava SP Kanasaki M He J Kitada M Nagai T . Interactions of DPP-4 and integrin β1 influences endothelial-to-mesenchymal transition. Kidney Int. (2015) 88:479–89. doi: 10.1038/ki.2015.103 25830763

[44] Gupta S Sen U . More than just an enzyme: Dipeptidyl peptidase-4 (DPP-4) and its association with diabetic kidney remodelling. Pharmacol Res. (2019) 147:104391. doi: 10.1016/j.phrs.2019.104391 31401210 PMC6733615

[45] Xu C Chen J Liang L Chen S Niu X Sang R . Midkine promotes renal fibrosis by stabilizing C/EBPβ to facilitate endothelial-mesenchymal transition. Commun Biol. (2024) 7:544. doi: 10.1038/s42003-024-06154-0 38714800 PMC11076470

[46] LeBleu VS Taduri G O'Connell J Teng Y Cooke VG Woda C . Origin and function of myofibroblasts in kidney fibrosis. Nat Med. (2013) 19:1047–53. doi: 10.1038/nm.3218 23817022 PMC4067127

[47] Diamond JR . Macrophages and progressive renal disease in experimental hydronephrosis. Am J Kidney Dis. (1995) 26:133–40. doi: 10.1016/0272-6386(95)90166-3 7611244

[48] Ume AC Wenegieme TY Shelby JN Paul-Onyia CDB Waite AMJ Kamau JK . Tacrolimus induces fibroblast-to-myofibroblast transition via a TGF-β-dependent mechanism to contribute to renal fibrosis. Am J Physiol Renal Physiol. (2023) 324:F433–45. doi: 10.1152/ajprenal.00226.2022 36927118 PMC10085566

[49] Singh SP Tao S Fields TA Webb S Harris RC Rao R . Glycogen synthase kinase-3 inhibition attenuates fibroblast activation and development of fibrosis following renal ischemia-reperfusion in mice. Dis Models Mech. (2015) 8:931–40. doi: 10.1242/dmm.020511 26092126 PMC4527294

[50] Jung SW Kim SM Kim A Park SH Moon JY Lee SH . Midbody plays an active role in fibroblast-myofibroblast transition by mediating TGF-β signaling. FASEB J. (2022) 36:e22272. doi: 10.1096/fj.202101613R 35436011

[51] Ding H Xu Z Lu Y Yuan Q Li J Sun Q . Kidney fibrosis molecular mechanisms Spp1 influences fibroblast activity through transforming growth factor beta smad signaling. iScience. (2024) 27:109839. doi: 10.1016/j.isci.2024.109839 39323737 PMC11422156

[52] Runyan CE Schnaper HW Poncelet AC . Smad3 and PKCdelta mediate TGF-beta1-induced collagen I expression in human mesangial cells. Am J Physiol Renal Physiol. (2003) 285:F413–22. doi: 10.1152/ajprenal.00082.2003 12759229

[53] Fu RG Wu JJ Xue RL Zhang T Wang L Wu XL . Premature senescence and cellular phenotype transformation of mesangial cells induced by TGF-B1. Renal Failure. (2013) 35:1142–5. doi: 10.3109/0886022x.2013.817279 23883105

[54] Mishra R Zhu L Eckert RL Simonson MS . TGF-beta-regulated collagen type I accumulation: role of Src-based signals. Am J Physiol Cell Physiol. (2007) 292:C1361–9. doi: 10.1152/ajpcell.00370.2006 17135298

[55] Mishra R Simonson MS . Oleate induces a myofibroblast-like phenotype in mesangial cells. Arteriosclerosis Thrombosis Vasc Biol. (2008) 28:541–7. doi: 10.1161/atvbaha.107.157339 18096831

[56] Xia L Wang H Munk S Kwan J Goldberg HJ Fantus IG . High glucose activates PKC-zeta and NADPH oxidase through autocrine TGF-beta1 signaling in mesangial cells. Am J Physiol Renal Physiol. (2008) 295:F1705–14. doi: 10.1152/ajprenal.00043.2008 18815221

[57] Ji C Zhang J Shi H Chen B Xu W Jin J . Single-cell RNA transcriptomic reveal the mechanism of MSC derived small extracellular vesicles against DKD fibrosis. J Nanobiotechnol. (2024) 22:339. doi: 10.1186/s12951-024-02613-2 38890734 PMC11184851

[58] Barnes JL Gorin Y . Myofibroblast differentiation during fibrosis: role of NAD(P)H oxidases. Kidney Int. (2011) 79:944–56. doi: 10.1038/ki.2010.516 21307839 PMC3675765

[59] Jiang F Liu GS Dusting GJ Chan EC . NADPH oxidase-dependent redox signaling in TGF-β-mediated fibrotic responses. Redox Biol. (2014) 2:267–72. doi: 10.1016/j.redox.2014.01.012 24494202 PMC3909817

[60] Cao Y Su H Zeng J Xie Y Liu Z Liu F . Integrin β8 prevents pericyte-myofibroblast transition and renal fibrosis through inhibiting the TGF-β1/TGFBR1/Smad3 pathway in diabetic kidney disease. Trans Res J Lab Clin Med. (2024) 265:36–50. doi: 10.1016/j.trsl.2023.10.007 37931653

[61] Yang J Wang M Zhu F Sun J Xu H Chong Lee Shin OL . Putative endothelial progenitor cells do not promote vascular repair but attenuate pericyte-myofibroblast transition in UUO-induced renal fibrosis. Stem Cell Res Ther. (2019) 10:104. doi: 10.1186/s13287-019-1201-5 30898157 PMC6429829

[62] Xu C Hong Q Zhuang K Ren X Cui S Dong Z . Regulation of pericyte metabolic reprogramming restricts the AKI to CKD transition. Metab Clin Exp. (2023) 145:155592. doi: 10.1016/j.metabol.2023.155592 37230215

[63] Chen L Li X Deng Y Chen J Huang M Zhu F . The PI3K-Akt-mTOR pathway mediates renal pericyte-myofibroblast transition by enhancing glycolysis through HKII. J Transl Med. (2023) 21:323. doi: 10.1186/s12967-023-04167-7 37179292 PMC10182641

[64] Li G Yang H Zhang D Zhang Y Liu B Wang Y . The role of macrophages in fibrosis of chronic kidney disease. Biomedicine Pharmacotherapy = Biomedecine Pharmacotherapie. (2024) 177:117079. doi: 10.1016/j.biopha.2024.117079 38968801

[65] Wang X Chen J Xu J Xie J Harris DCH Zheng G . The role of macrophages in kidney fibrosis. Front Physiol. (2021) 12:705838. doi: 10.3389/fphys.2021.705838 34421643 PMC8378534

[66] Meng XM Wang S Huang XR Yang C Xiao J Zhang Y . Inflammatory macrophages can transdifferentiate into myofibroblasts during renal fibrosis. Cell Death Dis. (2016) 7:e2495. doi: 10.1038/cddis.2016.402 27906172 PMC5261004

[67] Tang PM Nikolic-Paterson DJ Lan HY . Macrophages: versatile players in renal inflammation and fibrosis. Nat Rev Nephrol. (2019) 15:144–58. doi: 10.1038/s41581-019-0110-2 30692665

[68] Wang S Meng XM Ng YY Ma FY Zhou S Zhang Y . TGF-β/Smad3 signalling regulates the transition of bone marrow-derived macrophages into myofibroblasts during tissue fibrosis. Oncotarget. (2016) 7:8809–22. doi: 10.18632/oncotarget.6604 26684242 PMC4891006

[69] Wang Y Li Y Chen Z Yuan Y Su Q Ye K . GSDMD-dependent neutrophil extracellular traps promote macrophage-to-myofibroblast transition and renal fibrosis in obstructive nephropathy. Cell Death Dis. (2022) 13:693. doi: 10.1038/s41419-022-05138-4 35941120 PMC9360039

[70] Kramann R Machado F Wu H Kusaba T Hoeft K Schneider RK . Parabiosis and single-cell RNA sequencing reveal a limited contribution of monocytes to myofibroblasts in kidney fibrosis. JCI Insight. (2018) 3. doi: 10.1172/jci.insight.99561 29720573 PMC6012505

[71] Lever JM Hull TD Boddu R Pepin ME Black LM Adedoyin OO . Resident macrophages reprogram toward a developmental state after acute kidney injury. JCI Insight. (2019) 4. doi: 10.1172/jci.insight.125503 30674729 PMC6413788

[72] Jiang Y Wang Y Ma P An D Zhao J Liang S . Myeloid-specific targeting of Notch ameliorates murine renal fibrosis via reduced infiltration and activation of bone marrow-derived macrophage. Protein Cell. (2019) 10:196–210. doi: 10.1007/s13238-018-0527-6 29644573 PMC6338623

[73] Cheung MD Erman EN Moore KH Lever JM Li Z LaFontaine JR . Resident macrophage subpopulations occupy distinct microenvironments in the kidney. JCI Insight. (2022) 7. doi: 10.1172/jci.insight.161078 36066976 PMC9714795

[74] Lazarov T Juarez-Carreño S Cox N Geissmann F . Physiology and diseases of tissue-resident macrophages. Nature. (2023) 618:698–707. doi: 10.1038/s41586-023-06002-x 37344646 PMC10649266

[75] Dick SA Wong A Hamidzada H Nejat S Nechanitzky R Vohra S . Three tissue resident macrophage subsets coexist across organs with conserved origins and life cycles. Sci Immunol. (2022) 7:eabf7777. doi: 10.1126/sciimmunol.abf7777 34995099

[76] Cheung MD Agarwal A George JF . Where are they now: spatial and molecular diversity of tissue-resident macrophages in the kidney. Semin Nephrol. (2022) 42:151276. doi: 10.1016/j.semnephrol.2022.10.002 36435683

[77] Zhang YL Tang TT Wang B Wen Y Feng Y Yin Q . Identification of a novel ECM remodeling macrophage subset in AKI to CKD transition by integrative spatial and single-cell analysis. Advanced Sci (Weinheim Baden-Wurttemberg Germany). (2024) 11:e2309752. doi: 10.1002/advs.202309752 39119903 PMC11481374

[78] Humphreys BD Lin SL Kobayashi A Hudson TE Nowlin BT Bonventre JV . Fate tracing reveals the pericyte and not epithelial origin of myofibroblasts in kidney fibrosis. Am J Pathol. (2010) 176:85–97. doi: 10.2353/ajpath.2010.090517 20008127 PMC2797872

[79] Lin SL Kisseleva T Brenner DA Duffield JS . Pericytes and perivascular fibroblasts are the primary source of collagen-producing cells in obstructive fibrosis of the kidney. Am J Pathol. (2008) 173:1617–27. doi: 10.2353/ajpath.2008.080433 19008372 PMC2626374

[80] Kuppe C Ibrahim MM Kranz J Zhang X Ziegler S Perales-Patón J . Decoding myofibroblast origins in human kidney fibrosis. Nature. (2021) 589:281–6. doi: 10.1038/s41586-020-2941-1 33176333 PMC7611626

[81] Melkonian AL Cheung MD Erman EN Moore KH Lever JMP Jiang Y . Single-cell RNA sequencing and spatial transcriptomics reveal unique subpopulations of infiltrating macrophages and dendritic cells following AKI. Am J Physiol Renal Physiol. (2025) 328:F907–20. doi: 10.1152/ajprenal.00059.2025 40331777

[82] Liu J Zheng B Cui Q Zhu Y Chu L Geng Z . Single-cell spatial transcriptomics unveils platelet-fueled cycling macrophages for kidney fibrosis. Advanced Sci (Weinheim Baden-Wurttemberg Germany). (2024) 11:e2308505. doi: 10.1002/advs.202308505 38838052 PMC11304276

[83] He S Xia T Guo Z Song X Wang H He H . Tracing the origin of myofibroblasts in kidney fibrosis. Nat Commun. (2025) 17:653. doi: 10.1038/s41467-025-67373-5 41423454 PMC12815922

[84] Liu F Qiu H Xue M Zhang S Zhang X Xu J . MSC-secreted TGF-β regulates lipopolysaccharide-stimulated macrophage M2-like polarization via the Akt/FoxO1 pathway. Stem Cell Res Ther. (2019) 10:345. doi: 10.1186/s13287-019-1447-y 31771622 PMC6878630

[85] Qin YY Huang XR Zhang J Wu W Chen J Wan S . Neuropeptide Y attenuates cardiac remodeling and deterioration of function following myocardial infarction. Mol Ther J Am Soc Gene Ther. (2022) 30:881–97. doi: 10.1016/j.ymthe.2021.10.005 34628054 PMC8821956

[86] Zhang F Wang H Wang X Jiang G Liu H Zhang G . TGF-β induces M2-like macrophage polarization via SNAIL-mediated suppression of a pro-inflammatory phenotype. Oncotarget. (2016) 7:52294–306. doi: 10.18632/oncotarget.10561 27418133 PMC5239552

[87] Lei Z Tang R Wu Y Mao C Xue W Shen J . TGF-β1 induces PD-1 expression in macrophages through SMAD3/STAT3 cooperative signaling in chronic inflammation. JCI Insight. (2024) 9. doi: 10.1172/jci.insight.165544 38441961 PMC11128204

[88] Chen B Huang S Su Y Wu YJ Hanna A Brickshawana A . Macrophage Smad3 protects the infarcted heart, stimulating phagocytosis and regulating inflammation. Circ Res. (2019) 125:55–70. doi: 10.1161/circresaha.119.315069 31092129 PMC6681442

[89] Chung S Overstreet JM Li Y Wang Y Niu A Wang S . TGF-β promotes fibrosis after severe acute kidney injury by enhancing renal macrophage infiltration. JCI Insight. (2018) 3. doi: 10.1172/jci.insight.123563 30385721 PMC6238749

[90] Cao Q Wang Y Zheng D Sun Y Wang Y Lee VW . IL-10/TGF-beta-modified macrophages induce regulatory T cells and protect against adriamycin nephrosis. J Am Soc Nephrol JASN. (2010) 21:933–42. doi: 10.1681/asn.2009060592 20299353 PMC2900959

[91] Luo L Wang S Hu Y Wang L Jiang X Zhang J . Precisely regulating M2 subtype macrophages for renal fibrosis resolution. ACS Nano. (2023) 17:22508–26. doi: 10.1021/acsnano.3c05998 37948096

[92] Meng XM Wang L Nikolic-Paterson DJ Lan HY . Innate immune cells in acute and chronic kidney disease. Nat Rev Nephrol. (2025) 21(7):464–82. doi: 10.1038/s41581-025-00958-x 40263532

[93] Rogers NM Ferenbach DA Isenberg JS Thomson AW Hughes J . Dendritic cells and macrophages in the kidney: a spectrum of good and evil. Nat Rev Nephrol. (2014) 10:625–43. doi: 10.1038/nrneph.2014.170 25266210 PMC4922410

[94] Salei N Rambichler S Salvermoser J Papaioannou NE Schuchert R Pakalniškytė D . The kidney contains ontogenetically distinct dendritic cell and macrophage subtypes throughout development that differ in their inflammatory properties. J Am Soc Nephrol JASN. (2020) 31:257–78. doi: 10.1681/asn.2019040419 31932472 PMC7003301

[95] Kassianos AJ Wang X Sampangi S Muczynski K Healy H Wilkinson R . Increased tubulointerstitial recruitment of human CD141(hi) CLEC9A(+) and CD1c(+) myeloid dendritic cell subsets in renal fibrosis and chronic kidney disease. Am J Physiol Renal Physiol. (2013) 305:F1391–401. doi: 10.1152/ajprenal.00318.2013 24049150

[96] Hashimoto M Yanagisawa H Minagawa S Sen D Ma R Murray LA . TGF-β-dependent dendritic cell chemokinesis in murine models of airway disease. J Immunol (Baltimore Md 1950). (2015) 195:1182–90. doi: 10.4049/jimmunol.1500348 26109638 PMC4506848

[97] Seeger P Musso T Sozzani S . The TGF-β superfamily in dendritic cell biology. Cytokine Growth Factor Rev. (2015) 26:647–57. doi: 10.1016/j.cytogfr.2015.06.002 26115564

[98] Kurts C Ginhoux F Panzer U . Kidney dendritic cells: fundamental biology and functional roles in health and disease. Nat Rev Nephrol. (2020) 16:391–407. doi: 10.1038/s41581-020-0272-y 32372062

[99] Thepmalee C Panya A Junking M Chieochansin T Yenchitsomanus PT . Inhibition of IL-10 and TGF-β receptors on dendritic cells enhances activation of effector T-cells to kill cholangiocarcinoma cells. Hum Vaccines Immunotherapeutics. (2018) 14:1423–31. doi: 10.1080/21645515.2018.1431598 29420117 PMC6037468

[100] Owyang SY Zhang M El-Zaatari M Eaton KA Bishu S Hou G . Dendritic cell-derived TGF-β mediates the induction of mucosal regulatory T-cell response to Helicobacter infection essential for maintenance of immune tolerance in mice. Helicobacter. (2020) 25:e12763. doi: 10.1111/hel.12763 33025641 PMC7885176

[101] Zheng D Wen L Li C Peng A Cao Q Wang Y . Adoptive transfer of bone marrow dendritic cells failed to localize in the renal cortex and to improve renal injury in adriamycin nephropathy. Nephron Exp Nephrol. (2014) 126:8–15. doi: 10.1159/000358086 24526139

[102] Nakazawa D Masuda S Nishibata Y Watanabe-Kusunoki K Tomaru U Ishizu A . Neutrophils and NETs in kidney disease. Nat Rev Nephrol. (2025) 21:383–98. doi: 10.1038/s41581-025-00944-3 40102634

[103] Wang H Gao T Zhang R Hu J Gao S Wang Y . Neutrophil extracellular traps aggravate contrast-induced acute kidney injury by damaging glomeruli and peritubular capillaries. J Inflammation Res. (2023) 16:5629–46. doi: 10.2147/jir.S433110 38046404 PMC10693253

[104] Ryu S Shin JW Kwon S Lee J Kim YC Bae YS . Siglec-F-expressing neutrophils are essential for creating a profibrotic microenvironment in renal fibrosis. J Clin Invest. (2022) 132. doi: 10.1172/jci156876 35482420 PMC9197522

[105] Fridlender ZG Sun J Kim S Kapoor V Cheng G Ling L . Polarization of tumor-associated neutrophil phenotype by TGF-beta: "N1" versus "N2" TAN. Cancer Cell. (2009) 16:183–94. doi: 10.1016/j.ccr.2009.06.017 19732719 PMC2754404

[106] Peng H Shen J Long X Zhou X Zhang J Xu X . Local release of TGF-β inhibitor modulates tumor-associated neutrophils and enhances pancreatic cancer response to combined irreversible electroporation and immunotherapy. Advanced Sci (Weinheim Baden-Wurttemberg Germany). (2022) 9:e2105240. doi: 10.1002/advs.202105240 35128843 PMC8981446

[107] Hein LE SenGupta S Gunasekaran G Johnson CN Parent CA . TGF-β1 activates neutrophil signaling and gene expression but not migration. PloS One. (2023) 18:e0290886. doi: 10.1371/journal.pone.0290886 37682817 PMC10490904

[108] Torres LS Chweih H Fabris FCZ Gotardo EMF Leonardo FC Saad STO . TGF-β1 reduces neutrophil adhesion and prevents acute vaso-occlusive processes in sickle cell disease mice. Cells. (2022) 11. doi: 10.3390/cells11071200 35406764 PMC8998040

[109] Lee K Jang HR Rabb H . Lymphocytes and innate immune cells in acute kidney injury and repair. Nat Rev Nephrol. (2024) 20:789–805. doi: 10.1038/s41581-024-00875-5 39095505

[110] Kohei N Tanaka T Tanabe K Masumori N Dvorina N Valujskikh A . Natural killer cells play a critical role in mediating inflammation and graft failure during antibody-mediated rejection of kidney allografts. Kidney Int. (2016) 89:1293–306. doi: 10.1016/j.kint.2016.02.030 27165816 PMC4868788

[111] Kim HJ Lee JS Kim JD Cha HJ Kim A Lee SK . Reverse signaling through the costimulatory ligand CD137L in epithelial cells is essential for natural killer cell-mediated acute tissue inflammation. PNAS. (2012) 109:E13–22. doi: 10.1073/pnas.1112256109 22160719 PMC3252952

[112] Law BMP Wilkinson R Wang X Kildey K Lindner M Rist MJ . Interferon-γ production by tubulointerstitial human CD56(bright) natural killer cells contributes to renal fibrosis and chronic kidney disease progression. Kidney Int. (2017) 92:79–88. doi: 10.1016/j.kint.2017.02.006 28396119

[113] Castriconi R Dondero A Bellora F Moretta L Castellano A Locatelli F . Neuroblastoma-derived TGF-β1 modulates the chemokine receptor repertoire of human resting NK cells. J Immunol (Baltimore Md 1950). (2013) 190:5321–8. doi: 10.4049/jimmunol.1202693 23576682

[114] Trotta R Dal Col J Yu J Ciarlariello D Thomas B Zhang X . TGF-beta utilizes SMAD3 to inhibit CD16-mediated IFN-gamma production and antibody-dependent cellular cytotoxicity in human NK cells. J Immunol (Baltimore Md 1950). (2008) 181:3784–92. doi: 10.4049/jimmunol.181.6.3784 18768831 PMC2924753

[115] Yu J Wei M Becknell B Trotta R Liu S Boyd Z . Pro- and antiinflammatory cytokine signaling: reciprocal antagonism regulates interferon-gamma production by human natural killer cells. Immunity. (2006) 24:575–90. doi: 10.1016/j.immuni.2006.03.016 16713975

[116] Lian GY Wang QM Mak TS Huang XR Yu XQ Lan HY . Disrupting Smad3 potentiates immunostimulatory function of NK cells against lung carcinoma by promoting GM-CSF production. Cell Mol Life Sci CMLS. (2024) 81:262. doi: 10.1007/s00018-024-05290-4 38878186 PMC11335298

[117] Stangou M Bantis C Skoularopoulou M Korelidou L Kouloukouriotou D Scina M . Th1, Th2 and Treg/T17 cytokines in two types of proliferative glomerulonephritis. Indian J Nephrol. (2016) 26:159–66. doi: 10.4103/0971-4065.159303 27194829 PMC4862260

[118] Kitching AR Turner AL Wilson GR Semple T Odobasic D Timoshanko JR . IL-12p40 and IL-18 in crescentic glomerulonephritis: IL-12p40 is the key Th1-defining cytokine chain, whereas IL-18 promotes local inflammation and leukocyte recruitment. J Am Soc Nephrol JASN. (2005) 16:2023–33. doi: 10.1681/asn.2004121075 15888563

[119] Liu L Kou P Zeng Q Pei G Li Y Liang H . CD4+ T lymphocytes, especially Th2 cells, contribute to the progress of renal fibrosis. Am J Nephrol. (2012) 36:386–96. doi: 10.1159/000343283 23052013

[120] Chen B Mu C Zhang Z He X Liu X . The love-hate relationship between TGF-β signaling and the immune system during development and tumorigenesis. Front Immunol. (2022) 13:891268. doi: 10.3389/fimmu.2022.891268 35720407 PMC9204485

[121] Crowl JT Heeg M Ferry A Milner JJ Omilusik KD Toma C . Tissue-resident memory CD8(+) T cells possess unique transcriptional, epigenetic and functional adaptations to different tissue environments. Nat Immunol. (2022) 23:1121–31. doi: 10.1038/s41590-022-01229-8 35761084 PMC10041538

[122] van der Leij J van den Berg A Albrecht EW Blokzijl T Roozendaal R Gouw AS . High expression of TIAF-1 in chronic kidney and liver allograft rejection and in activated T-helper cells. Transplantation. (2003) 75:2076–82. doi: 10.1097/01.Tp.0000069829.71088.88 12829915

[123] Song T Eirin A Zhu X Zhao Y Krier JD Tang H . Mesenchymal stem cell-derived extracellular vesicles induce regulatory T cells to ameliorate chronic kidney injury. Hypertension (Dallas Tex 1979). (2020) 75:1223–32. doi: 10.1161/hypertensionaha.119.14546 32223383 PMC7219723

[124] Mortazavi H Soltani-Zangbar MS Eghbal-Fard S Mehdizadeh A Kamrani A Chakeri-Khiavi F . Cytokine profile, Treg/Th17 cell frequency changes during different posttransplantational time points in patients undergoing renal transplantation. J Cell Physiol. (2019) 234:20935–43. doi: 10.1002/jcp.28698 31012117

[125] Viklicky O Hribova P Volk HD Slatinska J Petrasek J Bandur S . Molecular phenotypes of acute rejection predict kidney graft prognosis. J Am Soc Nephrol JASN. (2010) 21:173–80. doi: 10.1681/asn.2008121268 19797166 PMC2799284

[126] Liao T Xue Y Zhao D Li S Liu M Chen J . *In vivo* attenuation of antibody-mediated acute renal allograft rejection by ex vivo TGF-β-induced CD4(+)Foxp3(+) regulatory T cells. Front Immunol. (2017) 8:1334. doi: 10.3389/fimmu.2017.01334 29085374 PMC5650643

[127] Ma X Ding J Min H Wen Y Gao Q . Protective role of low-dose TGF-β1 in early diabetic nephropathy induced by streptozotocin. Int Immunopharmacol. (2013) 17:752–8. doi: 10.1016/j.intimp.2013.08.023 24055008

[128] Qiao X Rao P Zhang Y Liu L Pang M Wang H . Redirecting TGF-β signaling through the β-catenin/Foxo complex prevents kidney fibrosis. J Am Soc Nephrol JASN. (2018) 29:557–70. doi: 10.1681/asn.2016121362 29180394 PMC5791062

[129] Cai J Jiao X Zhao S Liang Y Ning Y Shi Y . Transforming growth factor-β1-overexpressing mesenchymal stromal cells induced local tolerance in rat renal ischemia/reperfusion injury. Cytotherapy. (2019) 21:535–45. doi: 10.1016/j.jcyt.2018.12.003 30685215

[130] Horwitz DA Bickerton S Koss M Fahmy TM La Cava A . Suppression of murine lupus by CD4+ and CD8+ Treg cells induced by T cell-targeted nanoparticles loaded with interleukin-2 and transforming growth factor β. Arthritis Rheumatol (Hoboken NJ). (2019) 71:632–40. doi: 10.1002/art.40773 30407752 PMC6438734

[131] Pineda S Sigdel TK Liberto JM Vincenti F Sirota M Sarwal MM . Characterizing pre-transplant and post-transplant kidney rejection risk by B cell immune repertoire sequencing. Nat Commun. (2019) 10:1906. doi: 10.1038/s41467-019-09930-3 31015506 PMC6479061

[132] Einecke G Reeve J Mengel M Sis B Bunnag S Mueller TF . Expression of B cell and immunoglobulin transcripts is a feature of inflammation in late allografts. Am J Transplant. (2008) 8:1434–43. doi: 10.1111/j.1600-6143.2008.02232.x 18444922

[133] Kreimann K Jang MS Rong S Greite R von Vietinghoff S Schmitt R . Ischemia reperfusion injury triggers CXCL13 release and B-cell recruitment after allogenic kidney transplantation. Front Immunol. (2020) 11:1204. doi: 10.3389/fimmu.2020.01204 32849490 PMC7424013

[134] Reese SR Wilson NA Huang Y Ptak L Degner KR Xiang D . B-cell deficiency attenuates transplant glomerulopathy in a rat model of chronic active antibody-mediated rejection. Transplantation. (2021) 105:1516–29. doi: 10.1097/tp.0000000000003530 33273321 PMC8106694

[135] Cheung CK Barratt J Lafayette R Liew A Suzuki Y Tesař V . Targeting APRIL in the treatment of glomerular diseases. Kidney Int. (2024) 106:806–18. doi: 10.1016/j.kint.2024.08.012 39182759

[136] Cippà PE Liu J Sun B Kumar S Naesens M McMahon AP . A late B lymphocyte action in dysfunctional tissue repair following kidney injury and transplantation. Nat Commun. (2019) 10:1157. doi: 10.1038/s41467-019-09092-2 30858375 PMC6411919

[137] Jang HR Gandolfo MT Ko GJ Satpute SR Racusen L Rabb H . B cells limit repair after ischemic acute kidney injury. J Am Soc Nephrol JASN. (2010) 21:654–65. doi: 10.1681/asn.2009020182 20203156 PMC2844308

[138] Li X Yang W Ma K Zheng Z Liu X Hu B . Circulating B cell-derived small RNA delivered by extracellular vesicles: a dialogue mechanism for long-range targeted renal mitochondrial injury in obesity. Small (Weinheim der Bergstrasse Germany). (2024) 20:e2402526. doi: 10.1002/smll.202402526 38958071

[139] Kim AH Chung JJ Akilesh S Koziell A Jain S Hodgin JB . B cell-derived IL-4 acts on podocytes to induce proteinuria and foot process effacement. JCI Insight. (2017) 2. doi: 10.1172/jci.insight.81836 29093269 PMC5752294

[140] Xu Y Wei Z Feng M Zhu D Mei S Wu Z . Tumor-infiltrated activated B cells suppress liver metastasis of colorectal cancers. Cell Rep. (2022) 40:111295. doi: 10.1016/j.celrep.2022.111295 36044847

[141] Wallace CH Wu BX Salem M Ansa-Addo EA Metelli A Sun S . B lymphocytes confer immune tolerance via cell surface GARP-TGF-β complex. JCI Insight. (2018) 3. doi: 10.1172/jci.insight.99863 29618665 PMC5928869

[142] Xu A Liu Y Chen W Wang J Xue Y Huang F . TGF-β-induced regulatory T cells directly suppress B cell responses through a noncytotoxic mechanism. J Immunol (Baltimore Md 1950). (2016) 196:3631–41. doi: 10.4049/jimmunol.1501740 27001954 PMC4868785

[143] Zhong H Liu Y Xu Z Liang P Yang H Zhang X . TGF-β-induced CD8(+)CD103(+) regulatory T cells show potent therapeutic effect on chronic graft-versus-host disease lupus by suppressing B cells. Front Immunol. (2018) 9:35. doi: 10.3389/fimmu.2018.00035 29441062 PMC5797539

[144] Long W Zhang H Yuan W Lan G Lin Z Peng L . The role of regulatory B cells in kidney diseases. Front Immunol. (2021) 12:683926. doi: 10.3389/fimmu.2021.683926 34108975 PMC8183681

[145] Song H Kim Y Park G Kim YS Kim S Lee HK . Transforming growth factor-β1 regulates human renal proximal tubular epithelial cell susceptibility to natural killer cells via modulation of the NKG2D ligands. Int J Mol Med. (2015) 36:1180–8. doi: 10.3892/ijmm.2015.2317 26311146

[146] Banu N Meyers CM . TGF-beta1 down-regulates induced expression of both class II MHC and B7-1 on primary murine renal tubular epithelial cells. Kidney Int. (1999) 56:985–94. doi: 10.1046/j.1523-1755.1999.00645.x 10469366

[147] Kayhan M Vouillamoz J Rodriguez DG Bugarski M Mitamura Y Gschwend J . Intrinsic TGF-β signaling attenuates proximal tubule mitochondrial injury and inflammation in chronic kidney disease. Nat Commun. (2023) 14:3236. doi: 10.1038/s41467-023-39050-y 37270534 PMC10239443

[148] Pawluczyk IZ Harris KP . Transforming growth factor-beta suppresses macrophage-induced mesangial cell fibronectin expression. Kidney Int. (2001) 60:533–42. doi: 10.1046/j.1523-1755.2001.060002533.x 11473636

[149] Meng XM Huang XR Xiao J Chen HY Zhong X Chung AC . Diverse roles of TGF-β receptor II in renal fibrosis and inflammation *in vivo* and *in vitro*. J Pathol. (2012) 227:175–88. doi: 10.1002/path.3976 22190171

[150] Abedini A Levinsohn J Klötzer KA Dumoulin B Ma Z Frederick J . Single-cell multi-omic and spatial profiling of human kidneys implicates the fibrotic microenvironment in kidney disease progression. Nat Genet. (2024) 56:1712–24. doi: 10.1038/s41588-024-01802-x 39048792 PMC11592391

[151] Zhong X Chung AC Chen HY Meng XM Lan HY . Smad3-mediated upregulation of miR-21 promotes renal fibrosis. J Am Soc Nephrol JASN. (2011) 22:1668–81. doi: 10.1681/asn.2010111168 21852586 PMC3171938

[152] McClelland AD Herman-Edelstein M Komers R Jha JC Winbanks CE Hagiwara S . miR-21 promotes renal fibrosis in diabetic nephropathy by targeting PTEN and SMAD7. Clin Sci (London Engl 1979). (2015) 129:1237–49. doi: 10.1042/cs20150427 26415649

[153] Feng M Tang PM Huang XR Sun SF You YK Xiao J . TGF-β mediates renal fibrosis via the Smad3-Erbb4-IR long noncoding RNA axis. Mol Ther J Am Soc Gene Ther. (2018) 26:148–61. doi: 10.1016/j.ymthe.2017.09.024 29102563 PMC5763082

[154] Wang P Luo ML Song E Zhou Z Ma T Wang J . Long noncoding RNA lnc-TSI inhibits renal fibrogenesis by negatively regulating the TGF-β/Smad3 pathway. Sci Transl Med. (2018) 10. doi: 10.1126/scitranslmed.aat2039 30305452

[155] MacDonald BT Tamai K He X . Wnt/beta-catenin signaling: components, mechanisms, and diseases. Dev Cell. (2009) 17:9–26. doi: 10.1016/j.devcel.2009.06.016 19619488 PMC2861485

[156] Yang Y Mlodzik M . Wnt-Frizzled/planar cell polarity signaling: cellular orientation by facing the wind (Wnt). Annu Rev Cell Dev Biol. (2015) 31:623–46. doi: 10.1146/annurev-cellbio-100814-125315 26566118 PMC4673888

[157] Liu J Xiao Q Xiao J Niu C Li Y Zhang X . Wnt/β-catenin signalling: function, biological mechanisms, and therapeutic opportunities. Signal Transduction Targeted Ther. (2022) 7:3. doi: 10.1038/s41392-021-00762-6 34980884 PMC8724284

[158] Jiang X Charlat O Zamponi R Yang Y Cong F . Dishevelled promotes Wnt receptor degradation through recruitment of ZNRF3/RNF43 E3 ubiquitin ligases. Mol Cell. (2015) 58:522–33. doi: 10.1016/j.molcel.2015.03.015 25891077

[159] Schunk SJ Floege J Fliser D Speer T . WNT-β-catenin signalling - a versatile player in kidney injury and repair. Nat Rev Nephrol. (2021) 17:172–84. doi: 10.1038/s41581-020-00343-w 32989282

[160] Feng Y Ren J Gui Y Wei W Shu B Lu Q . Wnt/β-catenin-promoted macrophage alternative activation contributes to kidney fibrosis. J Am Soc Nephrol JASN. (2018) 29:182–93. doi: 10.1681/asn.2017040391 29021383 PMC5748914

[161] Feng Y Liang Y Zhu X Wang M Gui Y Lu Q . The signaling protein Wnt5a promotes TGFβ1-mediated macrophage polarization and kidney fibrosis by inducing the transcriptional regulators Yap/Taz. J Biol Chem. (2018) 293:19290–302. doi: 10.1074/jbc.RA118.005457 30333225 PMC6302175

[162] Działo E Czepiel M Tkacz K Siedlar M Kania G Błyszczuk P . WNT/β-catenin signaling promotes TGF-β-mediated activation of human cardiac fibroblasts by enhancing IL-11 production. Int J Mol Sci. (2021) 22. doi: 10.3390/ijms221810072 34576234 PMC8468519

[163] Blyszczuk P Müller-Edenborn B Valenta T Osto E Stellato M Behnke S . Transforming growth factor-β-dependent Wnt secretion controls myofibroblast formation and myocardial fibrosis progression in experimental autoimmune myocarditis. Eur Heart J. (2017) 38:1413–25. doi: 10.1093/eurheartj/ehw116 27099262

[164] Xiang FL Fang M Yutzey KE . Loss of β-catenin in resident cardiac fibroblasts attenuates fibrosis induced by pressure overload in mice. Nat Commun. (2017) 8:712. doi: 10.1038/s41467-017-00840-w 28959037 PMC5620049

[165] Johnson GL Lapadat R . Mitogen-activated protein kinase pathways mediated by ERK, JNK, and p38 protein kinases. Sci (New York NY). (2002) 298:1911–2. doi: 10.1126/science.1072682 12471242

[166] Livingston MJ Zhang M Kwon SH Chen JK Li H Manicassamy S . Autophagy activates EGR1 via MAPK/ERK to induce FGF2 in renal tubular cells for fibroblast activation and fibrosis during maladaptive kidney repair. Autophagy. (2024) 20:1032–53. doi: 10.1080/15548627.2023.2281156 37978868 PMC11135847

[167] Juan YH Yu YL Tsai YP Lee CC Chen YS Ting YH . Alpha-mangostin alleviate renal interstitial fibrosis via suppression of TGF-β1/Smad/ERK signaling axis *in vitro* and *in vivo*. Biochem Pharmacol. (2023) 218:115935. doi: 10.1016/j.bcp.2023.115935 37989414

[168] Yuan Q Tang B Zhang C . Signaling pathways of chronic kidney diseases, implications for therapeutics. Signal Transduction Targeted Ther. (2022) 7:182. doi: 10.1038/s41392-022-01036-5 35680856 PMC9184651

[169] Lee MK Pardoux C Hall MC Lee PS Warburton D Qing J . TGF-beta activates Erk MAP kinase signalling through direct phosphorylation of ShcA. EMBO J. (2007) 26:3957–67. doi: 10.1038/sj.emboj.7601818 17673906 PMC1994119

[170] Wang Y Yang H Su X Cao A Chen F Chen P . TGF-β1/SMOC2/AKT and ERK axis regulates proliferation, migration, and fibroblast to myofibroblast transformation in lung fibroblast, contributing with the asthma progression. Hereditas. (2021) 158:47. doi: 10.1186/s41065-021-00213-w 34876240 PMC8653533

[171] Ungefroren H Witte D Fiedler C Gädeken T Kaufmann R Lehnert H . The role of PAR2 in TGF-β1-induced ERK activation and cell motility. Int J Mol Sci. (2017) 18. doi: 10.3390/ijms18122776 29261154 PMC5751374

[172] Yamashita M Fatyol K Jin C Wang X Liu Z Zhang YE . TRAF6 mediates Smad-independent activation of JNK and p38 by TGF-beta. Mol Cell. (2008) 31:918–24. doi: 10.1016/j.molcel.2008.09.002 18922473 PMC2621323

[173] Lu S Chen X Chen Y Zhang Y Luo J Jiang H . Downregulation of PDZK1 by TGF-β1 promotes renal fibrosis via inducing epithelial-mesenchymal transition of renal tubular cells. Biochem Pharmacol. (2024) 220:116015. doi: 10.1016/j.bcp.2023.116015 38158021

[174] Rhyu DY Yang Y Ha H Lee GT Song JS Uh ST . Role of reactive oxygen species in TGF-beta1-induced mitogen-activated protein kinase activation and epithelial-mesenchymal transition in renal tubular epithelial cells. J Am Soc Nephrol JASN. (2005) 16:667–75. doi: 10.1681/asn.2004050425 15677311

[175] Li J Qin X Xu W Zhang H Huang S Yang Y . Herb pair of Rhubarb-Astragalus mitigates renal interstitial fibrosis through downregulation of autophagy via p38-MAPK/TGF-β1 and p38-MAPK/smad2/3 pathways. Int J Biochem Cell Biol. (2024) 169:106549. doi: 10.1016/j.biocel.2024.106549 38340950

[176] Tao Y Tang C Wei J Shan Y Fang X Li Y . Nr4a1 promotes renal interstitial fibrosis by regulating the p38 MAPK phosphorylation. Mol Med (Cambridge Mass). (2023) 29:63. doi: 10.1186/s10020-023-00657-y 37161357 PMC10169452

[177] Zhang J Cao L Wang X Li Q Zhang M Cheng C . The E3 ubiquitin ligase TRIM31 plays a critical role in hypertensive nephropathy by promoting proteasomal degradation of MAP3K7 in the TGF-β1 signaling pathway. Cell Death Differ. (2022) 29:556–67. doi: 10.1038/s41418-021-00874-0 34584221 PMC8901735

[178] Su Z Zimpelmann J Burns KD . Angiotensin-(1-7) inhibits angiotensin II-stimulated phosphorylation of MAP kinases in proximal tubular cells. Kidney Int. (2006) 69:2212–8. doi: 10.1038/sj.ki.5001509 16672906

[179] Alzayadneh EM Chappell MC . Angiotensin-(1-7) abolishes AGE-induced cellular hypertrophy and myofibroblast transformation via inhibition of ERK1/2. Cell Signalling. (2014) 26:3027–35. doi: 10.1016/j.cellsig.2014.09.010 25246357 PMC4254268

[180] Carvajal G Rodríguez-Vita J Rodrigues-Díez R Sánchez-López E Rupérez M Cartier C . Angiotensin II activates the Smad pathway during epithelial mesenchymal transdifferentiation. Kidney Int. (2008) 74:585–95. doi: 10.1038/ki.2008.213 18509316

[181] Kamato D Burch ML Piva TJ Rezaei HB Rostam MA Xu S . Transforming growth factor-β signalling: role and consequences of Smad linker region phosphorylation. Cell Signalling. (2013) 25:2017–24. doi: 10.1016/j.cellsig.2013.06.001 23770288

[182] Huang Y Noble NA Zhang J Xu C Border WA . Renin-stimulated TGF-beta1 expression is regulated by a mitogen-activated protein kinase in mesangial cells. Kidney Int. (2007) 72:45–52. doi: 10.1038/sj.ki.5002243 17396111

[183] Raman A Reif GA Dai Y Khanna A Li X Astleford L . Integrin-linked kinase signaling promotes cyst growth and fibrosis in polycystic kidney disease. J Am Soc Nephrol JASN. (2017) 28:2708–19. doi: 10.1681/asn.2016111235 28522687 PMC5576939

[184] Campillo S Gutiérrez-Calabrés E García-Miranda S Griera M Fernández Rodríguez L de Frutos S . Integrin-linked kinase mRNA expression in circulating mononuclear cells as a biomarker of kidney and vascular damage in experimental chronic kidney disease. Cell communication Signaling CCS. (2024) 22:264. doi: 10.1186/s12964-024-01646-2 38734696 PMC11088758

[185] Huang Q Sun Y Peng L Sun J Sha Z Lin H . Extracellular vesicle-packaged ILK from mesothelial cells promotes fibroblast activation in peritoneal fibrosis. J Extracell Vesicles. (2023) 12:e12334. doi: 10.1002/jev2.12334 37357686 PMC10291285

[186] Gonzalez-Ramos M de Frutos S Griera M Luengo A Olmos G Rodriguez-Puyol D . Integrin-linked kinase mediates the hydrogen peroxide-dependent transforming growth factor-β1 up-regulation. Free Radical Biol Med. (2013) 61:416–27. doi: 10.1016/j.freeradbiomed.2013.04.029 23624332

[187] Li Y Yang J Dai C Wu C Liu Y . Role for integrin-linked kinase in mediating tubular epithelial to mesenchymal transition and renal interstitial fibrogenesis. J Clin Invest. (2003) 112:503–16. doi: 10.1172/jci17913 12925691 PMC171389

[188] Li Y Tan X Dai C Stolz DB Wang D Liu Y . Inhibition of integrin-linked kinase attenuates renal interstitial fibrosis. J Am Soc Nephrol JASN. (2009) 20:1907–18. doi: 10.1681/asn.2008090930 19541809 PMC2736781

[189] Liu Y . New insights into epithelial-mesenchymal transition in kidney fibrosis. J Am Soc Nephrol JASN. (2010) 21:212–22. doi: 10.1681/asn.2008121226 20019167 PMC4554339

[190] Zheng G Zhang J Zhao H Wang H Pang M Qiao X . α3 integrin of cell-cell contact mediates kidney fibrosis by integrin-linked kinase in proximal tubular E-cadherin deficient mice. Am J Pathol. (2016) 186:1847–60. doi: 10.1016/j.ajpath.2016.03.015 27182643

[191] Pang M Wang H Rao P Zhao Y Xie J Cao Q . Autophagy links β-catenin and Smad signaling to promote epithelial-mesenchymal transition via upregulation of integrin linked kinase. Int J Biochem Cell Biol. (2016) 76:123–34. doi: 10.1016/j.biocel.2016.05.010 27177845

[192] Yamamura Y Sakai N Iwata Y Lagares D Hara A Kitajima S . Myocardin-related transcription factor contributes to renal fibrosis through the regulation of extracellular microenvironment surrounding fibroblasts. FASEB J. (2023) 37:e23005. doi: 10.1096/fj.202201870R 37289107

[193] Jiménez-Uribe AP Gómez-Sierra T Aparicio-Trejo OE Orozco-Ibarra M Pedraza-Chaverri J . Backstage players of fibrosis: NOX4, mTOR, HDAC, and S1P; companions of TGF-β. Cell Signalling. (2021) 87:110123. doi: 10.1016/j.cellsig.2021.110123 34438016

[194] Chen M Yu Y Mi T Guo Q Xiang B Tian X . MK-2206 alleviates renal fibrosis by suppressing the Akt/mTOR signaling pathway *in vivo* and *in vitro*. Cells. (2022) 11. doi: 10.3390/cells11213505 36359901 PMC9655032

[195] Li J Ren J Liu X Jiang L He W Yuan W . Rictor/mTORC2 signaling mediates TGFβ1-induced fibroblast activation and kidney fibrosis. Kidney Int. (2015) 88:515–27. doi: 10.1038/ki.2015.119 25970154 PMC4558569

[196] Ren J Li J Feng Y Shu B Gui Y Wei W . Rictor/mammalian target of rapamycin complex 2 promotes macrophage activation and kidney fibrosis. J Pathol. (2017) 242:488–99. doi: 10.1002/path.4921 28585302

[197] Hermida MA Dinesh Kumar J Leslie NR . GSK3 and its interactions with the PI3K/AKT/mTOR signalling network. Adv Biol Regul. (2017) 65:5–15. doi: 10.1016/j.jbior.2017.06.003 28712664

[198] Evangelisti C Chiarini F Paganelli F Marmiroli S Martelli AM . Crosstalks of GSK3 signaling with the mTOR network and effects on targeted therapy of cancer. Biochim Biophys Acta Mol Cell Res. (2020) 1867:118635. doi: 10.1016/j.bbamcr.2019.118635 31884070

[199] Ren J Li J Liu X Feng Y Gui Y Yang J . Quercetin inhibits fibroblast activation and kidney fibrosis involving the suppression of mammalian target of rapamycin and β-catenin signaling. Sci Rep. (2016) 6:23968. doi: 10.1038/srep23968 27052477 PMC4823739

[200] Tang Q Markby GR MacNair AJ Tang K Tkacz M Parys M . TGF-β-induced PI3K/AKT/mTOR pathway controls myofibroblast differentiation and secretory phenotype of valvular interstitial cells through the modulation of cellular senescence in a naturally occurring *in vitro* canine model of myxomatous mitral valve disease. Cell Proliferation. (2023) 56:e13435. doi: 10.1111/cpr.13435 36869852 PMC10280140

[201] Livingston MJ Shu S Fan Y Li Z Jiao Q Yin XM . Tubular cells produce FGF2 via autophagy after acute kidney injury leading to fibroblast activation and renal fibrosis. Autophagy. (2023) 19:256–77. doi: 10.1080/15548627.2022.2072054 35491858 PMC9809951

[202] Ding Y Choi ME . Regulation of autophagy by TGF-β: emerging role in kidney fibrosis. Semin Nephrol. (2014) 34:62–71. doi: 10.1016/j.semnephrol.2013.11.009 24485031 PMC3912517

[203] Kökény G Németh Á Kopp JB Chen W Oler AJ Manzéger A . Susceptibility to kidney fibrosis in mice is associated with early growth response-2 protein and tissue inhibitor of metalloproteinase-1 expression. Kidney Int. (2022) 102:337–54. doi: 10.1016/j.kint.2022.03.029 35513123 PMC9393427

[204] Zhuang S Liu N . EGFR signaling in renal fibrosis. Kidney Int Suppl. (2014) 4:70–4. doi: 10.1038/kisup.2014.13 26312153 PMC4536964

[205] Samarakoon R Dobberfuhl AD Cooley C Overstreet JM Patel S Goldschmeding R . Induction of renal fibrotic genes by TGF-β1 requires EGFR activation, p53 and reactive oxygen species. Cell Signalling. (2013) 25:2198–209. doi: 10.1016/j.cellsig.2013.07.007 23872073

[206] Docherty NG O'Sullivan OE Healy DA Murphy M O'Neill AJ Fitzpatrick JM . TGF-beta1-induced EMT can occur independently of its proapoptotic effects and is aided by EGF receptor activation. Am J Physiol Renal Physiol. (2006) 290:F1202–12. doi: 10.1152/ajprenal.00406.2005 16368739

[207] Chen J Chen JK Neilson EG Harris RC . Role of EGF receptor activation in angiotensin II-induced renal epithelial cell hypertrophy. J Am Soc Nephrol JASN. (2006) 17:1615–23. doi: 10.1681/asn.2005111163 16641152

[208] Wang TN Chen X Li R Gao B Mohammed-Ali Z Lu C . SREBP-1 mediates angiotensin II-induced TGF-β1 upregulation and glomerular fibrosis. J Am Soc Nephrol JASN. (2015) 26:1839–54. doi: 10.1681/asn.2013121332 25398788 PMC4520156

[209] Chen Y Peng FF Jin J Chen HM Yu H Zhang BF . Src-mediated ligand release-independent EGFR transactivation involves TGF-β-induced Smad3 activation in mesangial cells. Biochem Biophys Res Commun. (2017) 493:914–20. doi: 10.1016/j.bbrc.2017.09.121 28943431

[210] Overstreet JM Wang Y Wang X Niu A Gewin LS Yao B . Selective activation of epidermal growth factor receptor in renal proximal tubule induces tubulointerstitial fibrosis. FASEB J. (2017) 31:4407–21. doi: 10.1096/fj.201601359RR 28626027 PMC5602893

[211] Zhu X Meng X Du X Zhao C Ma X Wen Y . Vaccarin suppresses diabetic nephropathy through inhibiting the EGFR/ERK1/2 signaling pathway. Acta Biochim Biophys Sin. (2024) 56:1860–74. doi: 10.3724/abbs.2024141 39205643 PMC11972988

[212] Uttarwar L Peng F Wu D Kumar S Gao B Ingram AJ . HB-EGF release mediates glucose-induced activation of the epidermal growth factor receptor in mesangial cells. Am J Physiol Renal Physiol. (2011) 300:F921–31. doi: 10.1152/ajprenal.00436.2010 21289053

[213] Wu D Peng F Zhang B Ingram AJ Kelly DJ Gilbert RE . PKC-beta1 mediates glucose-induced Akt activation and TGF-beta1 upregulation in mesangial cells. J Am Soc Nephrol JASN. (2009) 20:554–66. doi: 10.1681/asn.2008040445 19211711 PMC2653673

[214] Chen J Chen JK Harris RC . EGF receptor deletion in podocytes attenuates diabetic nephropathy. J Am Soc Nephrol JASN. (2015) 26:1115–25. doi: 10.1681/asn.2014020192 25185988 PMC4413759

[215] Zhou B Lin W Long Y Yang Y Zhang H Wu K . Notch signaling pathway: architecture, disease, and therapeutics. Signal Transduction Targeted Ther. (2022) 7:95. doi: 10.1038/s41392-022-00934-y 35332121 PMC8948217

[216] Zavadil J Cermak L Soto-Nieves N Böttinger EP . Integration of TGF-beta/Smad and Jagged1/Notch signalling in epithelial-to-mesenchymal transition. EMBO J. (2004) 23:1155–65. doi: 10.1038/sj.emboj.7600069 14976548 PMC380966

[217] Ueno T Kobayashi N Nakayama M Takashima Y Ohse T Pastan I . Aberrant Notch1-dependent effects on glomerular parietal epithelial cells promotes collapsing focal segmental glomerulosclerosis with progressive podocyte loss. Kidney Int. (2013) 83:1065–75. doi: 10.1038/ki.2013.48 23447065 PMC7567340

[218] Tung CW Hsu YC Cai CJ Shih YH Wang CJ Chang PJ . Trichostatin A ameliorates renal tubulointerstitial fibrosis through modulation of the JNK-dependent Notch-2 signaling pathway. Sci Rep. (2017) 7:14495. doi: 10.1038/s41598-017-15162-6 29101337 PMC5670251

[219] Zhao Y Qiao X Tan TK Zhao H Zhang Y Liu L . Matrix metalloproteinase 9-dependent Notch signaling contributes to kidney fibrosis through peritubular endothelial-mesenchymal transition. Nephrology Dialysis Transplant. (2017) 32:781–91. doi: 10.1093/ndt/gfw308 27566305 PMC5427520

[220] Tattersall IW Du J Cong Z Cho BS Klein AM Dieck CL . *In vitro* modeling of endothelial interaction with macrophages and pericytes demonstrates Notch signaling function in the vascular microenvironment. Angiogenesis. (2016) 19:201–15. doi: 10.1007/s10456-016-9501-1 26965898 PMC5576170

[221] Nyhan KC Faherty N Murray G Cooey LB Godson C Crean JK . Jagged/Notch signalling is required for a subset of TGFβ1 responses in human kidney epithelial cells. Biochim Biophys Acta. (2010) 1803:1386–95. doi: 10.1016/j.bbamcr.2010.09.001 20833210

[222] Niranjan T Bielesz B Gruenwald A Ponda MP Kopp JB Thomas DB . The Notch pathway in podocytes plays a role in the development of glomerular disease. Nat Med. (2008) 14:290–8. doi: 10.1038/nm1731 18311147

[223] Wang H Li Y Zheng G . BMP7 in kidney development and disease: a multilayer regulatory network integrating inflammation, fibrosis, and regeneration. Cell Signaling Inflammation Dis. (2026) 1:2. doi: 10.1186/s44505-026-00002-038164791

[224] Awazu M Nagata M Hida M . BMP7 dose-dependently stimulates proliferation and cadherin-11 expression via ERK and p38 in a murine metanephric mesenchymal cell line. Physiol Rep. (2017) 5. doi: 10.14814/phy2.13378 28867673 PMC5582263

[225] Peng W Zhou X Xu T Mao Y Zhang X Liu H . BMP-7 ameliorates partial epithelial-mesenchymal transition by restoring SnoN protein level via Smad1/5 pathway in diabetic kidney disease. Cell Death Dis. (2022) 13:254. doi: 10.1038/s41419-022-04529-x 35314669 PMC8938433

[226] Wang H Gao T Fan K Fan C Dong H Zheng G . Compensatory protective potential of parietal epithelial cell-to-podocyte transdifferentiation. Kidney Int. (2025) 108:709–10. doi: 10.1016/j.kint.2025.06.002 40975528

[227] Gravesen E Lerche Mace M Nordholm A Hofman-Bang J Hruska K Haagen Nielsen C . Exogenous BMP7 in aortae of rats with chronic uremia ameliorates expression of profibrotic genes, but does not reverse established vascular calcification. PloS One. (2018) 13:e0190820. doi: 10.1371/journal.pone.0190820 29304096 PMC5755916

[228] Wang Y Xiao Y Li S Shi L Liu L Zhang Y . BMP-7 enhances SnoN mRNA expression in renal tubular epithelial cells under high-glucose conditions. Mol Med Rep. (2017) 16:3308–14. doi: 10.3892/mmr.2017.7025 28765970 PMC5548011

[229] Higgins DF Ewart LM Masterson E Tennant S Grebnev G Prunotto M . BMP7-induced-Pten inhibits Akt and prevents renal fibrosis. Biochim Biophys Acta Mol Basis Dis. (2017) 1863:3095–104. doi: 10.1016/j.bbadis.2017.09.011 28923783

[230] Shen B Liu X Fan Y Qiu J . Macrophages regulate renal fibrosis through modulating TGFβ superfamily signaling. Inflammation. (2014) 37:2076–84. doi: 10.1007/s10753-014-9941-y 24929555

[231] Meng XM Chung AC Lan HY . Role of the TGF-β/BMP-7/Smad pathways in renal diseases. Clin Sci (London Engl 1979). (2013) 124:243–54. doi: 10.1042/cs20120252 23126427

[232] Bongiovanni C Bueno-Levy H Posadas Pena D Del Bono I Miano C Boriati S . BMP7 promotes cardiomyocyte regeneration in zebrafish and adult mice. Cell Rep. (2024) 43:114162. doi: 10.1016/j.celrep.2024.114162 38678558

[233] Wang H Dhaliwal NS Zahin M Zhao T Sims GL Xie J . The clinical effectiveness of anti-TGF-β therapy in chronic kidney disease: a systematic review of Western and Traditional Chinese Medicine. Integr Med Nephrol Andrology. (2025) 12. doi: 10.1097/IMNA-D-24-0006442585464

[234] Cho ME Smith DC Branton MH Penzak SR Kopp JB . Pirfenidone slows renal function decline in patients with focal segmental glomerulosclerosis. Clin J Am Soc Nephrol CJASN. (2007) 2:906–13. doi: 10.2215/cjn.01050207 17702727

[235] Sharma K Ix JH Mathew AV Cho M Pflueger A Dunn SR . Pirfenidone for diabetic nephropathy. J Am Soc Nephrol JASN. (2011) 22:1144–51. doi: 10.1681/asn.2010101049 21511828 PMC3103734

[236] Yu D Zhang R Zhou J Guo P Li P Ye M . Pharmacokinetics and safety of pirfenidone in individuals with chronic kidney disease stage G2 and G3a: a single-dose, Phase I, bridging study. J Pharm Sci. (2025) 114:1087–94. doi: 10.1016/j.xphs.2024.11.020 39694267

[237] Hong Q Kim H Cai GY Chen XM He JC Lee K . Modulation of TGF-β signaling new approaches toward kidney disease and fibrosis therapy. Int J Biol Sci. (2025) 21:1649–65. doi: 10.7150/ijbs.101548 39990662 PMC11844295

[238] Liang X Schnaper HW Matsusaka T Pastan I Ledbetter S Hayashida T . Anti-TGF-β antibody, 1D11, ameliorates glomerular fibrosis in mouse models after the onset of proteinuria. PloS One. (2016) 11:e0155534. doi: 10.1371/journal.pone.0155534 27187580 PMC4871338

[239] Dahly AJ Hoagland KM Flasch AK Jha S Ledbetter SR Roman RJ . Antihypertensive effects of chronic anti-TGF-beta antibody therapy in Dahl S rats. Am J Physiol Regul Intgr Comp Physiol. (2002) 283:R757–67. doi: 10.1152/ajpregu.00098.2002 12185011

[240] Lavoie P Robitaille G Agharazii M Ledbetter S Lebel M Larivière R . Neutralization of transforming growth factor-beta attenuates hypertension and prevents renal injury in uremic rats. J Hypertens. (2005) 23:1895–903. doi: 10.1097/01.hjh.0000182521.44440.c5 16148614

[241] Cai C Kilari S Singh AK Zhao C Simeon ML Misra A . Differences in transforming growth factor-β1/BMP7 signaling and venous fibrosis contribute to female sex differences in arteriovenous fistulas. J Am Heart Assoc. (2020) 9:e017420. doi: 10.1161/jaha.120.017420 32757791 PMC7660821

[242] Kanamori M Sato I Koo CX Sun Y Kawauchi H Nakagawa K . Selective blockade of latent TGF-β1 activation suppresses tissue fibrosis with good safety. Commun Med. (2026) 6. doi: 10.1038/s43856-026-01408-w 41606188 PMC12953895

[243] Wu W Huang XR You Y Xue L Wang XJ Meng X . Latent TGF-β1 protects against diabetic kidney disease via Arkadia/Smad7 signaling. Int J Biol Sci. (2021) 17:3583–94. doi: 10.7150/ijbs.61647 34512167 PMC8416717

[244] Huang XR Chung AC Wang XJ Lai KN Lan HY . Mice overexpressing latent TGF-beta1 are protected against renal fibrosis in obstructive kidney disease. Am J Physiol Renal Physiol. (2008) 295:F118–27. doi: 10.1152/ajprenal.00021.2008 18448597 PMC2494503

[245] Wang W Huang XR Li AG Liu F Li JH Truong LD . Signaling mechanism of TGF-beta1 in prevention of renal inflammation: role of Smad7. J Am Soc Nephrol JASN. (2005) 16:1371–83. doi: 10.1681/asn.2004121070 15788474

[246] Ponticelli C Anders HJ . Thrombospondin immune regulation and the kidney. Nephrology Dialysis Transplant. (2017) 32:1084–9. doi: 10.1093/ndt/gfw431 28088772

[247] Lu A Miao M Schoeb TR Agarwal A Murphy-Ullrich JE . Blockade of TSP1-dependent TGF-β activity reduces renal injury and proteinuria in a murine model of diabetic nephropathy. Am J Pathol. (2011) 178:2573–86. doi: 10.1016/j.ajpath.2011.02.039 21641382 PMC3124297

[248] Zhou Y Ng DYE Richards AM Wang P . microRNA-221 inhibits latent TGF-β1 activation through targeting thrombospondin-1 to attenuate kidney failure-induced cardiac fibrosis. Mol Ther Nucleic Acids. (2020) 22:803–14. doi: 10.1016/j.omtn.2020.09.041 33230477 PMC7645417

[249] Lakhe-Reddy S Li V Arnold TD Khan S Schelling JR . Mesangial cell αvβ8-integrin regulates glomerular capillary integrity and repair. Am J Physiol Renal Physiol. (2014) 306:F1400–9. doi: 10.1152/ajprenal.00624.2013 24740792 PMC4059974

[250] Geng H Lan R Singha PK Gilchrist A Weinreb PH Violette SM . Lysophosphatidic acid increases proximal tubule cell secretion of profibrotic cytokines PDGF-B and CTGF through LPA2- and Gαq-mediated Rho and αvβ6 integrin-dependent activation of TGF-β. Am J Pathol. (2012) 181:1236–49. doi: 10.1016/j.ajpath.2012.06.035 22885106 PMC3463629

[251] Shen Y Miao N Wang B Xu J Gan X Xu D . c-Myc promotes renal fibrosis by inducing integrin αv-mediated transforming growth factor-β signaling. Kidney Int. (2017) 92:888–99. doi: 10.1016/j.kint.2017.03.006 28483378

[252] Hinz B . It has to be the αv: myofibroblast integrins activate latent TGF-β1. Nat Med. (2013) 19:1567–8. doi: 10.1038/nm.3421 24309651

[253] Umeda M Karino K Satyam A Yoshida N Hisada R Bhargava R . Hypoxia promotes the expression of ADAM9 by tubular epithelial cells, which enhances transforming growth factor β1 activation and promotes tissue fibrosis in patients with lupus nephritis. Arthritis Rheumatol (Hoboken NJ). (2025) 77:180–9. doi: 10.1002/art.42987 39279154 PMC11785507

[254] Xiao L Li Q Chen S Huang Y Ma L Wang Y . ADAMTS16 drives epithelial-mesenchymal transition and metastasis through a feedback loop upon TGF-β1 activation in lung adenocarcinoma. Cell Death Dis. (2024) 15:837. doi: 10.1038/s41419-024-07226-z 39551781 PMC11570625

[255] Link F Li Y Zhao J Munker S Fan W Nwosu ZC . ECM1 attenuates hepatic fibrosis by interfering with mediators of latent TGF-β1 activation. Gut. (2025) 74:424–39. doi: 10.1136/gutjnl-2024-333213 39448254

[256] Vistnes M Erusappan PM Sasi A Nordén ES Bergo KK Romaine A . Inhibition of the extracellular enzyme A disintegrin and metalloprotease with thrombospondin motif 4 prevents cardiac fibrosis and dysfunction. Cardiovasc Res. (2023) 119:1915–27. doi: 10.1093/cvr/cvad078 37216909 PMC10439713

[257] Jackson JW Frederick CS Jr. Pal A Coricor G Boston C Brueckner CT . An antibody that inhibits TGF-β1 release from latent extracellular matrix complexes attenuates the progression of renal fibrosis. Sci Signaling. (2024) 17:eadn6052. doi: 10.1126/scisignal.adn6052 38980922

[258] Ni JY Wang X Xie HY Yang NH Li JY Sun XA . Deubiquitinating enzyme USP11 promotes renal tubular cell senescence and fibrosis via inhibiting the ubiquitin degradation of TGF-β receptor II. Acta Pharmacol Sin. (2023) 44:584–95. doi: 10.1038/s41401-022-00977-5 36045219 PMC9958121

[259] Noh H Kim HJ Yu MR Kim WY Kim J Ryu JH . Heat shock protein 90 inhibitor attenuates renal fibrosis through degradation of transforming growth factor-β type II receptor. Lab Investigation; A J Tech Methods Pathol. (2012) 92:1583–96. doi: 10.1038/labinvest.2012.127 22964853

[260] Kasuga H Ito Y Sakamoto S Kawachi H Shimizu F Yuzawa Y . Effects of anti-TGF-beta type II receptor antibody on experimental glomerulonephritis. Kidney Int. (2001) 60:1745–55. doi: 10.1046/j.1523-1755.2001.00990.x 11703592

[261] Kim HW Kim BC Song CY Kim JH Hong HK Lee HS . Heterozygous mice for TGF-betaIIR gene are resistant to the progression of streptozotocin-induced diabetic nephropathy. Kidney Int. (2004) 66:1859–65. doi: 10.1111/j.1523-1755.2004.00959.x 15496156

[262] Song CY Kim BC Hong HK Lee HS . TGF-beta type II receptor deficiency prevents renal injury via decrease in ERK activity in crescentic glomerulonephritis. Kidney Int. (2007) 71:882–8. doi: 10.1038/sj.ki.5002149 17299519

[263] Wang Q Tao Y Xie H Liu C Liu P . MicroRNA-101 inhibits renal tubular epithelial-to-mesenchymal transition by targeting TGF-β1 type I receptor. Int J Mol Med. (2021) 47. doi: 10.3892/ijmm.2021.4952 33955520 PMC8099196

[264] Yang S Jiang K Li L Xiang J Li Y Kang L . MircroRNA-92b as a negative regulator of the TGF-β signaling by targeting the type I receptor. iScience. (2023) 26:108131. doi: 10.1016/j.isci.2023.108131 37867958 PMC10587525

[265] Suo XG Wang F Xu CH He XY Wang JN Zhang Y . Targeted inhibition of TGF-β type I receptor by AZ12601011 protects against kidney fibrosis. Eur J Pharmacol. (2022) 929:175116. doi: 10.1016/j.ejphar.2022.175116 35780825

[266] Moon JA Kim HT Cho IS Sheen YY Kim DK . IN-1130, a novel transforming growth factor-beta type I receptor kinase (ALK5) inhibitor, suppresses renal fibrosis in obstructive nephropathy. Kidney Int. (2006) 70:1234–43. doi: 10.1038/sj.ki.5001775 16929250

[267] Terashima H Kato M Ebisawa M Kobayashi H Suzuki K Nezu Y . R-268712, an orally active transforming growth factor-β type I receptor inhibitor, prevents glomerular sclerosis in a Thy1 nephritis model. Eur J Pharmacol. (2014) 734:60–6. doi: 10.1016/j.ejphar.2014.03.045 24726873

[268] Petersen M Thorikay M Deckers M van Dinther M Grygielko ET Gellibert F . Oral administration of GW788388, an inhibitor of TGF-beta type I and II receptor kinases, decreases renal fibrosis. Kidney Int. (2008) 73:705–15. doi: 10.1038/sj.ki.5002717 18075500

[269] Park SA Kim MJ Park SY Kim JS Lee SJ Woo HA . EW-7197 inhibits hepatic, renal, and pulmonary fibrosis by blocking TGF-β/Smad and ROS signaling. Cell Mol Life Sci CMLS. (2015) 72:2023–39. doi: 10.1007/s00018-014-1798-6 25487606 PMC11113926

[270] Ha KB Sangartit W Jeong AR Lee ES Kim HM Shim S . EW-7197 attenuates the progression of diabetic nephropathy in db/db mice through suppression of fibrogenesis and inflammation. Endocrinol Metab (Seoul Korea). (2022) 37:96–111. doi: 10.3803/EnM.2021.1305 35255604 PMC8901963

[271] Jang SY Hwang SH Choi Y Kim WY Park SH Kim WM . Evaluation of the novel ALK5 inhibitor EW-7197 on therapeutic efficacy in renal fibrosis using a three-dimensional chip model. Kidney Res Clin Pract. (2025) 44:612–25. doi: 10.23876/j.krcp.23.324 39384357 PMC12245568

[272] You YK Wu WF Huang XR Li HD Ren YP Zeng JC . Deletion of Smad3 protects against C-reactive protein-induced renal fibrosis and inflammation in obstructive nephropathy. Int J Biol Sci. (2021) 17:3911–22. doi: 10.7150/ijbs.62929 34671208 PMC8495386

[273] Xu BH Sheng J You YK Huang XR Ma RCW Wang Q . Deletion of Smad3 prevents renal fibrosis and inflammation in type 2 diabetic nephropathy. Metab Clin Exp. (2020) 103:154013. doi: 10.1016/j.metabol.2019.154013 31734275

[274] Wang HL Wei B He HJ Huang XR Sheng JY Chen XC . Smad3 deficiency improves islet-based therapy for diabetes and diabetic kidney injury by promoting β cell proliferation via the E2F3-dependent mechanism. Theranostics. (2022) 12:379–95. doi: 10.7150/thno.67034 34987651 PMC8690916

[275] He H Wang H Chen X Zhong Y Huang XR Ma RC . Treatment for type 2 diabetes and diabetic nephropathy by targeting Smad3 signaling. Int J Biol Sci. (2024) 20:200–17. doi: 10.7150/ijbs.87820 38164169 PMC10750285

[276] You YK Luo Q Wu WF Zhang JJ Zhu HJ Lao L . Petchiether A attenuates obstructive nephropathy by suppressing TGF-β/Smad3 and NF-κB signalling. J Cell Mol Med. (2019) 23:5576–87. doi: 10.1111/jcmm.14454 31211499 PMC6652659

[277] Lee KM Hwang YJ Jung GS . Alantolactone attenuates renal fibrosis via inhibition of transforming growth factor β/Smad3 signaling pathway. Diabetes Metab J. (2024) 48:72–82. doi: 10.4093/dmj.2022.0231 38173367 PMC10850280

[278] Liu J Xiong Y Mo H Niu H Miao J Shen W . MicroRNA-29b plays a vital role in podocyte injury and glomerular diseases through inducing mitochondrial dysfunction. Int J Biol Sci. (2024) 20:4654–73. doi: 10.7150/ijbs.93506 39309435 PMC11414390

[279] Chen HY Zhong X Huang XR Meng XM You Y Chung AC . MicroRNA-29b inhibits diabetic nephropathy in db/db mice. Mol Ther J Am Soc Gene Ther. (2014) 22:842–53. doi: 10.1038/mt.2013.235 24445937 PMC3982502

[280] Li L Lee J Cho A Kim JH Ju W An JN . cMet agonistic antibody prevents acute kidney injury to chronic kidney disease transition by suppressing Smurf1 and activating Smad7. Clin Sci (London Engl 1979). (2021) 135:1427–44. doi: 10.1042/cs20210013 34061176

[281] Gong M Guo Y Dong H Wu W Wu F Lu F . Trigonelline inhibits tubular epithelial-mesenchymal transformation in diabetic kidney disease via targeting Smad7. Biomedicine Pharmacotherapy = Biomedecine Pharmacotherapie. (2023) 168:115747. doi: 10.1016/j.biopha.2023.115747 37864898

[282] Tao S Yang L Wu C Hu Y Guo F Ren Q . Gambogenic acid alleviates kidney fibrosis via epigenetic inhibition of EZH2 to regulate Smad7-dependent mechanism. Phytomedicine Int J Phytotherapy Phytopharmacology. (2022) 106:154390. doi: 10.1016/j.phymed.2022.154390 35994849

[283] Zhang T He X Caldwell L Goru SK Ulloa Severino L Tolosa MF . NUAK1 promotes organ fibrosis via YAP and TGF-β/SMAD signaling. Sci Transl Med. (2022) 14:eaaz4028. doi: 10.1126/scitranslmed.aaz4028 35320001

[284] Szeto SG Narimatsu M Lu M He X Sidiqi AM Tolosa MF . YAP/TAZ are mechanoregulators of TGF-β-Smad signaling and renal fibrogenesis. J Am Soc Nephrol JASN. (2016) 27:3117–28. doi: 10.1681/asn.2015050499 26961347 PMC5042658

[285] Higgins CE Tang J Mian BM Higgins SP Gifford CC Conti DJ . TGF-β1-p53 cooperativity regulates a profibrotic genomic program in the kidney: molecular mechanisms and clinical implications. FASEB J. (2019) 33:10596–606. doi: 10.1096/fj.201900943R 31284746 PMC6766640

[286] Higgins CE Tang J Higgins SP Gifford CC Mian BM Jones DM . The genomic response to TGF-β1 dictates failed repair and progression of fibrotic disease in the obstructed kidney. Front Cell Dev Biol. (2021) 9:678524. doi: 10.3389/fcell.2021.678524 34277620 PMC8284093

[287] Nakashima H Tsujimura K Irie K Ishizu M Pan M Kameda T . Canonical TGF-β signaling negatively regulates neuronal morphogenesis through TGIF/Smad complex-mediated CRMP2 suppression. J Neurosci. (2018) 38:4791–810. doi: 10.1523/jneurosci.2423-17.2018 29695415 PMC6596019

[288] Shepherd RD Kos SM Rinker KD . Flow-dependent Smad2 phosphorylation and TGIF nuclear localization in human aortic endothelial cells. Am J Physiol Heart Circ Physiol. (2011) 301:H98–H107. doi: 10.1152/ajpheart.00668.2010 21490324 PMC3129916

[289] Lee K Drakas R He JC . Small molecule allosteric inhibitor of HIPK2 as a novel therapy against kidney fibrosis. J Am Soc Nephrol JASN. (2024) 35:809–11. doi: 10.1681/asn.0000000000000327 39288913 PMC11164111

[290] Nastase MV Zeng-Brouwers J Wygrecka M Schaefer L . Targeting renal fibrosis: mechanisms and drug delivery systems. Adv Drug Delivery Rev. (2018) 129:295–307. doi: 10.1016/j.addr.2017.12.019 29288033

[291] Mu Y Liu J Wu Q Wang B Hu T Li Y . A dual αvβ1/αvβ6 integrin inhibitor Bexotegrast (PLN-74809) ameliorates organ injury and fibrogenesis in fibrotic kidney disease. Eur J Pharmacol. (2024) 983:176983. doi: 10.1016/j.ejphar.2024.176983 39243926

[292] Piko N Bevc S Hojs R Ekart R . Finerenone: From the mechanism of action to clinical use in kidney disease. Pharm (Basel Switzerland). (2024) 17. doi: 10.3390/ph17040418 38675379 PMC11054947

[293] Isaka Y . Targeting TGF-β signaling in kidney fibrosis. Int J Mol Sci. (2018) 19. doi: 10.3390/ijms19092532 30150520 PMC6165001

[294] Rauchman M Griggs D . Emerging strategies to disrupt the central TGF-β axis in kidney fibrosis. Trans Res J Lab Clin Med. (2019) 209:90–104. doi: 10.1016/j.trsl.2019.04.003 31085163 PMC6850218

[295] Jin Y Ratnam K Chuang PY Fan Y Zhong Y Dai Y . A systems approach identifies HIPK2 as a key regulator of kidney fibrosis. Nat Med. (2012) 18:580–8. doi: 10.1038/nm.2685 22406746 PMC3321097

[296] Hao S He W Li Y Ding H Hou Y Nie J . Targeted inhibition of β-catenin/CBP signaling ameliorates renal interstitial fibrosis. J Am Soc Nephrol JASN. (2011) 22:1642–53. doi: 10.1681/asn.2010101079 21816937 PMC3171936

[297] Zhou L Li Y Hao S Zhou D Tan RJ Nie J . Multiple genes of the renin-angiotensin system are novel targets of Wnt/β-catenin signaling. J Am Soc Nephrol JASN. (2015) 26:107–20. doi: 10.1681/asn.2014010085 25012166 PMC4279741

[298] Essers MA de Vries-Smits LM Barker N Polderman PE Burgering BM Korswagen HC . Functional interaction between beta-catenin and FOXO in oxidative stress signaling. Sci (New York NY). (2005) 308:1181–4. doi: 10.1126/science.1109083 15905404

[299] Almeida M Han L Martin-Millan M O'Brien CA Manolagas SC . Oxidative stress antagonizes Wnt signaling in osteoblast precursors by diverting beta-catenin from T cell factor- to forkhead box O-mediated transcription. J Biol Chem. (2007) 282:27298–305. doi: 10.1074/jbc.M702811200 17623658

[300] Hoogeboom D Essers MA Polderman PE Voets E Smits LM Burgering BM . Interaction of FOXO with beta-catenin inhibits beta-catenin/T cell factor activity. J Biol Chem. (2008) 283:9224–30. doi: 10.1074/jbc.M706638200 18250171

[301] Jin T George Fantus I Sun J . Wnt and beyond Wnt: multiple mechanisms control the transcriptional property of beta-catenin. Cell Signalling. (2008) 20:1697–704. doi: 10.1016/j.cellsig.2008.04.014 18555664

[302] Zheng G Harris DCH . Editorial: TGF-β in human disease: Friend or foe? Front Cell Dev Biol. (2021) 9:739172. doi: 10.3389/fcell.2021.739172 34660597 PMC8514017

[303] Rao P Pang M Qiao X Yu H Wang H Yang Y . Promotion of β-catenin/Foxo1 signaling ameliorates renal interstitial fibrosis. Lab Investigation; A J Tech Methods Pathol. (2019) 99:1689–701. doi: 10.1038/s41374-019-0276-z 31243340

[304] Yang Y Nankivell BJ Hua W Rao P Ren X Yu H . Renal tubular cell binding of β-catenin to TCF1 versus FoxO1 is associated with chronic interstitial fibrosis in transplanted kidneys. Am J Transplant. (2021) 21:727–39. doi: 10.1111/ajt.16287 32870598

[305] Yang Y Feng X Liu X Wang Y Hu M Cao Q . Fate alteration of bone marrow-derived macrophages ameliorates kidney fibrosis in murine model of unilateral ureteral obstruction. Nephrology Dialysis Transplant. (2019) 34:1657–68. doi: 10.1093/ndt/gfy381 30590718

[306] Rao P Qiao X Hua W Hu M Tahan M Chen T . Promotion of β-catenin/Forkhead box protein O signaling mediates epithelial repair in kidney injury. Am J Pathol. (2021) 191:993–1009. doi: 10.1016/j.ajpath.2021.03.005 33753026 PMC8351131

[307] Bourgeois B Gui T Hoogeboom D Hocking HG Richter G Spreitzer E . Multiple regulatory intrinsically disordered motifs control FOXO4 transcription factor binding and function. Cell Rep. (2021) 36:109446. doi: 10.1016/j.celrep.2021.109446 34320339

[308] Lancaster L Cottin V Ramaswamy M Wuyts WA Jenkins RG Scholand MB . Bexotegrast in patients with idiopathic pulmonary fibrosis: The INTEGRIS-IPF clinical trial. Am J Respir Crit Care Med. (2024) 210:424–34. doi: 10.1164/rccm.202403-0636OC 38843105 PMC11351797

[309] Rovin BH Barratt J Heerspink HJL Alpers CE Bieler S Chae DW . Efficacy and safety of sparsentan versus irbesartan in patients with IgA nephropathy (PROTECT): 2-year results from a randomised, active-controlled, phase 3 trial. Lancet (London England). (2023) 402:2077–90. doi: 10.1016/s0140-6736(23)02302-4 37931634

[310] Trachtman H Nelson P Adler S Campbell KN Chaudhuri A Derebail VK . DUET: A phase 2 study evaluating the efficacy and safety of sparsentan in patients with FSGS. J Am Soc Nephrology: JASN. (2018) 29:2745–54. doi: 10.1681/asn.2018010091 30361325 PMC6218860

[311] Agarwal R Green JB Heerspink HJL Mann JFE McGill JB Mottl AK . Finerenone with empagliflozin in chronic kidney disease and type 2 diabetes. N Engl J Med. (2025) 393:533–43. doi: 10.1056/NEJMoa2410659 40470996

[312] Heerspink HJL Birkenfeld AL Cherney DZI Colhoun HM Groop PH Ji L . Finerenone in type 1 diabetes and chronic kidney disease. N Engl J Med. (2026) 394:947–57. doi: 10.1056/NEJMoa2512854 41780000

[313] Malek E Rana PS Swamydas M Daunov M Miyagi M Murphy E . The TGFβ type I receptor kinase inhibitor vactosertib in combination with pomalidomide in relapsed/refractory multiple myeloma: a phase 1b trial. Nat Commun. (2024) 15:7388. doi: 10.1038/s41467-024-51442-2 39191755 PMC11350185

[314] Feng Y Li Z Wang H Liu BC Lee K He JC . HIPK2 C-terminal domain inhibits NF-κB signaling and renal inflammation in kidney injury. JCI Insight. (2024) 9. doi: 10.1172/jci.insight.175153 38512421 PMC11141872

[315] Xu M Fu L Zhang Y Guo X Wu H Gao S . Tubular PFKFB3 drives diabetic kidney fibrosis via lactate-dependent H4K12 lactylation and HIPK2 transactivation. Int J Mol Med. (2026) 58. doi: 10.3892/ijmm.2026.5926 42464649 PMC13384534

[316] Funahashi Y Park SH Hebert JF Eiwaz MB Munhall AC Groat T . Nanotherapeutic kidney cell-specific targeting to ameliorate acute kidney injury. Kidney Int. (2024) 106:597–610. doi: 10.1016/j.kint.2024.06.021 39067856 PMC12711150

